# Targeting Quorum Sensing: High-Throughput Screening to Identify Novel LsrK Inhibitors

**DOI:** 10.3390/ijms20123112

**Published:** 2019-06-25

**Authors:** Viviana Gatta, Polina Ilina, Alison Porter, Stuart McElroy, Päivi Tammela

**Affiliations:** 1 Drug Research Program, Division of Pharmaceutical Biosciences, Faculty of Pharmacy, University of Helsinki, P.O. Box 56, FI-00014 Helsinki, Finland; viviana.gatta@helsinki.fi (V.G.); polina.ilina@helsinki.fi (P.I.); 2 European Screening Centre, Biocity Scotland, Newhouse ML1 5UH, UK; aporter@bioascent.com (A.P.); smcelroy@bioascent.com (S.M.)

**Keywords:** antivirulence agent, bacterial communication, harpagoside, rosolic acid, virulence

## Abstract

Since quorum sensing (QS) is linked to the establishment of bacterial infection, its inactivation represents one of the newest strategies to fight bacterial pathogens. LsrK is a kinase playing a key role in the processing of autoinducer-2 (AI-2), a quorum-sensing mediator in gut enteric bacteria. Inhibition of LsrK might thus impair the quorum-sensing cascade and consequently reduce bacterial pathogenicity. Aiming for the development of a target-based assay for the discovery of LsrK inhibitors, we evaluated different assay set-ups based on ATP detection and optimized an automation-compatible method for the high-throughput screening of chemical libraries. The assay was then used to perform the screening of a 2000-compound library, which provided 12 active compounds with an IC_50_ ≤ 10 µM confirming the effectiveness and sensitivity of our assay. Follow-up studies on the positive hits led to the identification of two compounds, harpagoside and rosolic acid, active in a cell-based AI-2 QS interference assay, which are at the moment the most promising candidates for the development of a new class of antivirulence agents based on LsrK inhibition.

## 1. Introduction

Virulence can be defined as the capability of microorganisms to infect a host by releasing virulence factors or by activating a mechanism which can cause damage. Antivirulence agents are molecules which can interfere with virulence, preventing the infection establishment [1,2]. Disarmed bacteria are then exposed to the attack of the host immune system which can clear the microbes [3]. Since virulence is not essential for bacterial growth and survival, antivirulence agents are considered less prone to resistance development than antibiotics, representing a new frontier to fight bacterial infections [4,5].

Microbial pathogenicity is often dependent on intercellular communication. Bacteria can communicate by releasing and detecting chemical signal molecules called autoinducers (AI). This process, known as quorum sensing (QS), has been linked e.g., to virulence factor production and biofilm formation [6]. Due to the complexity of its regulation and the variety of molecules involved, QS offers a plethora of approaches for developing antivirulence strategies [7,8].

Autoinducer-2 (AI-2), a derivative of (*S*)-4,5-dihydroxy-2,3-pentanedione (DPD), is a quorum-sensing signal molecule in Gram-positive and -negative bacteria. Once AI-2 is internalized, it is phosphorylated by autoinducer-2 kinase LsrK, and only in the phosphorylated form it can bind to the repressor LsrR, enhancing the transcription of *lsr* operon. The operon encodes genes for AI-2 internalization and processing and its overexpression results in the QS cascade activation. Mutants deprived of LsrK have been shown to be unable to establish QS mediated by AI-2, proving the key role of the enzyme [9], and suggesting it as a promising target for the development of antivirulence compounds. 

However, high-throughput screening (HTS) to find LsrK inhibitors has not been performed. LsrK activity has been previously evaluated by thin layer chromatography using [ϒ-^32^P] ATP [10,11], which is not suitable for HTS purposes due to very low throughput and use of a radiolabeled chemical. Small sets of DPD analogues have been tested for LsrK inhibition based on monitoring adenosine 5′-triphosphate (ATP) consumption in the kinase reaction, to evaluate the impact of minor structural changes in DPD for the AI-2 processing [10,12]. However, the described procedure has as intrinsic limitation the low ATP concentration allowed by the selected kit. Furthermore, the assay allowed only limited throughput with 96-well plate format with a final reaction volume of 100 µL and kinetic detection mode. We have also evaluated small sets of potential LsrK inhibitors using a methodology based on monitoring ATP consumption as a biological tool to validate the effectiveness of a new synthetic approach for DPD and its analogue [13,14] or the accuracy of a 3D LsrK model designed for virtual screening [15]. Indeed, due to the limited size of the libraries and the main purposes of the studies, the assay format that we have previously described did not undergo all the characterization which is essential to develop assays for HTS.

In this paper, we describe a new, scalable, and automation-compatible approach for the HTS of chemical libraries to find LsrK inhibitors. Commercially available kits based on ATP quantification, commonly used for kinase inhibitor studies, were first evaluated in order to select an assay set-up fulfilling the requirements for HTS. Reaction conditions were optimized and stability of all the reaction components as well as the signal were assessed to assure suitability for large-scale screening campaigns. Our set-up was then validated by performing a pilot screen of 2000 compounds. The hits were further characterized through a series of follow-up assays, including dose-response, selectivity experiments, and promiscuity analyses. The campaign led to the discovery of 12 compounds with an IC50 ≤ 10 µM, which represent the most potent LsrK inhibitors identified so far.

## 2. Results and Discussion

### 2.1. Assay Selection

LsrK activity has been previously evaluated by using ATP Bioluminescence CLSII kit in kinetic mode [12]. This assay is a bioluminescence-based method for monitoring ATP consumption during kinase reactions. Luciferase in the presence of luciferin, ATP, and oxygen catalyzes the production of oxyluciferin, AMP, PPi, and light. The amount of light is directly proportional to the ATP concentration in the mixture and inversely proportional to the enzymatic activity in the kinase reaction.

We used this assay to confirm the activity of our recombinant LsrK, recording the signal every 2 min for 30 min in presence of 200 nM LsrK, 200 µM DPD and 20 µM ATP (data not shown). Despite the effectiveness of the assay, the short read-out stability (up to 30 min after reaction has ended) represented a considerable limitation for the use of this assay set-up in HTS. 

With the aim to develop an HTS-compatible method, the assay was immediately designed for 384-well plate format with a total volume of 20 µL per well and adapted to automated liquid handling (Biomek FX). Two commercially available kits, Kinase-Glo Max Luminescent kinase assay and ADP-Quest were evaluated. Kinase-Glo Max Luminescent kinase assay is a luminescence-based method which, similarly to ATP Bioluminescence CLSII kit, monitors the ATP level in the reaction mixture after the kinase activity by converting it into light. ADP-Quest is based on monitoring adenosine diphosphate (ADP) concentration by converting ADP, in the presence of H_2_O_2_ and peroxidase, into ADPH. ADPH is then transformed into resorufin, a fluorescent molecule with excitation wavelength of 530 nm and emission of 590 nm. The detected fluorescence is proportional to the ADP amount and directly correlated with the activity of the enzyme.

A preliminary assay was performed with Kinase-Glo Max Luminescent kinase assay to select the optimal concentrations for LrsK and DPD to ensure maximum signal/background (S/B) separation and assay quality measured by the Z’ factor (Z´). S/B and Z´, statistical parameters commonly used in assay development to determine assay quality, were calculated and compared for each tested condition to ensure optimal assay performance. S/B is defined as the ratio of the average of the maximum signal to the minimum signal and values greater than 2 are normally considered acceptable. Z´ factor is a more accurate quality indicator since it takes into account not only the maximum and minimum signals but also their variability. Z´ factor values range up to 1, and 1 corresponds to an ideal assay. Values ≥ 0.5 indicate a good quality assay [16]. Minimum control wells, a mixture of LrsK, DPD, and ATP, yield a low signal. Maximum control wells should contain a reference inhibitor and yield the highest signal. However, since no LsrK inhibitors were available, wells containing only LsrK and ATP were added to the plate to provide maximum signal. The remaining ATP, after addition of luciferase, produces a strong signal which is comparable to the signal that we would expect from the most efficient inhibitor. 

To optimize assay conditions, a matrix of concentrations ranging from 100 to 800 nM LsrK and 100 to 700 µM DPD was evaluated. ATP concentration was fixed at 100 µM to avoid interference between neighboring wells. S/B and Z’ were calculated for each combination (Figure S1). Excellent separation (S/B > 37) and Z’ compatible with HTS screening format (>0.6) were observed for concentrations ≥ 200 µM for DPD and ≥200 nM for LsrK. For further experiments 300 µM DPD and 300 nM LsrK were selected as optimal combination. 

**Figure 1:**
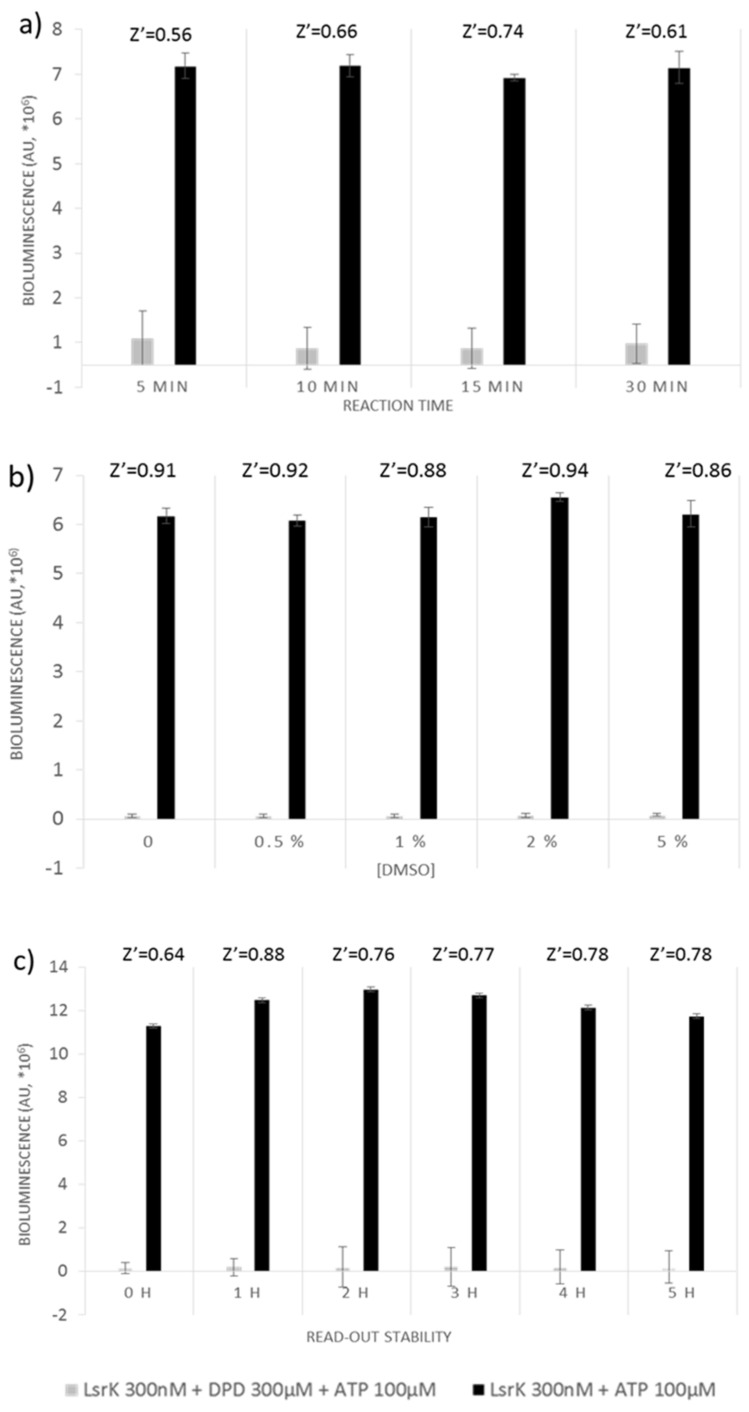
Optimization of LsrK high-throughput screening assay based on the Kinase-Glo Max Luminescent kinase kit: (**a**) effect of different incubation times; (**b**) effect of different DMSO concentration; (**c**) evaluation of the stability of the read-out within a time window of 5 h.

We further determined the optimal reaction time to reduce downtimes without compromising assay quality. Four time points were evaluated by adding the Kinase-Glo Max Luminescent kinase assay reagent after 5, 10, 15 and 30 min incubation of the reaction mixture (Figure 1a). Quality parameters were calculated for each time point. With a Z’ factor of 0.54 the reaction could be considered completed already after 5 min, although it still indicates a certain instability of the signal which disappears at 15 min incubation, when the Z’ factor reached the maximum value of 0.74. 

We also assessed the potential interference of dimethyl sulfoxide (DMSO), common stock solvent used in chemical libraries. DMSO concentrations ranging from 0% to 5% were tested, and did not reveal any significant effects on Z’ factor (0.98 ± 0.02), which remained almost constant for all the tested DMSO concentrations (Figure 1b). 

Stability of the signal was evaluated up to 5 h. Separation between negative and positive control was relatively constant during the experimental time window. The Z’ factor value ranged from 0.88 after 1-h of incubation with the kit reagent to 0.78, recorded after 5 h (Figure 1c).

**Figure 2:**
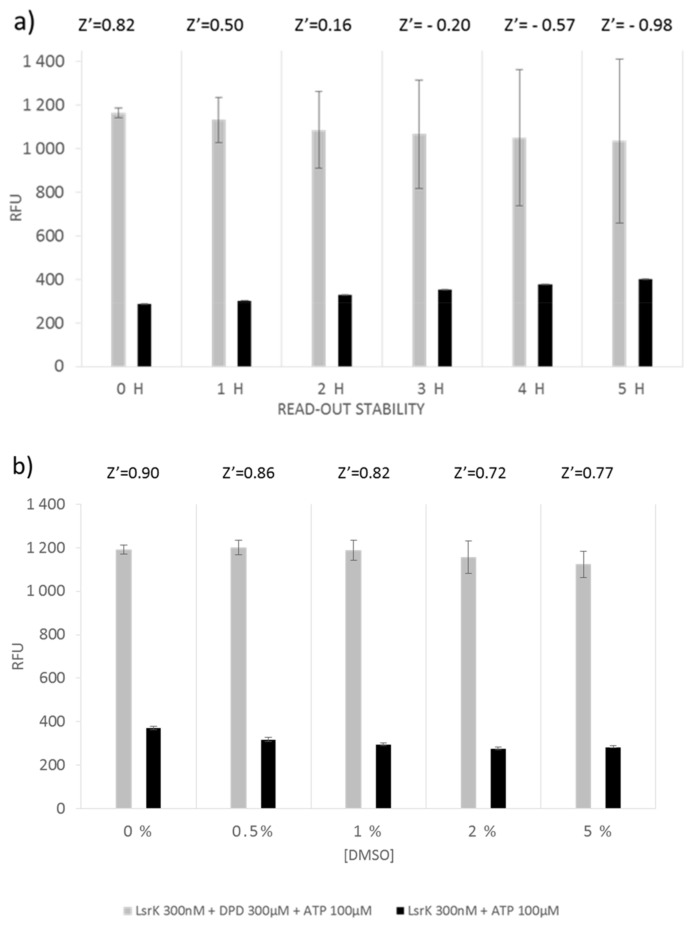
Optimization of LsrK high-throughput screening assay by using the ADP-Quest kit: (**a**) evaluation of the stability of the read-out within a time window of 5 h; (**b**) effect of different DMSO concentration on the reaction.

In addition, we evaluated the performance of the ADP-Quest assay. To assess the signal stability, fluorescence was recorded every h for a time window of 5 h and quality parameters were calculated for each time point (Figure 2a). The Z’ factor dropped below the acceptable value (0.5) after 1 h of incubation with the kit components, offering a fairly short time window for the measurement. As with what was observed for Kinase-Glo Max Luminescent kinase assay, DMSO concentration, even at the highest tested percentage (5%), does not affect the quality of the assay (Figure 2b). However, the observed S/B ratio for ADP-Quest was ~4, indicating only a modest separation when compared to Kinase-Glo Max Luminescent kinase assay.

Considering the excellent S/B and Z’ values, the high tolerance for DMSO, the high stability of the signal even after 5 h and automation-compatible format, Kinase-Glo Max Luminescent Kinase assay was selected as the best option for the HTS of chemical libraries to discover LsrK inhibitors.

The finalized HTS assay protocol with optimized reaction conditions (300 nM LsrK, 300 µM DPD, 100 µM ATP in triethanolamine (TEA) buffer (pH 7.4) supplemented with 200 µM MgCl_2_, 0.1 mg/mL bovine serum albumin (BSA) and 0.01% Triton X-100) was set up as follows: (1) Compound pre-plating by Echo, (2) Addition of LsrK, (3) Incubation of the plate under shaking for 30 min, (4) Addition of DPD, (5) Addition of ATP,(6) Incubation of the plate under shaking for 15 min,(7) Addition of Kinase-Glo Max Luminescent kinase assay kit´s reagent,(8) Incubation of the plate for 30 min, 9) Detection of luminescence.

Reproducibility and data consistency were evaluated by monitoring S/B ratio, S/N and Z’ factors in six independent experiments, yielding mean values 168.99 (±21.80); 55.16 (±23.20) and 0.93 (±0.03), respectively, which demonstrate the high quality of the assay.

### 2.2. Pilot Screening

**Figure 3:**
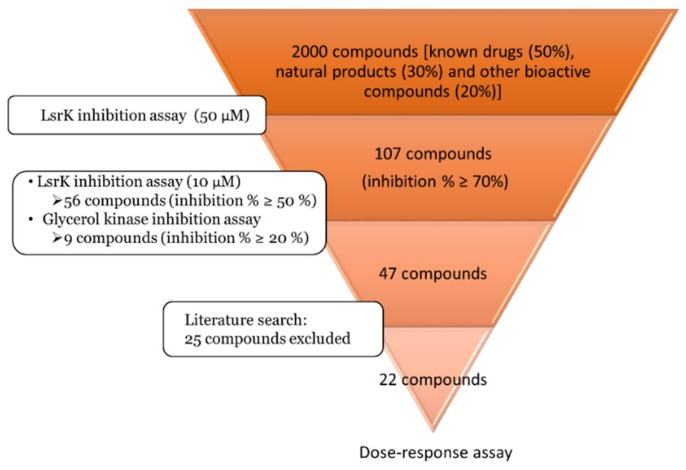
Workflow of the screening campaign.

2000 compounds (known drugs (50%), natural products (30%) and other bioactive compounds (20%)) from MicroSource Spectrum Library were screened at a final concentration of 50 µM (Table S1). HTS hit cut-off > 70% inhibition was applied and yielded 118 active compounds. Based on MicroSource annotations, 11 compounds described as unspecific inhibitors were excluded. The remaining set of 107 compounds was retested against LsrK at lower concentration (10 µM) to assess potency, yielding 56 compounds with inhibition percentage above 50% (Table S2). To estimate selectivity, the same set of 107 hits was tested against bacterial glycerol kinase, a LsrK homologue from the FGGY carbohydrate kinase family (Table S2). The results from the two experiments were compared and 9 hits were excluded as they showed an inhibition percentage > 20% against glycerol kinase. Literature was searched for previously reported activity of the remaining 47 compounds, resulting in the exclusion of 25 compounds known to interfere with kinase activity or antibacterial properties. The campaign provided a final set of 22 compounds which were tested in dose-response assay (Figure 3).

### 2.3. Dose-Response Experiments and Hit Identification

**Figure 4:**
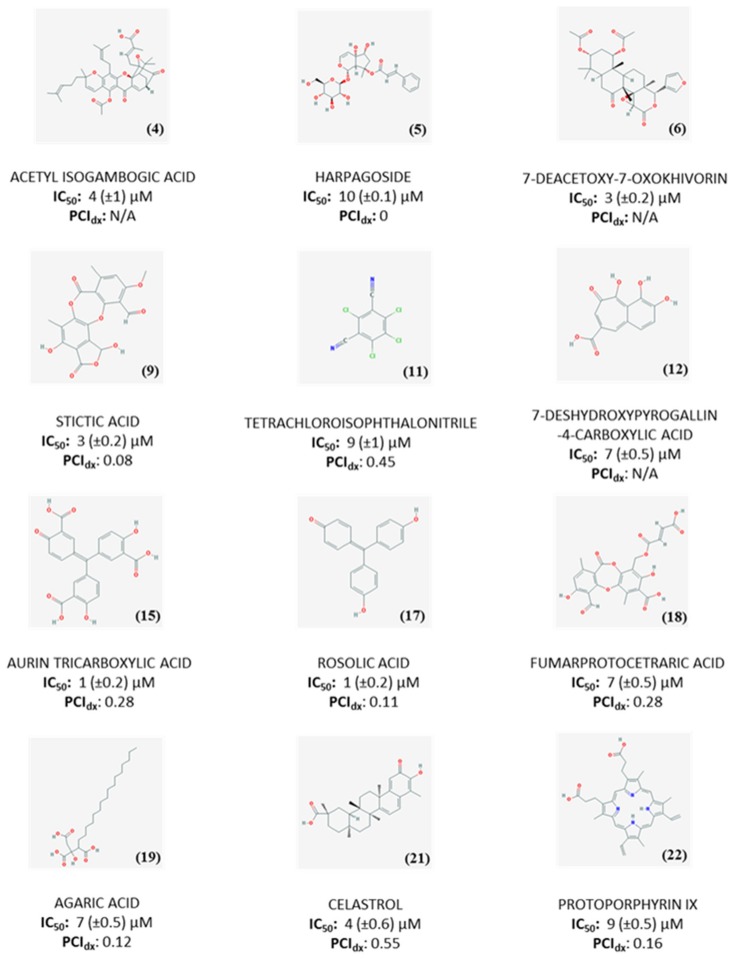
Structures, IC_50_ values and PCIdx for 12 compounds with IC_50_ ≤ 10 µM against LsrK.

Primary and secondary screening resulted in a final list of 22 compounds, which were investigated by dose-response experiments to determine the IC_50_ values (Figure S2, Table S3). 12 compounds showed IC_50_ ≤ 10 µM (Figure 4). The promiscuity index (PCIdx), an indicator of promiscuity based on PubChem activity profile, was calculated according to equation 5 (Figure 4). However, since its significance is strictly related to the amount of reported assays in PubChem, we consider it as a recommendation for further tests of the potentially promiscuous compounds rather than a basis for their exclusion from the follow-up studies [17].

Among the compounds with a PCIdx ranging from 0 to 0.2, harpagoside, a natural product from the plant *Harpagophytum procumbens* which has mainly been studied for its anti-inflammatory [18] and neuroprotective properties [19], was selected for follow-up studies. Rosolic acid and agaric acid were also considered since no interactions with kinases have been previously reported. Limited information is available about rosolic acid, which has only been mentioned for its antioxidant properties [20], whereas agaric acid has been reported as inhibitor of adenine nucleotide carrier at mitochondrial level [21], suggesting hypothetical interference with the ATP processing by LsrK. 

Aurin tricarboxylic acid was also selected for further investigations, despite being flagged as promiscuous due to its involvement in several processes such as prevention of apoptosis, endonuclease, and topoisomerase inhibition, activation of mitogen-activated protein kinase (MAPK) and inhibition of JAK-STAT signaling pathways [22]. The structural similarity with rosolic acid, together with the comparable activity, provides indeed an interesting hint to investigate this structural motif further as a scaffold for the design of new molecules to test as LsrK inhibitors. 

Eight compounds were not selected for follow-up studies. Tetrachloroisophthalonitrile, a well-known fungicidal used in agriculture [23], was excluded due to potential toxicity of its metabolites [24,25]. Celastrol, despite its anti-inflammatory, antitumoral, neuroprotective [26] and antivirulence properties [27] was also discarded, due to poor solubility [28] and reported side-effects [29,30]. Protoporphyrin IX interferes with bacterial growth and biofilm formation when complexed with gallium [31,32]. However, the antibacterial activity seems to be due to the presence of the metal, while the protoporphyrin acts only as a carrier facilitating the incorporation of gallium into heme-containing enzymes with consequent loss of activity [33]. Fumarprotocetraric acid has been classified as promiscuous and, additionally, it has been described as strong antimicrobial agent [34]. 

Due to low number of assays reported in PubChem (0-14), it was not possible to determine PCIdx for acetyl isogambogic acid, 7-deacetoxy-7-oxokhivorin, and 7-deshydroxypyrogallin-4-4carboxylic acid. The lack of data in PubChem, together with the limited literature available, testifies the novelty of these compounds and qualify them as interesting candidates for further investigation and optimization to develop LsrK inhibitors. With its low PCIdx, stictic acid, a known antioxidant agent [35], also represents a promising hit to further investigate. However, follow-up studies for these compounds could not be performed due to their unavailability for re-purchase.

### 2.4. Thermal Shift and MST Assay

Based on the reasons described above, harpagoside, rosolic acid, aurin tricarboxylic acid, and agaric acid were further tested to confirm their binding to LsrK. 10 µM agaric acid caused a significant leftward shift in the *T*_m_ of His-LsrK (Figure S3a,b) of −4.7 ± 0.70 °C (mean ± 95% CI, *n* = 4, unpaired two-tailed t-test where **** *p* < 0.0001). This may be the result of specific binding to a less stable conformer of the enzyme or could indicate that agaric acid acts as a chaotropic agent/aggregator. The results of the MST assay support the latter theory due to the hooked nature of the concentration response (Figure S3c) and the noisy and irregular thermophoretic traces (Figure S3d).

**Figure 5:**
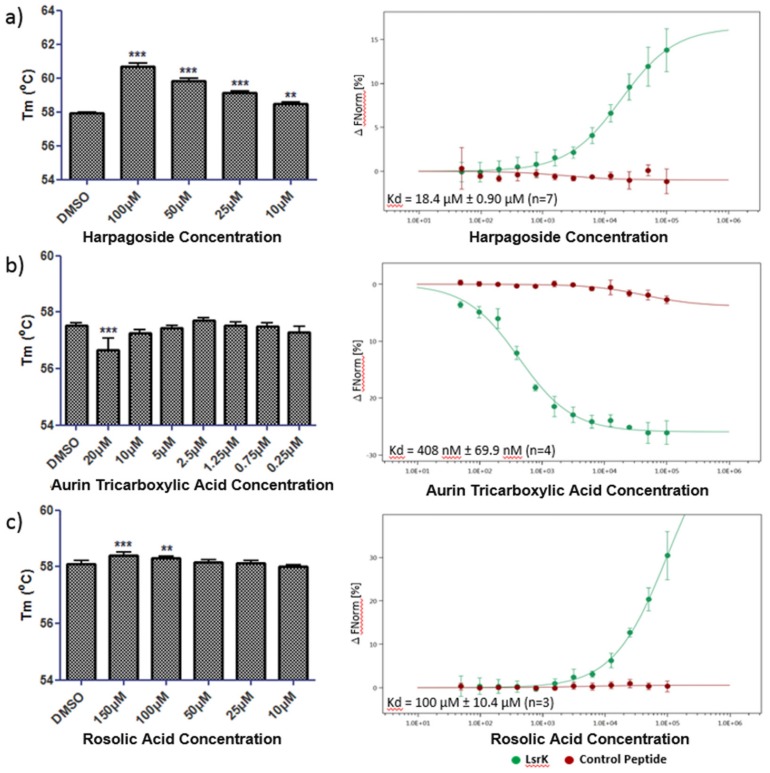
Melting temperature (*T*_m_) for LsrK protein (1 µg/well) in the presence of 1% DMSO or a dose range of test compound (**left** hand panels) where experiments performed *n* = 3 for harpagoside (**a**), *n* = 4 for aurin tricarboxylic acid (**b**) and *n* = 5 for rosolic acid (**c**). Changes in thermophoretic movement for dye labelled His-LsrK protein or His-Control peptide in response to test compounds (**right** hand panels) where experiments performed *n* = 7 for harpagoside, *n* = 4 for aurin tricarboxylic acid and *n* = 3 for rosolic acid.

Figure 5 shows the thermal shift and MST data for (a) harpagoside, (b) aurin tricarboxylic acid and (c) rosolic acid. Harpagoside caused a significant, dose-dependent, positive shift in the T_m_ of His-LsrK (one-way ANOVA where *** *p* < 0.001 and ** *p* < 0.01), indicating a specific interaction with the protein. This was supported by the MST data where a positive change in thermophoretic movement allowed the fitting of a binding isotherm with a *K*_d_ of 18.4 ± 0.90 µM (95% CI, *n* = 7). No binding of harpagoside was observed in MST to either a His-tagged control peptide or sodium dodecyl sulfate (SDS) denatured His-LsrK (Figure S4a) indicating that binding is specific to structurally competent His-LsrK. 

For aurin tricarboxylic acid, a significant shift in the *T*_m_ of His-LsrK was only observed at the highest concentration of 20 µM. The shift was negative; −0.85 ± 0.38 °C (mean ± 95% CI, *n* = 4, one-way ANOVA where *** *p* < 0.001), which could be indicative of aggregation; however this was not supported by the MST data, where no light scattering was observed at this concentration. Indeed, despite the limited activity in thermal shift aurin tricarboxylic acid produced a clear binding isotherm against His-LsrK in MST with a *K*_d_ of 408 ± 69.9 nM (95% CI, *n* = 4). Target specificity was confirmed with no binding to the control peptide or SDS denatured His-LsrK (Figure S4b). 

Discrepancies when profiling compound-target binding with differing biophysical techniques is not unusual, particularly between thermal shift and MST. A screen of 361 fragments against endothiapepsin using four different biophysical techniques (MST, thermal shift, saturation transfer difference spectroscopy nuclear magnetic resonance (STD-NMR), and electrospray ionization mass spectroscopy (ESI-MS)), found a diversity of hit populations with those identified by MST being more distinct than those identified in the other techniques [36]. In another example, an MST fragment screen on BRD9 identified several hits, later validated by NMR, which showed no effect in either thermal shift or SPR [37]. The reasons for these discrepancies are likely many and varied but thermal shift relies upon a stable compound-target complex at high temperature to work, which is likely to be different for compounds with different binding modes. 

Rosolic acid positively stabilizes His-LsrK in thermal shift at high concentrations, suggesting a low affinity binding interaction between the two and this is confirmed by MST in which a partial binding isotherm was obtained with an estimated *K*_d_ of 100 ± 10.4 µM (95% CI, *n* = 3) (Figure 5c). No binding of rosolic acid to the control peptide or SDS denatured His-LsrK was observed (Figure S4c) confirming target specificity.

### 2.5. AI-2 QS Interference Assay

As their binding to LsrK was confirmed, harpagoside, rosolic acid and aurin tricarboxylic acid were selected to be tested in the AI-2 QS interference assay which is designed to evaluate the activity of potential QS inhibitors in cellular environment. The assay is based on *E. coli* LW7 pLW11 which expresses β-galactosidase gene under the control of AI-2 mediated QS responsive *lsr* promoter [38]. The addition of DPD initiates the QS cascade which leads to β-galactosidase production, whereas QS inhibition by a test compound results in the reduction of β-gal expression. 

In the initial experiment, none of the compounds demonstrated QS inhibitory activity at two tested concentrations (50, 10 µM) (Figure S4). However, considering that one of the main obstacles in the discovery of new antimicrobials against Gram-negative bacteria is the efflux pump system, which actively removes various xenobiotics from the cell [39], the assay was repeated in the presence of phe-arg β-naphtylamide dihydrochloride (PAβN), a well-known efflux pump blocking agent [40]. The results showed nearly complete inhibition of AI-2 mediated QS at 50 µM and 68% at 10 µM by rosolic acid. Although less effective, harpagoside showed a still significant inhibitory effect at 50 µM with an inhibition percentage close to 50%, whereas the inhibition at 10 µM was only minor. No significant activity was observed for aurin tricarboxylic acid at both tested concentrations (Figure S5).

**Figure 6:**
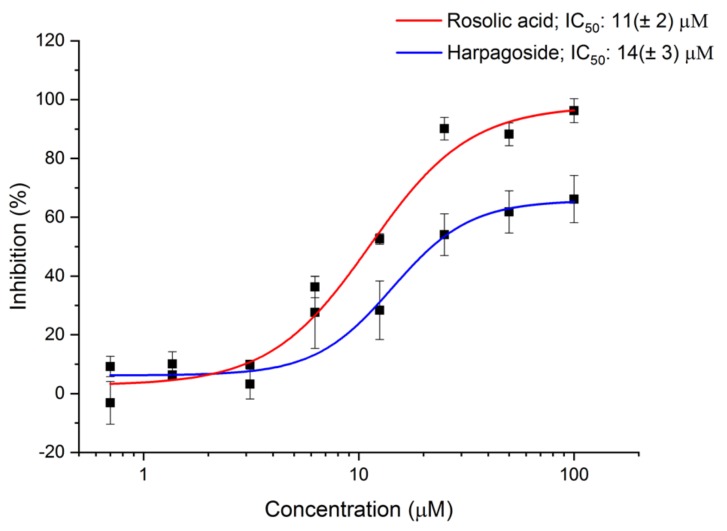
AI-2 QS inhibition by harpagoside and rosolic acid determined by AI-2 QS interference assay in the presence of PAβN. Data points represent means ± SD from two independent experiments (*n* = 3).

The initial results obtained for rosolic acid and harpagoside were further confirmed by a dose-response assay. The IC_50_ values for rosolic acid and harpagoside were 11 (±2) µM and 14 (±3) µM, respectively (Figure 6).

Only 100 µM rosolic acid marginally affected β-gal expression or activity in the control strain, *E. coli* pBAC-lacZ in which *β-gal* gene is placed under control of a QS-independent *lac* promoter, otherwise no significant effect was detected, proving that the compounds specifically interfered with the QS cascade (Figure S6). 

## 3. Materials and Methods 

### 3.1. Materials

*Escherichia coli* MET1158 strain, *E. coli* LW7 pLW11 and *E. coli* pBAC-LacZ were donated by Prof Karina Xavier (Instituto Gulbenkian de Ciência, Portugal), Prof. William Bentley (University of Meriland, USA) and Keith Joung (Addgene plasmid # 13422), respectively. 

All chemicals were purchased from Sigma (USA) if not otherwise stated. DPD was acquired from Carbosynth (Compton, Berkshire, UK). PD-10 desalting columns and Protino^®^ Ni-NTA columns (1 mL) for protein purification were purchased from GE Healthcare Lifescience (Chicago, IL, USA) and Macherey-Nagel (Düren, Germany), respectively. Pierce Coomassie Plus Assay Kit was obtained from Thermo Fisher Scientific (Waltham, MA, USA). ATP Bioluminescence CLS II kit was purchased from Roche Diagnostics GmbH (Basel, Switzerland), Kinase-Glo Max Luminescent kinase assay kit from Promega Corp. (Madison, WI, USA) and ADP-Quest kit from DiscoveRx Corp. (Fremont, CA, USA). PopCulture™reagent and rLysozyme™ were purchased from Millipore (Burlington, MA, USA). Plates were purchased from Greiner Bio One (KremsMünster, Austria), for assay development and screening campaign, and from Thermo Fisher Scientific (Waltham, MA, USA) for AI-2 QS interference assay. 

### 3.2. Overexpression and Purification of LsrK from S. thyphmurium 

LsrK from *S. typhimurium* was overexpressed in *E. coli* by using MET1158 strain (*E. coli*, amp resistance, BL21 (DE3) luxS-, with pMET1144 (LsrK-His in pET21b)) [11]. Bacteria were grown in YTPG (yeast, tryptone, phosphate buffer and glucose) medium supplemented with 100 µg/mL ampicillin at 18 °C (250 rpm) until exponential phase (OD 0.2–0.4). Induction was initiated by adding 0.1 mM isopropyl-β-d-thiogalactoside (IPTG) and continued for 9 h at 22 °C (250 rpm). Cells were isolated by centrifugation at 4 °C (4000 rpm) and frozen overnight. 

The pellet was incubated in lysis buffer containing 25 mM potassium phosphate pH 7.1, 10 mM β-mercaptoethanol, 50 mM NaCl, 1 mM MgCl_2_, 1:100 protease inhibitors cocktail, 150 µg/mL lysozyme for 1 h and then sonicated with 10 short burst of 10 s followed by intervals of 50 s for cooling (VC 500 Vibra-Cell, Sonics & Materials (Newton, CT, USA)). The supernatant was purified by Ni-nitriloacetic acid chromatography using 50 mM potassium phosphate pH 7.1, 250 mM imidazole, 20 mM β-mercaptoethanol, 50 mM NaCl, 1 mM MgCl_2_ as the elution buffer. The purified protein was transferred to 25 mM potassium phosphate, pH 7.1, 1 mM dithiothreitol (DTT), 1 mM MgCl_2_ via size-exclusion chromatography with Sephadex-G25 resin in PD-10 desalting columns.

Purity of the expressed protein was confirmed by SDS-PAGE and concentration determined by the Bradford assay, using the Coomassie Plus kit according to manufacturer’s instructions. LsrK, at final concentration of 3 mg/mL, was aliquoted and stored at −80 °C.

### 3.3. Assay Development

#### 3.3.1. ATP Bioluminescence CLS II Assay

LsrK activity was initially confirmed in 96-well plate format using the ATP Bioluminescence CLS II kit in kinetic mode as previously described [10,12]. The reaction mixture included 200 nM LsrK, 200 µM DPD and 20 µM ATP, added last to start the reaction. Assay buffer consisted of 25 mM triethanolamine (TEA), pH 7.4, 200 µM MgCl_2_ and 0.1 mg/mL BSA. After ATP addition, 50 µL of kit reagent were added and luminescence was recorded with Varioskan LUX (Thermo Fisher Scientific, Vantaa, Finland) every 2 min for 30 min to monitor the reaction.

#### 3.3.2. Assay Miniaturization and Optimization for HTS

The assay was miniaturized to 384-well plate format in 20-µL final volume and performed in end-point mode. Two commercially available kits based on different detection technologies were evaluated to select the most suitable for HTS.

##### Kinase-Glo Max Luminescent Kinase Assay

LsrK and DPD were diluted in optimized assay buffer containing 25 mM TEA, pH 7.4, 200 µM MgCl_2_, 0.01% TritonX-100 and 0.1 mg/mL BSA. In the optimized assay, the final concentration on the plate was 300 nM LsrK and 300 µM DPD. 100 µM ATP was added last to start the reaction. After 15-min reaction time, 20 µL of kit reagent were added. The plate was incubated for 15 min and luminescence signal was measured by Varioskan LUX.

##### ADP-Quest

Reaction mixture including 300 nM LsrK, 300 µM DPD and 100 µM ATP, diluted in assay buffer, was incubated for 15 min followed by the addition of 10 µL of reagent A and 20 µL of reagent B from the kit. Assay was carried out in 384-well plate format. Fluorescence (ex 530 nm, em 590 nm) was detected by Varioskan LUX after 30-min incubation.

### 3.4. Assay Validation

The optimized assay was validated by screening of the MicroSource Spectrum Library containing 2000 bioactive compounds. The library was acquired from FIMM (Institute for Molecular Medicine Finland), University of Helsinki. 100 nl from 10 mM stock solution in 100% DMSO were plated in singles at FIMM by an Echo acoustic dispenser into 384-well plates to yield a final concentration of 50 µM in the assay. 

Maximum (8 wells containing only LsrK and ATP) and minimum controls (8 wells containing LsrK, DPD, and ATP) were placed on each plate in columns 1 and 2. The final DMSO concentration in the assay was 0.5%, also present in all controls.

The following assay steps were performed on Biomek FX (Beckman Coulter, Brea, CA, USA). 10 µL of 600 nM LsrK stock solution in assay buffer was added to each well to yield final concentration of 300 nM LsrK. The plate was incubated for 30 min under shaking at room temperature RT to dissolve the compounds. 5 µL of 1.2 mM DPD stock solution in assay buffer was added to the plate followed by 5 µL of 5 mM ATP stock solution in assay buffer. DPD and ATP final concentration on the plate were 300 µM and 100 µM, respectively. The reaction was carried out at RT under shaking for 15 min and then 20 µL of kit reagent was added. After 30-min incubation, luminescence was measured by Varioskan LUX. 

### 3.5. Glycerol Kinase Inhibition Assay

Compounds were plated in single at FIMM by an Echo acoustic dispenser into 384-well plates to yield a final concentration of 50 µM in the assay. The assay was carried out as previously described for LsrK with a reaction mixture including 0.3 U/mL glycerol kinase from *E. coli*, 100 μM ATP, and 300 μM glycerol.

### 3.6. Thermal Shift Assay

Compounds were added to qPCR plates by Echo acoustic dispenser with a DMSO backfill to maintain a constant final concentration of DMSO in each well (the assay is tolerant up to 8% DMSO with no effect on T_m_ of LsrK). LsrK protein was added at a concentration of 1 µg per well in a volume of 10 µL with 1× Thermo thermal shift dye or 5× Sypro Orange dye (used for aurin tricarboxylic acid as this compound fluoresces with Thermo dye in 25 mM TEA, 200 µM MgCl_2_, pH 7.1). Appropriate DMSO-only negative controls and 1 mM ATP/DMSO positive controls were included in each test occasion. Using an Applied Biosystems Quant Studio 5 qPCR machine (Thermo Fisher Scientific, Waltham, MA, USA) the following thermal shift protocol was used: step 1—hold at 25 °C for 1 min; step 2—heat at 0.05 °C/s dissociation then hold at 99 °C for 1 min. Filter setting for Thermo dye: Ex/Em 580/623 nm and for Sypro Orange dye: Ex/Em 470/520 nm.

### 3.7. MST Assay

His-tagged LsrK (His-LsrK) was labelled with red fluorescent dye NT-647 using a Monolith NT™ His Tag labelling kit—Red-tris-NTA. Briefly 200 nM His-LsrK in 50 mM TEA, 400 µM MgCl_2_, pH 7.1 was combined with equivalent volume of 100 nM red NT-647 dye prepared in PBS-T buffer (supplied with the kit) and this was incubated at room temperature for 30 min in the dark. The solution was centrifuged at 15,000× *g* for 5 min at 4 °C, resulting in 100 nM red NT-647 dye labelled His-LsrK. 200 nM His tagged control peptide (supplied with the labelling kit) was labelled in a similar manner except that PBS-T buffer was used in all steps.

MST assays were performed in standard capillaries for both labelled protein and control peptide in 1× Assay Buffer (25 mM TEA, 200 µM MgCl_2_, pH 7.1 supplemented with 0.1% Pluronic F127). Red fluorescence (Excitation, 650nm: Emission, 670–690nm) was measured for a series of LsrK and control peptide concentrations to confirm that labelling was successful. 30 nM labelled His-LsrK or control peptide were found to give sufficient signal of 250 to 300 fluorescence units at 40% MST power and 40% excitation power. For compound concentration responses, 2× 12-point 1 in 2 concentration series were prepared in 1× assay buffer containing 2% DMSO in Greiner black low volume non-binding surface (NBS) plates (#784900). An equivalent volume of 60 nM (2×) labelled His-LsrK or control peptide, prepared in 1× assay buffer was then added before being loaded into standard capillaries for reading (1% DMSO final). 

### 3.8. AI-2 QS Interference Assay

Quorum-sensing inhibition was evaluated by using the bioreporter strain *E. coli* LW7 pLW11. The assay was carried out as previously described [38]. Compounds were dissolved in phosphate buffer saline (PBS) with or without 100 µg/mL phe-arg β-naphtylamide dihydrochloride (PAβN) and plated in triplicate on a 96-well plate to a final concentration of 50 or 10 µM. The same protocol was applied to a control strain *E. coli* pBAC-LacZ, which expresses the β-galactosidase gene under control of the QS-independent *lac* promoter. Active compounds were then retested in a dose-response experiment at 8 different concentrations (0.7–100 µM)

### 3.9. Data Analysis

During the assay development, optimization, and validation, the assay performance was evaluated by calculating quality parameters typically used in HTS, Z’, S/N and S/B according to the following equations [16,41]:

Z’ = 1 − (3 × σM + 3 × σm)/(|µM − µm|)(1)

S/N = (µM − µm)/SQRT[(σM)^2^ + (σm)^2^)] (2)

S/B = µM/µm(3)

In the equations µM and σM represent respectively the average and the standard deviation for the maximum signal, given by the enzyme and ATP. µm and σm represent average and standard deviation for the minimum signal, from a sample with LsrK, ATP, and DPD. Good quality assays are indicated by Z’ factor > 0.5. 

The inhibition % for each tested compound was determined according to the following equation:

Inhibition% = 100 × [(*X* − *X*m)/(*X*M − *X*m)] (4)

In the equation, *X* represents the detected luminescence value from a sample, while XM and Xm are respectively the average of the detected luminescence value for the maximum and minimum controls. 

The promiscuity index (PCIdx), based on PubChem data, was calculated according to the following equation [17]:

PCIdx(substance) = *N*(active)/*N*(tested) (5)

N (active) includes the number of assays reported in PubChem where the considered compound has been qualified as active. *N*(tested) is a sum of the tests performed at a single concentration (method “screening”) and dose-response assays (method “confirmatory”). 

In the AI-2 QS interference assay, inhibition was calculated according to the following equation:

Inhibition%= 100 – remaining activity%(6)

Wherein remaining activity has been determined as follows:

Remaining activity% =100 × [MUs(DPD+) − MUc(DPD−)]/[MUc(DPD+) − MUc(DPD−)](7)

In the equation MUs and MUc respectively correspond to the β galactosidase activity expressed in Miller Unit (MU) calculated for the sample and the DMSO control, with (DPD+) and without DPD (DPD−) [38].

## 4. Conclusions

Quorum-sensing inhibition is one of the most promising strategies to face the threat of bacterial resistance. Due to its complex regulation, many targets and approaches are under investigation for the development of quorum-sensing inhibitors.

In this manuscript, we describe the optimization and validation of a new scalable and automation-compatible assay, allowing HTS to identify inhibitors of LsrK, one of the key enzymes in AI-2-mediated QS.

Two different set-ups were evaluated: Kinase-Glo Max Luminescent kinase assay and ADP-Quest. Both methods are easily scalable, robust, cost-effective, and significantly overcome the limitations of previously described assays for this purpose. However, the Kinase-Glo Max Luminescent kinase assay is more beneficial in large screening campaigns since it requires only one reagent addition by liquid handling workstation, offering a fast and simple mix-and-read method. It also generates a highly stable signal offering a wide time window for the detection of luminescence. ADP-Quest is, however, an important orthogonal assay option for hit confirmation phase.

The established HTS assay set-up was used to perform a screening campaign of 2000 compounds including known drugs (50%), natural products (30%) and other bioactive compounds (20%) which provided a set of 12 compounds with an IC_50_ values ≤ 10 µM. Guided by PCIdx and available literature, we selected harpagoside, rosolic acid, aurin tricarboxylic acid, and agaric acid for follow-up studies to investigate target engagement. Since their binding to LsrK was proven by thermal shift or MST, harpagoside, rosolic acid and aurin tricarboxylic acid were tested in a cell-based assay to evaluate their ability to inhibit QS pathway in a genetically modified *E. coli* expressing β-galactosidase gene under control of *lsr* promoter. Rosolic acid and harpagoside were strongly inhibiting the AI-2 mediated QS in the presence of PAβN. With an IC_50_ in the low µM range, these compounds are the first LsrK inhibitors to show activity in a cell-based assay. Although the need for efflux pump inhibitor is a great limitation for further applications, these compounds are at the moment the best starting points for the development of a new class of antivirulence agents based on LsrK inhibition. In fact, further studies will be needed to determine the structural motif, which directly interacts with the protein. This could help to design and synthesize analogues with increased potency feasible to more profound follow-up studies in cellular environment.

## Supplementary Materials

Supplementary materials can be found at https://www.mdpi.com/1422-0067/20/12/3112/s1.

## Abbreviations

QS	Quorum sensing
AI-2	Autoinducer 2
DPD	(*S*)-4,5-dihydroxy-2,3-pentanedione
LsrK	Autoinducer-2 kinase
HTS	High-throughput screening
TEA	Triethanolamine
BSA	Bovine serum albumin
DTT	Dithiothreitol
ATP	Adenosine 5′-triphosphate
DMSO	Dimethyl sulfoxide
ADP	Adenosine diphosphate
PcIDx	Promiscuity index
MST	Microscale thermophoresis
PAβN	phe-arg β-naphtylamide dihydrochloride
