# Supplementary material for: Targeting Quorum Sensing: High-Throughput Screening to Identify Novel LsrK Inhibitors
Source: Int J Mol Sci. 2019 Jun 25;20(12):3112. doi: 10.3390/ijms20123112 (PMC6627609; doi:10.3390/ijms20123112)
Supplement: Supplementary file 1 [file ijms-20-03112-s001.pdf]

Supporting information

Targeting Quorum Sensing: High-Throughput Screening to Identify Novel LsrK Inhibitors

Viviana Gatta [1], Polina Ilina[1], Alison Porter [2, #], Stuart McElroy [2,#] and Päivi Tammela [1,*]

1 Drug Research Program, Division of Pharmaceutical Biosciences, Faculty of Pharmacy, University of Helsinki, P.O. Box 56, FI-00014 Helsinki, Finland; viviana.gatta@helsinki.fi (V.G.), polina.ilina@helsinki.fi (P.I.), paivi.tammela@helsinki.fi (P.T.)
2 European Screening Centre, Biocity Scotland, Newhouse, ML1 5UH, UK; a.j.porter@dundee.ac.uk (A.P.), s.mcelroy@dundee.ac.uk (S.M.)
* Correspondence: paivi.tammela@helsinki.fi; Tel.: +358-50-448 0886
\# Current address: BioAscent Discovery Ltd., Newhouse ML1 5UH, UK; aporter@bioascent.com (A.P.); smcelroy@bioascent.com (S.M.)

Table of contents:

Assay development…………………………………………………………………………………...page 2

Results from primary screening………………………………………………………………………..page 2-85

Dose-response experiments……………………………………………………………….............page 86-89

Thermal shift assay and MST assay………………………………………………………………page 90-91

AI-2 quorum sensing interference activity…………………………………………………………page 92

a)

S/B<br>DPD (µM)	LsrK (nM)<br>0	100	200	300	400	500	600	700	800
0									
100		1,51	3,68	5,11	9,23	6,14	6,57	5,78	6,12
200		3,07	37,55	53,49	64,74	43,99	52,70	49,41	44,74
300		4,14	47,89	69,04	101,14	59,09	59,99	58,49	49,07
400		7,59	51,31	64,60	84,05	49,28	50,10	48,83	79,61
500		11,44	57,89	60,75	107,87	52,80	60,20	46,87	48,12
600		10,94	50,06	49,75	52,36	44,32	53,44	52,47	55,47
700		4,73	46,31	47,67	30,39	48,26	53,11	74,52	49,65

b)

Z'<br>DPD (µM)	LsrK (nM)<br>0	100	200	300	400	500	600	700	800
0									
100		-1,22	0,41	0,42	0,91	0,41	0,69	0,34	0,31
200		-0,10	0,85	0,84	0,91	0,80	0,90	0,72	0,70
300		0,03	0,86	0,83	0,91	0,81	0,90	0,72	0,70
400		0,19	0,86	0,83	0,91	0,80	0,90	0,71	0,71
500		0,30	0,87	0,82	0,89	0,81	0,89	0,70	0,69
600		0,30	0,87	0,82	0,91	0,79	0,90	0,71	0,70
700		0,07	0,85	0,81	0,89	0,80	0,89	0,73	0,70

Figure S1: Optimization of LsrK and DPD concentrations: (a) S/B and (b) Z′ calculated for each tested combination of LsrK and DPD.

Table S1. LsrK inhibition results from primary screening of the MicroSource Spectrum Library. Bioactivity and source refer to information provided as MicroSource annotations. Compounds were tested in singles at 50 µM, and sorted in the table according to their inhibitory effect.

Microsource ID	Compound´s name:	Formula:	Molecular Weight:	Bioactivity:	Source:	Inhibition (%):
00300147	USNIC ACID	C18H16O7	344.32	antibacterial	*Usnea. Cladonia* and other lichen spp	204
00201507	2'.2'-BISEPIGALLOCATECHIN DIGALLATE	C44H34O22	914.74		tea pigment	170
01503941	THIOCTIC ACID	C8H14O2S2	206.32	hepatoprotectan t	synthetic; alpha-lipoic acid	170
01505775	AGARIC ACID	C22H40O7	416.55	antiperspirant	*Fomes and Polyporus spp*	157
00310016	CHAULMOOGRIC ACID	C18H32O2	280.45	antibacterial (mycobacteria)	*Hydnocarpus and Oncola spp*	156
01505390	NISOLDIPINE	C20H24N2O6	388.42	vasodilator (coronary)	synthetic; bayk-5552	154
01500262	DISULFIRAM	C10H20N2S4	296.54	alcohol antagonist	synthetic	154
01502259	MORIN	C15H10O7	302.24	P450 and ATPase inhibitor	*Chlorophora tinctoria*	154
00200054	FUMARPROTOCETRARIC ACID	C22H16O12	472.36		*Cetraria islandica*	150
01505707	TEICOPLANIN [A(2-1) shown]	C88H95Cl2N9O 33	1877.68	antibacterial	*Actinoplanes teichomyceticus ; MDL-507*	148
01503074	ALEXIDINE HYDROCHLORIDE	C26H58Cl2N10	581.72	antibacterial	synthetic	146

00201716	NORSTICTIC ACID	C18H12O9	372.29	antibacterial	*Lobaria pulmonaria. Usnea japonica. Lecanora radiosa. Parmelia & Ramalina spp*	145
01500572	THIMEROSAL	C9H9HgNaO2S	404.81	antiinfective. preservative	synthetic	141
01505164	4.4'-DIISOTHIOCYANOSTILBENE-2.2'-SUFONIC ACID SODIUM SALT	C16H8N2Na2O6S4	498.48	ATP transport inhibitor. anion transport inhibitor. antiulcer	synthetic; DIDS	141
00300549	ACETYL ISOGAMBOGIC ACID	C40H46O9	670.80		derivative	141
01503223	PARAROSANILINE PAMOATE	C42H33N3O6	675.74	anthelmintic. antischistosomal	synthetic	138
01505682	TOREMIPHENE CITRATE	C32H36ClNO8	598.09	antineoplastic. anti-estrogen	synthetic; FC-1157a	138
01504101	TETRACHLOROISOPHTHALONITRILE	C8Cl4N2	265.91	antifungal	synthetic	138
00100048	7-DEACETOXY-7-OXOKHIVORIN	C30H38O9	542.63		*Khaya senegalensis and other Meliaceae; mp 225*	133
00200012	BRAZILIN	C16H14O5	286.28		*Haematoxylin campechianum*	133
00200111	THEAFLAVIN	C29H24O12	564.50	antioxidant	pigment in black tea	133
01500169	CETYLPYRIDINIUM CHLORIDE	C21H38ClN	339.99	antiinfective (topical)	synthetic	132
01500268	DYCLONINE HYDROCHLORIDE	C18H28ClNO2	325.88	anesthetic (topical)	synthetic	131
00200090	OBTUSAQUINONE	C16H14O3	254.28		*Dalbergia retusa*	130
00210242	THEAFLAVIN MONOGALLATES	C36H28O16	716.61		mixed isomers black tea (3' shown)	129
01500554	SULFINPYRAZONE	C23H20N2O3S	404.49	uricosuric	synthetic	129
01504030	beta-ESCIN	C55H86O24	1131.28	membrane permeabilizer	principal saponin *Aesculus hippocastanum*	129
01500252	DIMERCAPROL	C3H8OS2	124.22	chelating agent (As. Au. Hg antidote)	synthetic	125
01503200	CETRIMONIUM BROMIDE	C19H42BrN	364.45	antiinfectant	synthetic	125
01500345	HYDROXYZINE PAMOATE	C44H43ClN2O8	763.29	anxiolytic. antihistaminic	synthetic	125
01500365	LEVONORDEFRIN	C9H13NO3	183.20	vasoconstrictor	synthetic	123
01503118	TRIFLUPROMAZINE HYDROCHLORIDE	C18H20ClF3N2S	388.88	antipsychotic	synthetic	122
01503610	BENZALKONIUM CHLORIDE	C22H40ClN	354.02	antiinfective (topical)	synthetic	121
01500637	MERBROMIN	C20H8Br2HgNa2O6	750.66	antibacterial	synthetic	119
01500762	ROSOLIC ACID	C19H14O3	290.32	diagnostic aid	synthetic	119
00300006	STICTIC ACID	C19H14O9	386.31		numerous lichens. e.g. *Parmelia sp.*	119

01600919	3-METHOXYCATECHOL	C7H8O3	140.14		*Machaerium kuhlmanni*	118
00200422	KOPARIN	C16H12O6	300.27		*Castanospermum australe*	118
01500450	OXIDOPAMINE HYDROCHLORIDE	C8H12ClNO3	205.64	adrenergic agonist (opthalmic)	synthetic	117
01505825	DIHYDROTANSHINONE I	C18H14O3	278.31		*Salvia miltiorrhiza*	115
01505143	GOSSYPETIN	C15H10O8	318.24		widespread in plants	115
00211012	IRIGINOL HEXAACEATATE	C27H22O14	570.46		semisynthetic	114
01500566	TETRACYCLINE HYDROCHLORIDE	C22H25ClN2O8	480.90	antibacterial. antiamebic. antirickettsial	*Streptomyces spp*	114
00201505	METHYL 7-DESHYDROXYPYROGALLIN-4-CARBOXYLATE	C13H10O6	262.22		synthetic	112
01503135	THONZYLAMINE HYDROCHLORIDE	C16H23ClN4O	322.84	antihistamine	synthetic	112
01500328	HEXACHLOROPHENE	C13H6Cl6O2	406.90	antiinfective (topical)	synthetic	112
01500636	MECYSTEINE HYDROCHLORIDE	C4H10ClNO2S	171.64	mucolytic	synthetic	112
00240673	ROBUSTIC ACID	C22H20O6	380.40		*Derris robusta*	111
01502252	MONOCROTALINE	C16H23NO6	325.36	antineoplastic. insect sterilant	*Crotalaria spp*	111
01500148	BITHIONATE SODIUM	C12H4Cl4Na2O2S	400.02	anthelmintic. antiseptic	synthetic	111
00200412	2.3.4'-TRIHYDROXY-4-METHOXYBENZOPHENONE	C14H12O5	260.24		derivative	111
01503117	TRIMIPRAMINE MALEATE	C24H30N2O4	410.51	antidepressant	synthetic	111
01502032	SURAMIN	C51H34N6Na6O23S6	1429.18	antiprotozoal. trypanocidal. antiviral	synthetic; Bayer-205. 309F	110
01500226	DEMECLOCYCLINE HYDROCHLORIDE	C21H22Cl2N2O8	501.32	antibacterial	*Streptomyces aureofaiens*	109
00201664	CELASTROL	C29H38O4	450.62	antineoplastic. antiinflamatory. NO synthesis inhibitor. chaperone stimulant	*Celastrus scandens & Tripterygium wilfordii*	109
01505151	HARPAGOSIDE	C23H28O11	480.47		*Melittis melissophyllum. Harpagophytum procumbens*	108
01502253	HEMATEIN	C16H12O6	300.27		*logwood. Haematoxylon spp.*	107
00201508	7-DESHYDROXYPYROGALLIN-4-CARBOXYLIC ACID	C12H8O6	248.19		synthetic	107
00240565	5.7-DIHYDROXYISOFLAVONE	C15H10O4	254.24		*Arachis hypogaea & Derris spp*	106
01505971	BENZBROMARONE	C17H12Br2O3	424.09	uricosuric	synthetic; MJ-10061. L-2214	106
01500266	DOXYCYCLINE HYDROCHLORIDE	C22H25ClN2O8	480.90	antibacterial	semisynthetic; GS-3065	106

01504082	DIHYDROCELASTROL	C29H40O4	452.63		celastrol derivative	105
01505993	MESALAMINE	C7H7NO3	153.13	antiinflammatory	synthetic; 5-ASA	105
01505207	CEFTIBUTEN	C15H14N4O6S2	410.43	antibacterial	semisynthetic	105
01505308	CHLOROPHYLLIDE Cu COMPLEX Na SALT	C34H28CuN4Na2O5	682.15	antineoplastic	water soluble derivative of chlorophyll	104
01503322	THIRAM	C6H12N2S4	240.43	antifungal	synthetic	104
01500132	AUROTHIOGLUCOSE	C6H11AuO5S	392.18	antirheumatic	synthetic	104
00300010	AGELASINE (stereochemistry of diterpene unknown)	C26H40ClN5	458.09	cytotoxic. antineoplastic	*Agelas dispar*	102
01505142	2'.5'-DIHYDROXY-4-METHOXYCHALCONE	C16H14O4	270.28		*Cassia javanica*	102
01500517	PYRANTEL PAMOATE	C34H30N2O6S	594.69	anthelmintic	synthetic	101
00210515	PYROGALLIN	C11H8O4	204.18	antiinfectant	synthetic	101
01504261	CANDESARTAN CILEXTIL	C33H34N6O6	610.67	angiotensin 1 receptor antagonist	synthetic	101
01501111	PROTOPORPHYRIN IX	C34H34N4O4	562.67	hepatoprotectant	mamalian feces. avian pigment	100
01500138	BENZETHONIUM CHLORIDE	C27H42ClNO2	448.09	antiinfective (topical)	synthetic	100
01503904	PATULIN	C7H6O4	154.12	antibacterial	*Aspergillus clavatus. Penicillium patulum*	99
00100013	3-DEACETYLKHIVORIN	C30H40O9	544.64		*Meliaceae spp*	99
00201448	4.4'-DIMETHOXYDALBERGIONE	C17H16O4	284.31		*Dalbergia nigra*	98
01500431	NIFEDIPINE	C17H18N2O6	346.34	antianginal. antihypertensive	synthetic	98
01300042	SODIUM TETRADECYL SULFATE	C14H29NaO4S	316.43	sclerosing agent	synthetic	98
00300018	LOBARIC ACID	C25H28O8	456.49		lichens of the genus *Stereocaulon* and others	98
01501104	METHACYCLINE HYDROCHLORIDE	C22H23ClN2O8	478.89	antibacterial	semisynthetic	97
01501198	TOLFENAMIC ACID	C14H12ClNO2	261.71	antiinflammatory. analgesia	synthetic	97
01500137	BENSERAZIDE HYDROCHLORIDE	C10H16ClN3O5	293.70	decarboxylase inhibitor	component of Madopa (Hoffmann-LaRoche)	97
00201515	THEAFLAVIN DIGALLATE	C43H32O20	868.72		pigment in black tea	96
01505163	AURIN TRICARBOXYLIC ACID	C22H14O9	422.35	apoptosis inhibitor. topoisomerase II inhibitor	synthetic	96
01505412	BISMUTH SUBSALICYLATE	C7H5BiO4	362.09	antidiarrheal. antacid. antiulcer	synthetic	94
01500457	OXYTETRACYCLINE	C22H25ClN2O9	496.90	antibacterial	*Streptomyces rimosus*	94
01504105	TANNIC ACID	C76H52O46	1701.24	nonspecific enzyme/receptor blocker	principal constituent of	94

					tree galls. esp Quercus spp;	
01500455	OXYPHENBUTAZONE	C19H20N2O3	324.38	antiinflammatory	synthetic	93
01501118	MECLOCYCLINE SULFOSALICYLATE	C29H27ClN2O14S	695.06	antibacterial	semisynthetic; GS-2989. NSC-78502	93
00201281	DALBERGIONE	C15H12O2	224.26		*Dalbergia spp*	91
01500838	CHOLECALCIFEROL	C27H44O	384.65	vitamin D3	fish oils	90
00201182	IRIGENOL	C15H10O8	318.24		Iris spp	90
01502020	FOLIC ACID	C19H19N7O6	441.40	hematopoietic vitamin	liver. kidney. green plants and fungi	90
00200798	DALBERGIONE. 4-METHOXY-4'-HYDROXY-	C16H14O4	270.28		*Dalbergia spp*	90
00205113	EPIGALLOCATECHIN	C15H14O7	306.27		green tea	89
01500500	PRIMAQUINE DIPHOSPHATE	C15H27N3O9P2	455.34	antimalarial	synthetic	89
00200007	GAMBOGIC ACID	C38H44O8	628.76	antiinflammatory. cytotoxic. inhibits HeLa cells in vitro; LD50(rat) 88 mg/kg ip	*Garcinia morella. G hanburyii*	89
01503253	METHYLBENZETHONIUM CHLORIDE	C28H44ClNO2	462.12	antiinfective	synthetic	89
00210505	PURPUROGALLIN	C11H8O5	220.18	xanthine oxidase inhibitor. antioxidant	gall of *Dryophanta divisa*	89
01503094	TIOXOLONE	C7H4O3S	168.17	antiseborrhoic	synthetic	88
00300038	JUGLONE	C10H6O3	174.15	antineoplastic. antifungal	leaves and nuts of *Juglans spp.. Carya spp and Pterocarya spp.*	88
01505007	2.3-DIMERCAPTOSUCCINIC ACID	C4H6O4S2	182.21	chelating agent. antihypertensive	synthetic; DMSA	88
00201513	EPIGALLOCATECHIN 3.5-DIGALLATE	C29H22O15	610.48		tea pigment	88
00210239	EPIGALLOCATECHIN-3-MONOGALLATE	C22H18O11	458.38		tea pigment	87
00210238	EPICATECHIN MONOGALLATE	C22H18O10	442.38		tea pigment	86
00200488	NORSTICTIC ACID PENTAACETATE	C28H24O15	600.49		derivative of norstictic acid (00240916)	86
01500287	ETHACRYNIC ACID	C13H12Cl2O4	303.14	diuretic	synthetic	85
00310035	SANGUINARINE SULFATE	C20H15NO8S	429.40	antineoplastic. antiplaque agent	*Sanguinaria canadensis*	84
00200033	LEOIDIN	C18H14Cl2O7	413.21		lichen metabolite	83
01501150	SULPIRIDE	C15H23N3O4S	341.43	dopamine receptor antagonist. antipsychotic	synthetic	83
01504019	GOSSYPOL	C30H30O8	518.56	antispermatogenic. antineoplastic. antiHIV	*Gossypium spp*	83
01505177	RUBESCENSIN A	C20H28O6	364.44	antibacterial. antineoplastic.	*Isodon trichocarpus. I*	82

				insect growth inhibitor	*japonicus. Rabdosia spp*	
00200424	2.3.4-TRIHYDROXY-4'-ETHOXYBENZOPHENONE	C15H14O5	274.27		semisynthetic	79
10101011	BIXIN	C25H30O4	394.51		*Bixa orellana* seeds	79
01500186	CHLORTETRACYCLINE HYDROCHLORIDE	C22H24Cl2N2O8	515.35	antibacterial. antiamebic. Ca chelator. hepatotoxic; inhibits protein synthesis	*Streptomyces aureofaciens*	77
02300205	LEVODOPA	C9H11NO4	197.19	antiparkinsonian	*Vicia faba seedlings. Sarothamnus spp. & other palnts*	77
01500603	TYROTHRICIN	C66H85N11O15	1272.47	topical antibacterial (topical)	*Bacillus aneurinolyticus; mixture of gramicidins & tyrocidines (tyrocidine A shown)*	76
01500719	7.2'-DIHYDROXYFLAVONE	C15H10O4	254.24	antihaemorrhagic	*Primula spp*	76
00201539	GARCINOLIC ACID	C38H46O9	646.78		*Garcinia spp.;* also hydrolysis product of gambogic acid	75
01500436	NOREPINEPHRINE	C8H11NO3	169.18	adrenergic agonist. antihypotensive	synthetic	74
01503806	HOMIDIUM BROMIDE	C21H20BrN3	394.31	antiprotozoal. intercalcate with DNA	synthetic	72
01500414	MINOCYCLINE HYDROCHLORIDE	C23H28ClN3O7	493.94	antibacterial	semisynthetic	71
01505782	CEFTAZIDIME	C22H22N6O7S2	546.58	antibacterial	semisynthetic	70
01505751	ERYTHROSINE SODIUM	C20H6I4Na2O5	879.86	color additive	synthetic	69
00200463	BRAZILEIN	C16H12O5	284.27		*Caesalpinia spp*	66
01505746	7-AMINOCEPHALOSPORANIC ACID	C10H12N2O5S	272.28		derivative of *Cephalosporin C*	64
01300037	SODIUM NITROPRUSSIDE	C5FeN6Na2O	261.92	antihypertensive	synthetic	64
01500644	PHENYLMERCURIC ACETATE	C8H8HgO2	336.74	antifungal	synthetic	62
01501015	FLUFENAMIC ACID	C14H10F3NO2	281.23	antiinflammatory. analgesic	synthetic	62
01500122	AMPHOTERICIN B	C47H73NO17	924.10	antifungal	*Streptomycetes nodosus*	61
00300554	PHLORETIN	C15H14O5	274.27		*Prunus spp*	60
01505141	2'.4-DIHYDROXYCHALCONE	C15H12O3	240.26		*Adhatoda vasica* (as glucoside)	58
01300030	OMEGA-3-ACID ESTERS (EPA shown)	C22H34O2	330.51	hypolipidemic	semisynthetic; K-85	57
01505127	GOSSYPIN	C21H20O13	480.38		*Gossypium spp. Hibiscus spp*	57
01500276	ERGOCALCIFEROL	C28H44O	396.66	antirachitic vitamin; LD50	irradiation of ergosterol	56

				(rat) 56 mg/kg po		
01505036	TANSHINONE IIA SULFONATE SODIUM	C19H17NaO6S	396.39	free radical scavenger	semisynthetic	55
00700024	HAEMATOPORPHYRIN	C34H38N4O6	598.70	antidepressant. antineoplastic	*Chlorella vulgaris;* derived blood	55
00201092	4-METHOXYDALBERGIONE	C16H14O3	254.28		*Dalbergia retusa and D. nigra*	52
01505824	TANSHINONE IIA	C19H18O3	294.35	antineoplastic. bone resorption inhibitor. antiproliferative. apoptosis inducer	*Salvia miltiorrhiza*	50
00200413	2.6-DIMETHOXYQUINONE	C8H8O4	168.15	antibacterial. induces dermatitis. mutagen	*Picrasmus & Ailanthus spp*	50
01500260	PYRITHIONE ZINC	C10H10N2O2S2 Zn	319.70	antibacterial. antifungal. antiseborrheic	synthetic	49
01500671	DIHYDROFOLIC ACID	C19H21N7O6	443.42	antidote to methotrexate toxicity	dihydrofolate reductase product	49
01500492	POLYMYXIN B SULFATE	C56H100N16O1 7S	1301.54	antibacterial	*Bacillus polymyxa*	48
01505300	PURPURIN	C14H8O5	256.21	xanthin oxidase inhibitor. irritant	*Rubia and Gallium spp*	45
01500856	CHOL-11-ENIC ACID	C24H36O3	372.55		semisynthetic	45
01503427	ALOIN	C21H22O10	434.40	cathartic. laxative	aloe	45
01505190	L(+/-)-ALLIIN	C6H11NO3S	177.22	antibacterial. antioxidant	*Allium species*	44
01505876	CHICAGO SKY BLUE	C34H24N6Na4O 16S4	992.81	inhibitor of aminoacid uptake	synthetic	41
01500446	NYSTATIN	C47H75NO17	926.11	antifungal. binds to membrane sterols	*Streptomyces noursei*	41
01505847	LACCAIC ACID A	C26H19NO12	537.44		*Coccus laccae* exudate; lac resin	40
00200690	2-BENZOYL-5-METHOXYBENZOQUINONE	C14H10O4	242.23		synthetic dalbergione analog	38
00200407	4'-HYDROXYCHALCONE	C15H12O2	224.26		*Shorea robusta aglycone*	38
01502109	ZIDOVUDINE [AZT]	C10H13N5O4	267.24	RT transferase inhibitor. antiviral	synthetic	36
01505356	DECOQUINATE	C24H35NO5	417.55	coccidiostat	synthetic; M&B-15497. HC-1528	36
01505257	ICARIIN	C33H40O15	676.67	hepatoprotective	*Epimedium spp*	36
01504118	DIFFRACTAIC ACID	C20H22O7	374.39		*Usnea spp*	35
01503926	LANSOPRAZOLE	C16H14F3N3O2 S	369.36	antiulcer	synthetic	33
00100146	7-DESACETOXY-6.7-DEHYDROGEDUNIN	C26H30O5	422.52		derivative of gedunin	33

01502247	FISETIN	C15H10O6	286.24	antioxidant	*Rhus and Acacia spp.*	33
01500836	CEPHALOSPORIN C SODIUM	C16H20N3NaO8S	437.40	antibacterial	*Cephalosprium acremonium*	32
01503070	MEFLOQUINE	C17H17ClF6N2O	414.78	antimalarial	synthetic	32
01500165	CEFOTAXIME SODIUM	C16H16N5NaO7S2	477.45	antibacterial	semisynthetic	32
01505158	2.3-DICHLORO-5.8-DIHYDROXYNAPTHOQUINONE	C10H4Cl2O4	259.04	apoptosis inducer. antiproliferative	synthetic	30
01505742	ASCORBYL PALMITATE	C22H38O7	414.54	antioxidant	semisynthetic	28
01505256	SILIBININ	C25H22O10	482.44	hepatoprotective agent. antioxidant	*Silybum marianum*	27
01505296	OXFENDAZOLE	C15H13N3O3S	315.35	anthelmintic	synthetic	27
01505332	AZAPERONE	C19H22FN3O	327.40	tranquilizer. neuroleptic. alpha adrenergic blocker	synthetic; R-1929	25
10100003	BIOCHANIN A	C16H12O5	284.27	phytoestrogen	widely distributed in *Leguminosae*	25
01505637	CEFPODOXIME PROXETIL	C21H27N5O9S2	557.60	antibacterial	semisynthetic; U-76252. CS-807	24
01500771	CEFACLOR	C15H14ClN3O4S	367.81	antibacterial	semisynthetic	24
01502074	4-NAPHTHALIMIDOBUTYRIC ACID	C16H13NO4	283.28	aldose reductase inhibitor	synthetic	23
01500420	NAFCILLIN SODIUM	C21H21N2NaO5S	436.46	antibacterial	semisynthetic	23
01505121	11a-ACETOXYPROGESTERONE	C23H32O4	372.50	metabolite of progesterone	semisynthetic	23
01500672	QUERCETIN	C15H10O7	302.24	capillary protectant. antioxidant. antineoplastic. anti-HIV; LD50(mouse) 159 mg/kg po	*Solanaceae. Rhamnaceae. Passifloraceae. Umbelliferae genera*	22
01505217	RHODOCLADONIC ACID	C15H10O8	318.24		*Cladonia spp*	22
01505679	TETRAMIZOLE HYDROCHLORIDE	C11H13ClN2S	240.75	anthelmintic	synthetic; R-8299. McN-JR-8299-11	22
00200110	ANTIAROL	C9H12O4	184.19		*Antiaris toxicaria*	21
01505467	ZINC UNDECYLENATE	C22H38O4Zn	431.91	antifungal	synthetic	21
01500196	CLOMIPHENE CITRATE	C32H36ClNO8	598.09	gonad stimulating principle	synthetic	21
01500556	SULINDAC	C20H17FO3S	356.41	antiinflammatory	synthetic	21
01505108	L-BUTHIONINE SULFOXIMINE	C8H18N2O3S	222.30	inhibitor of gamma-glutamylcysteine synthetase	synthetic	20
01500844	COENZYME B12	C72H104CoN18O17P	1583.61	vitamin	Vitamin B12; cyanocobalamin	20

01502045	PIROMIDIC ACID	C14H16N4O3	288.30	antibacterial	synthetic; PD-93	19
01504226	CYSTEAMINE HYDROCHLORIDE	C2H8ClNS	113.60	antiurolithic. depigmentation. radiation protectant	cysteamine; CL-9148	19
01503065	ALTRETAMINE	C9H18N6	210.28	antineoplastic	synthetic	19
01505132	2'.4'-DIHYDROXYCHALCONE	C15H12O3	240.26	anthelmintic. antiulcer	*Flemingia chappar. Ceratiola ericoides. Acacia neovernicosa. Flourensia spp*	19
01503106	BEPRIDIL HYDROCHLORIDE	C24H35ClN2O	403.01	antiarrhythmic	synthetic; CERM-1978	18
01500529	RIFAMPIN	C43H58N4O12	822.96	antibacterial (tuberculostatic)	semisynthetic; L-5103. Ba-41166/E. NSC-113926	18
01500514	DEXPROPRANOLOL HYDROCHLORIDE	C16H22ClNO2	295.81	antihypertensive. antianginal. antiarrhythmic	synthetic	18
01500802	ALLOXAN	C4H2N2O4	142.07	specific cytotoxin (pacreatic islet betacell)	synthetic	18
01505208	CEFDINIR	C14H13N5O5S2	395.41	antibacterial	semisynthetic; CL-983. FK-482	17
01502196	ETHOSUXIMIDE	C7H11NO2	141.17	anticonvulsant	synthetic	17
00200425	2.3-DIHYDROXY-4-METHOXY-4'-ETHOXYBENZOPHENONE	C16H16O5	288.30		derivative	16
01505112	TILMICOSIN	C46H80N2O13	869.15	antibacterial	semisynthetic	16
01506047	TIRATRICOL	C14H9I3O4	621.93	thyroid agent	synthetic	16
01503923	IOPANIC ACID	C11H12I3NO2	570.93	radioopaque agent	synthetic	16
01505812	CRYPTOTANSHINONE	C19H20O3	296.36	inhibits angiogenesis	*Salvia miltiorrhiza. Rosmarinus officinalis*	16
01502038	CEFAMANDOLE SODIUM	C18H17N6NaO5S2	484.49	antibacterial	semisynthetic	15
01504148	MEPIVACAINE HYDROCHLORIDE	C15H23ClN2O	282.81	anesthetic (local)	synthetic	15
01505660	PHENTERMINE	C10H15N	149.23	anorexic	synthetic	15
01505212	PERINDOPRIL ERBUMINE	C23H43N3O5	441.61	antihypertensive. ACE inhibitor	synthetic; S9490-3. McN-A2833-109	15
01505333	TRANILAST	C18H17NO5	327.33	antiallergic. mast cell degranulation inhibitor. angiogenesis blocker	synthetic; MK-341	15
01503604	RETINYL PALMITATE	C36H60O2	524.87	provitamin. antixerophthalamic	semisynthetic; Vitamin A palmitate	15
01500274	ADRENALINE BITARTRATE	C13H19NO9	333.29	adrenergic agonist. bronchodilator.	*Portulaca grandiflora*	15

				antiglaucoma agent		
01503324	THIOTEPA	C6H12N3PS	189.22	antineoplastic. alkylating agent	synthetic	14
00102058	OLEANOLIC ACID ACETATE	C32H50O4	498.75		birch bark	14
01505134	MANGIFERIN	C19H18O11	422.34	MAO inhibitor. immunostimulant	*Mangifera indica. Iris & Salacia spp. Aphloia. Athyrium. Anemarrhena. Belamcanda chinensis. Hedysarum ussuriense*	14
01504065	MYRICETIN	C15H10O8	318.24	antiHIV. topoisomerase II inhibitor	*Myrica spp*	14
01505267	OXAPROZIN	C18H15NO3	293.32	antiinflammatory	synthetic; WY-21743	13
01505675	TRIENTINE HYDROCHLORIDE	C6H20Cl2N4	219.15	chelating agent	synthetic; MK-0681	13
00307059	CEDROL	C15H26O	222.37	acaricide	Common constitutent in the Family *Cupressaceae*	13
01503202	CHLOROXINE	C9H5Cl2NO	214.05	chelating agent	synthetic	13
00211475	4'-METHOXYCHALCONE	C16H14O2	238.28		*Citrus limon*	12
01505330	OXICONAZOLE NITRATE	C18H14Cl4N4O4	492.14	antifungal	synthetic	12
01505560	NATAMYCIN	C33H47NO13	665.74	antibacterial	*Steptomyces natalensis; CL-12625. A-5283*	12
01500586	TRIAMCINOLONE	C21H27FO6	394.44	glucocorticoid	semisynthetic	11
00240429	3.4-DIDESMETHYL-5-DESHYDROXY-3'-ETHOXYSCLEROIN	C15H14O5	274.27		synthetic	11
00100049	KHAYANTHONE	C32H42O9	570.68		*Khaya species*	11
01505012	EXEMESTANE	C20H24O2	296.41	antineoplastic. aromatase inhibitor	synthetic; PNU-155971. FCE-24304	11
01500315	GENTIAN VIOLET	C25H30ClN3	407.99	antibacterial. anthelmintic	synthetic	11
01503710	CLOPIDOGREL SULFATE	C16H18ClNO6S2	419.90	platelet aggregation inhibitor	synthetic	11
01503004	BENZOYL PEROXIDE	C14H10O4	242.23	keratolytic	synthetic; NSC-675	11
01501176	ERYTHROMYCIN ESTOLATE	C52H97NO18S	1056.49	antibacterial	*Streptomyces erythreus*	10
01505174	GARLICIN	C6H10S2	146.27	antineoplastic. antibacterial. apoptosis inducer. insecticide	*Allium spp. Descurainia sophia*	10
01504259	IRBESARTAN	C25H28N6O	428.54	angiotensin 2 receptor antagonist	synthetic	10
01503110	CARMUSTINE	C5H9Cl2N3O2	214.05	antineoplastic. alkylating agent	synthetic	10
00211224	alpha-TOXICAROL	C23H22O7	410.42		*Derris spp*	10

01505034	BACCATIN III	C31H38O11	586.64		*Taxus baccata*	9
01503251	METARAMINOL BITARTRATE	C13H19NO8	317.29	antihypotensive	synthetic	9
01502111	AZACITIDINE	C8H12N4O5	244.20	antineoplastic. pyrimidine antimetabolite	synthetic; U-18496; NSC-102816	9
00205071	HAEMATOMMIC ACID. ETHYL ESTER	C11H12O5	224.21		various lichens. e.g. *Evernia spp. Parmelia spp*	8
00300610	ACETOSYRINGONE	C10H12O4	196.20	insect attractant. plant hormone	several commercial woods	8
01500325	HALOPERIDOL	C21H23ClFNO2	375.87	antidyskinetic. antipsychotic	synthetic	8
01505811	LEVALBUTEROL HYDROCHLORIDE	C13H22ClNO3	275.77	bronchodilator. tocolytic	synthetic	8
00240927	CAPERATIC ACID	C21H38O7	402.53	antibacterial (tuberculostatic)	lichens: *Parmelia. Mycoblastus. Nephromopsis spp*	8
01503603	ACYCLOVIR	C8H11N5O3	225.20	antiviral	synthetic	8
01500127	ANTHRALIN	C14H10O3	226.23	antipsoriatic	synthetic	8
01501157	SUXIBUZONE	C24H26N2O6	438.48	analgesic. antipyretic. antiinflammatory	synthetic	8
00300563	TRICHLORMETHINE	C6H13Cl4N	240.98	antineoplastic. cytotoxic	synthetic	7
01502245	ELLAGIC ACID	C14H6O8	302.19	hemostatic. antineoplastic. antimutagenic	widely distributed in higher plants	7
01505022	PROPOFOL	C12H18O	178.27	anesthetic	synthetic; ICI-35868	7
01504181	PRISTIMERIN	C30H40O4	464.65	antineoplastic. antiinflammatory	*Celastrus and Maytenus spp*	7
01505265	TELITHROMYCIN	C43H65N5O10	812.02	antibacterial	semisynthetic; HMR-3647	7
01505693	OMEPRAZOLE	C17H19N3O3S	345.42	gastric acid depressant	synthetic; H-168/68	6
01500377	MECLOFENAMATE SODIUM	C14H10Cl2NNaO2	318.13	antiinflammatory. antipyretic	synthetic	6
01504508	CINCHOPHEN	C16H11NO2	249.27	analgesic. antipyretic. antiinflammatory	synthetic	6
01504911	FLUVASTATIN	C24H26FNO4	411.47	antihyperlipidemic. HMGCoA reductase inhibitor	synthetic; XU-62-320	6
01500702	ACETYLTRYPTOPHAN	C13H14N2O3	246.26	antidepressant	synthetic	6
01501188	EBSELEN	C13H9NOSe	274.18	antioxidant. lipoxygenase inhibitor. inhibits oxidation of LDL	synthetic	6
01500817	CARMINIC ACID	C22H20O14	508.39		*Dactylopius coccus (cochineal)*	6

01505982	OXANTEL PAMOATE	C36H32N2O7	604.66	anthelmintic	synthetic; CP-14445-16	6
00200774	IRIGENIN	C18H16O8	360.32		*Iris spp*	5
01500364	LEUCOVORIN CALCIUM	C20H21CaN7O7	511.51	antianemic. antidote to folic acid antagonists	synthetic	5
00100201	3-DEOXO-3beta-ACETOXYDEOXYDIHYDROGEDUNIN	C30H40O7	512.64		*Meliaceae spp*	5
01503102	BACAMPICILLIN HYDROCHLORIDE	C21H28ClN3O7S	501.99	antibacterial	semisynthetic	5
01503938	RIBAVIRIN	C8H12N4O5	244.20	antiviral	synthetic	5
01505684	ROLITETRACYCLINE	C27H33N3O8	527.57	antibacterial	semisynthetic; SQ-15659	5
01504206	BOVINOCIDIN (3-nitropropionic acid)	C3H5NO4	119.07	antineoplastic	*Aspergillus. Streptomyces* spp & other microorganisms	5
01500552	SULFASALAZINE	C18H14N4O5S	398.40	anticolitis and Crohn's disease	synthetic	5
01500570	THIABENDAZOLE	C10H7N3S	201.25	anthelmintic	synthetic	5
01500134	BACITRACIN	C66H103N17O16S	1422.71	antibacterial	*Bacillus licheniformis and B subtilis*	5
00201315	7.8-DIHYDROXYFLAVONE	C15H10O4	254.24	vascular protectant. antihaemorrhagic	*Godmania aesculifolia*	5
00212097	ONONETIN	C15H14O4	258.27		*Trifolium subterraneum*	5
01500245	DIFLUNISAL	C13H8F2O3	250.20	analgesic. antiinflammatory	synthetic	5
01505713	PREGNENOLONE SUCCINATE	C25H36O5	416.56	glucocortcoid. antiinflammatory	semisynthetic	4
01500815	BETULIN	C30H50O2	442.73		*Betula spp*	4
01500814	CANTHARIDIN	C10H12O4	196.20	irritant	active principle of cantharides and other insects	4
01503936	PERICIAZINE	C21H23N3OS	365.50	antipsychotic	synthetic	4
01504088	ETHYLNOREPINEPHRINE HYDROCHLORIDE	C10H16ClNO3	233.69	bronchodilator	synthetic	4
01505783	OXYPHENONIUM BROMIDE	C21H34BrNO3	428.41	anticholinergic. anticonvulsant	synthetic; BA-5473. C-5473	4
01502044	OFLOXACIN	C18H20FN3O4	361.37	antibacterial	synthetic	4
01500465	PENICILLIN G POTASSIUM	C16H17KN2O4S	372.49	antibacterial	*Penicillium spp*	4
01505461	SULFISOXAZOLE ACETYL	C13H15N3O4S	309.34	antibacterial	synthetic	4
00200446	METHYLXANTHOXYLIN	C11H14O4	210.23		*Acradenia franklinii. Eugenia jambolana*	4
01504173	FLUOXETINE	C17H19ClF3NO	345.79	antidepressant	synthetic	4
01500669	BENFLUOREX HYDROCHLORIDE	C19H21ClF3NO2	387.83	antihyperlipoproteinemic	synthetic	4
01500865	HARMALOL HYDROCHLORIDE	C12H13ClN2O	236.70	anthelmintic. narcotic agent	*Peganium harmala*	4

00330008	2.4-DINITROPHENOL	C6H4N2O5	184.10	uncouples oxidative phosphorylation	synthetic	4
01500408	METHYLTHIOURACIL	C5H6N2OS	142.18	antithyroid agent	synthetic	4
01505532	DILOXANIDE FUROATE	C14H11Cl2NO4	328.15	amoebicide	synthetic	4
01504008	SIROLIMUS	C51H79NO13	914.19	immunosuppressant. antineoplastic; rapamycin	*Streptomyces hygroscopicus*	4
01500239	DICUMAROL	C19H12O6	336.30	anticoagulant	synthetic	4
01500907	DEHYDROCHOLIC ACID	C24H34O5	402.53	choleretic	derivative of cholic acid	4
01504523	FTAXILIDE	C16H15NO3	269.30	antiulcer	synthetic; MP-12	4
01503903	ALBENDAZOLE	C12H15N3O2S	265.33	anthelmintic	synthetic	4.
01500343	HYDROXYPROGESTERONE CAPROATE	C27H40O4	428.61	progestogen	semisynthetic	3
00310050	QUININE ETHYL CARBONATE	C23H28N2O4	396.49	antimalarial	semisynthetic	3
01503421	RITANSERIN	C27H25F2N3OS	477.58	antiserotonin	synthetic	3
01504236	SIMVASTATIN	C25H38O5	418.57	antihyperlipidemic. HMGCoA reductase inhibitor	synthetic	3
01505214	RAMIPRIL	C23H32N2O5	416.52	antihypertensive. ACE inhibitor	synthetic; HOE-498	3
01505294	CARBADOX	C11H10N4O4	262.22	antibacterial	synthetic	3
01505973	CYPROHEPTADINE HYDROCHLORIDE	C21H22ClN	323.86	H1-antihistamine. antipruritic	synthetic	3
00300057	LARIXOL ACETATE	C22H36O3	348.53		*Larix europaea*	3
01503001	BENZOIC ACID	C7H6O2	122.12	antifungal	synthetic	3
01503081	AMIFOSTINE	C5H15N2O3PS	214.22	radioprotectant	synthetic	3
01501183	ESTRADIOL METHYL ETHER	C19H26O2	286.41	estrogen	semisynthetic	3
01500331	HISTAMINE DIHYDROCHLORIDE	C5H11Cl2N3	184.06	H receptor agonist. induces edema in mammalian tissues; gastric secretion stimulant	*Claviceps purpurea. many plants and animals*	3
01500218	DACARBAZINE	C6H10N6O	182.18	antineoplastic	synthetic	3
01503212	DOBUTAMINE HYDROCHLORIDE	C18H24ClNO3	337.84	cardiotonic	synthetic	3
00300117	ISOKOBUSONE	C14H22O2	222.33		*Cyperus rotundus. Sindora sumatrana*	3
01503974	DIMETHYL 4.4-o-PHENYLENE-BIS (3-THIOPHANATE)	C12H14N4O4S2	342.39	antifungal (systemic plant)	synthetic	3
01500131	ATROPINE SULFATE	C17H25NO7S	387.45	anticholinergic. mydriatic	*Atropa and Datura spp*	3
01505818	PANTOPRAZOLE	C16H15F2N3O4S	383.37	proton pump inhibitor. gastric acid release inhibitor. antiulcer	synthetic; SK&F-96022. BY-1023	3

01503914	BUTACAINE	C18H30N2O2	306.45	anesthetic (local)	synthetic	3
01505444	METHYLENE BLUE	C16H20ClN3OS	337.87	antimethemoglobinemic. cyanide antidote	synthetic	3
01505410	BENURESTAT	C9H9ClN2O3	228.63	urease inhibitor	synthetic; EU-2826	3
01502238	DEOXYADENOSINE	C10H13N5O3	251.24		synthetic	3
01505249	APRAMYCIN	C21H41N5O11	539.58	antibacterial; LD50(iv) 280mg/kg(mouse)	*Streptomyces tenebrarius. Saccharomyces porispora hiltus*	3
01505986	NIALAMIDE	C16H18N4O2	298.34	MAO inhibitor	synthetic	3
01500624	CARNITINE (dl) HYDROCHLORIDE	C7H16ClNO3	197.66	antihyperlipoproteinemic. gastric/ pancreatic secretion stimulant	striated muscle. liver; also in Pisum sativum	3
01504036	3-DESHYDROXYSAPPANOL TRIMETHYL ETHER	C19H22O5	330.38		derivative	3
01500709	CHRYSIN	C15H10O4	254.24	diuretic	*Ulnus sieboldiana. Flourensia resinosa. Oroxylum indicum. Pinus and Scutellaria spp.*	3
00200457	ANHYDROBRAZILIC ACID	C12H10O5	234.21		derivative	3
01503228	PAROMOMYCIN SULFATE	C23H47N5O18S	713.71	antibacterial. antiamebic	*Streptomyces rimosis paramomycinus*	3
01502033	CEFUROXIME SODIUM	C16H15N4NaO8S	446.37	antibacterial	*semisynthetic*	3
01505953	SUCRALOSE	C12H19Cl3O8	397.63	sweetener	semisynthetic	3
01500303	FLUOCINONIDE	C26H32F2O7	494.53	antiinflammatory. glucocorticoid	semisynthetic	3
01505757	MELIBIOSE	C12H22O11	342.30		yeast	3
00240437	PERSITOL HEPTAACETATE	C21H30O14	506.46		derivative of perseitol	3
00200004	12a-HYDROXY-9-DEMETHYLMUNDUSERONE-8-CARBOXYLIC ACID	C19H16O9	388.33		derivative	3
01502031	CEFOXITIN SODIUM	C16H16N3NaO7S2	449.43	antibacterial	semisynthetic	3
00300565	METERGOLINE	C25H29N3O2	403.52	analgesic. antipyretic	synthetic; FI-6337. MCE	3
01500648	AZELAIC ACID	C9H16O4	188.22	antiacne. antiproliferative agent	rancid fats and *Lycopodium spp*	3
01503606	CYPERMETHRIN	C22H19Cl2NO3	416.30	insecticide	synthetic	3
00501000	d.l-threo-3-HYDROXYASPARTIC ACID	C4H7NO5	149.10	L-aspartate beta-carboxylase inhibitor	*Streptomyces spp. Arthrinium phaeospermum. Dactylosporangium aurantiacum*	3
00100655	DIHYDROGEDUNIC ACID. METHYL ESTER	C26H36O8	476.57		derivative	3

01502237	HARMOL HYDROCHLORIDE	C12H11ClN2O	234.68	MAO inhibitor	common plant alkaloid	3
01500478	PHENIRAMINE MALEATE	C20H24N2O4	356.42	antihistaminic	synthetic	3
01506031	PIRIBEDIL HYDROCHLORIDE	C16H18N4O2	298.34	dopamine agonist	synthetic; EU-4200. ET-495	3
01504506	TOSYLCHLORAMIDE SODIUM	C7H7ClNNaO2S	227.64	antiseptic. disinfestant. antiproliferative	synthetic	3
01500509	PROMAZINE HYDROCHLORIDE	C17H21ClN2S	320.88	antipsychotic	synthetic	3
01500522	QUINACRINE HYDROCHLORIDE	C23H32Cl3N3O	472.88	anthelmintic. antimalarial. intercalating agent	synthetic	3
01500383	MEPENZOLATE BROMIDE	C21H26BrNO3	420.35	anticholinergic	synthetic	3
01503640	PARTHENOLIDE	C15H20O3	248.32	5HT antagonist. antineoplastic. smooth muscle relaxant	*Chrysanthemum parthenium. Michelia champaca*	3
01502113	AZASERINE	C5H7N3O4	173.12	antineoplastic. amino acid antagonist	*Streptomyces fragilis*	3
01503209	DANTROLENE SODIUM	C14H9N4NaO5	336.24	muscle relaxant (skeletal)	synthetic	3
01500390	METAPROTERENOL	C11H17NO3	211.26	bronchodilator	synthetic	3
01500284	ESTRADIOL VALERATE	C23H32O3	356.50	estrogen	semisynthetic	3
01500632	HEXESTROL	C18H22O2	270.37	estrogen. antineoplastic (hormonal)	synthetic; NSC-9894	3
01504027	CYTISINE	C11H14N2O	190.24	antiinflammatory. respiratory stimulant	*Thermopsis lanceolata*	3
01502034	METAMPICILLIN SODIUM	C17H18N3NaO4S	383.40	antibacterial	semisynthetic	2
01505584	ERGOTAMINE TARTRATE	C38H43N5O10	729.79	analgesic. antimigraine	*Claviceps purpurea*	2
01501174	TODRALAZINE HYDROCHLORIDE	C11H13ClN4O2	268.70	antihypertensive. peripheral vasodilator	synthetic; CEPH. BT-621	2
01503121	TRAZODONE HYDROCHLORIDE	C19H23Cl2N5O	408.33	antidepressant	synthetic	2
00100114	3alpha-HYDROXY-3-DEOXYANGOLENSIC ACID METHYL ESTER	C27H36O7	472.58		*Meliaceae spp*	2
01600480	TETRAHYDROSAPPANONE A TRIMETHYL ETHER	C19H22O5	330.38		derivative *Caesalpinia sappan*	2
01500752	QUERCITRIN	C21H20O11	448.38	antihemmorrhagic	widespread in plants	2
00310023	HYPOXANTHINE	C5H4N4O	136.11		widely distributed in the plant and animal kingdom	2
01500813	BUDESONIDE	C25H34O6	430.54	antiinflammatory	semisynthetic	2
01505349	4-O-METHYLPHLORACETOPHENONE	C9H10O4	182.17	antifungal	*Prunus domesticus*	2
01503818	BUPIVACAINE HYDROCHLORIDE	C18H29ClN2O	324.89	anesthetic (local)	synthetic	2

01505305	PEFLOXACINE MESYLATE	C18H24FN3O6S	429.47	antibacterial. antiproliferative	synthetic	2
01502094	ROSMARINIC ACID	C18H16O8	360.32	antiinflammatory. antithrombotic. antiplatelet. cytostatic. antiviral	*Rosmarinus officinalis. Melissa officinalis. Momordica balsamina. Mentha piperita. Salvia officinalis. Teucrium scorodonia. Sanicula europaea. Coleus blumei. Thymus spp*	2
01500359	ISOXSUPRINE HYDROCHLORIDE	C18H24ClNO3	337.84	vasodilator	synthetic	2
01504204	CANTHAXANTHIN (euglenanone)	C40H52O2	564.85		*Cantharellus cinnabarinus*	2
00200763	IRIGENIN. 7-BENZYL ETHER	C25H22O8	450.44		derivative of irigenin from Iris spp.	2
00300539	ARBUTIN	C12H16O7	272.25		*Berginia crassifolia; also in Pyrus and Vaccinium spp.*	2
01504079	TOMATINE	C47H79NO21	994.14	antifungal. antibacterial. antiinflammatory agent	*Solanum spp.*	2
00300118	3.7-EPOXYCARYOPHYLLAN-6-OL	C15H26O2	238.37		derivative *Lippia spp*	2
00100447	DEACETOXY(7)-7-OXOKHIVORINIC ACID	C27H36O10	520.58		derivative	2
01501008	FENBUFEN	C16H14O3	254.28	antiinflammatory	synthetic	2
01505153	2'.3-DIHYDROXY-4.4'.6'-TRIMETHOXYCHALCONE	C18H18O6	330.34		*Merrillia caloxylon*	2
01500549	SULFAMETHIZOLE	C9H10N4O2S2	270.33	antibacterial	synthetic	2
00100355	1.3-DIDEACETYL-7-DEACETOXY-7-OXOKHIVORIN	C26H34O7	458.55		*Meliaceae spp*	2
01504403	DONEPEZIL HYDROCHLORIDE	C24H30ClNO3	415.96	acetylcholinesterase inhibitor (reversible). cognitive enhancer	synthetic; E-2020. BNAG	2
00201605	BISANHYDRORUTILANTINONE	C22H16O7	392.36	antibacterial	*Streptomyces spp; also rutilantinone*	2
01505465	TRICLOSAN	C12H7Cl3O2	289.54	antiinfective	synthetic; CH-3565	2
01505714	DARIFENACIN HYDROBROMIDE	C28H31BrN2O2	507.47	M3 muscarinic antagonist. bladder suppressant	synthetic; UK-88525-04	2
00210206	EPICATECHIN	C15H14O6	290.27	antioxidant	tea and cocoa constituent	2
01500394	METHENAMINE	C6H12N4	140.18	antibacterial (urinary)	synthetic	2

00201580	POMIFERIN	C25H24O6	420.46	antioxidant	*Maclura pomifera*	2
01505152	2'.4'-DIHYDROXY-4-METHOXYCHALCONE	C16H14O4	270.28		*Bauhinia manca*	2
01503298	DIPYRONE	C13H16N3NaO4S	333.34	analgesic. antipyretic	synthetic	2
01504156	ANABASAMINE HYDROCHLORIDE	C16H20ClN3	289.81		*Anabasis aphylla*	2
01500906	LITHOCHOLIC ACID	C24H40O3	376.58	LD50(mouse) 3900 mg/kg po	mammalian bile and gallstones. faecal matter	2
01500741	6.4'-DIMETHOXYFLAVONE	C17H14O4	282.29		derivative *Cassia spectabilis*	2
01502046	BEZAFIBRATE	C19H20ClNO4	361.82	antihyperlipidemic	synthetic	2
01500822	BRUCINE	C23H26N2O4	394.47	central stimulant	*Strychnos nux-vomica*	2
01503373	OXIBENDAZOLE	C12H15N3O3	249.27	anthelmintic	synthetic	2
00200427	PISCIDIC ACID	C11H12O7	256.21		*Piscidia piscipula*	2
01503203	CHLORPROTHIXENE HYDROCHLORIDE	C18H19Cl2NS	352.32	antipsychotic	synthetic	2
00210296	GENISTEIN	C15H10O5	270.24		widely distributed in Leguminoseae	2
01503712	LORATADINE	C22H23ClN2O2	382.89	H1 antihistamine	synthetic	2
01504044	RESVERATROL 4'-METHYL ETHER	C15H14O3	242.27		derivative	2
01500995	FLUTAMIDE	C11H11F3N2O3	276.21	antiandrogen	synthetic	2
01503918	CLOBETASOL PROPIONATE	C25H32ClFO5	466.98	glucocorticoid. antiinflammatory	synthetic	2
00100424	XYLOCARPUS A	C31H38O11	586.64		*Meliaceae spp*	2
01505360	CEFDITORIN PIVOXIL	C25H28N6O7S3	620.72	antibacterial	semisynthetic	2
01500256	DIPHENHYDRAMINE HYDROCHLORIDE	C17H22ClNO	291.82	antihistaminic	synthetic	2
01506039	ETICLOPRIDE HYDROCHLORIDE	C17H26Cl2N2O3	377.31	dopamine D2 blocker	synthetic	2
00310004	ANABASINE HYDROCHLORIDE	C10H15ClN2	198.69	insecticide	*Anabasis aphylla & Nicotiana spp*	2
01505599	ETOMIDATE	C14H16N2O2	244.29	sedative	synthetic	2
01502016	TRETINON	C20H28O2	300.44	keratolytic	semisynthetic	2
01505581	TIOCONAZOLE	C16H13Cl3N2OS	387.71	antifungal	synthetic; UK-20349	2
01505978	FLORFENICOL	C12H14Cl2FNO4S	358.21	antibacterial	synthetic; SCH-25298	2
01506011	6-METHOXYHARMALAN	C13H14N2O	214.26	convulsant. halucinogen	*Peganum spp*	2
01501185	ECONAZOLE NITRATE	C18H16Cl3N3O4	444.70	antifungal	SQ-13050; synthetic	2
01500729	4-METHYLESCULETIN	C10H8O4	192.17		analog of esculetin	2
00310299	THYMOQUINONE	C10H12O2	164.20		*Callitris. Monarda spp. Juniperus drus. Nigella sativa*	2

01506010	METHIONINE SULFOXIMINE (L)	C5H12N2O3S	180.22	glutamine synthetase inhibitor. ornithine decarboxylase enhancer	synthetic	2
00307050	DEHYDROABIETAMIDE	C20H29NO	299.46		derivative	2
01504137	ANCITABINE HYDROCHLORIDE	C9H12ClN3O4	261.66	antineoplastic	synthetic	2
01505230	CILOSTAZOL	C20H27N5O2	369.47	phosphodiesterase inhibitor	synthetic	2
01500760	HECOGENIN	C27H42O4	430.63	antiinflammatory	*Agave and Yucca spp*	2
00100595	COTARNINE CHLORIDE	C12H14ClNO3	255.70	vasoconstrictor	*Papaver pseudo-orientale*	2
01500259	DIPYRIDAMOLE	C24H40N8O4	504.63	coronary vasodilator	synthetic	2
01503383	PEMPIDINE TARTRATE	C14H27NO6	305.37	ganglionic blocker. antihypertensive	synthetic	2
01400010	IPRIFLAVONE	C18H16O3	280.32	anabolic	synthetic	2
01300019	OXIGLUTATIONE DISODIUM SALT	C20H30N6Na2O12S2	656.60	antioxidant	synthetic	2
00200744	ERGOSTEROL ACETATE	C32H50O2	466.75		derivative; mp 179-181 C	2
01505944	RISEDRONATE SODIUM HYDRATE	C7H16NNaO10P2	359.14	calcium regulator	synthetic; NE-58095	2
01500299	FLUDROCORTISONE ACETATE	C23H31FO6	422.49	mineralocorticoid	semisynthetic	2
01503278	MITOXANTHRONE HYDROCHLORIDE	C22H30Cl2N4O6	517.41	antineoplastic	semisynthetic	2
01505807	AVOCADENOFURAN	C17H28O	248.41		*Persea spp*	2
01500639	OCTOPAMINE HYDROCHLORIDE	C8H12ClNO2	189.64	adrenergic agonist	salivary glands of *Octopus vulgaris*; also *Capsicum frutescens & Cyperus spp*; ND-50	2
01500990	ENOXOLONE	C30H46O4	470.69	antitussive. antiinflammatory. antibacterial	derivative	2
00100497	3beta-ACETOXYDEOXODIHYDROGEDUNIN	C30H40O8	528.64		Meliaceae spp	2
01504075	RHOIFOLIN	C27H30O14	578.53		Chorisia. Citrus and Rhus spp	2
01506079	MOXISYLYTE HYDROCHORIDE	C16H25NO3	279.38	alpha-adrenergic blocker	synthetic	2
01500547	SULFAMERAZINE	C11H12N4O2S	264.30	antibacterial	synthetic	2
01503206	CLOFOCTOL	C21H26Cl2O	365.34	antibacterial	synthetic	2
01505319	CLOPIDOL	C7H7Cl2NO	192.04	coccidiostat. antiplatelet	synthetic	2
01505175	ASIATIC ACID	C30H48O5	488.71	wound healing. experimental carcinogen	Dipterocarpus pilosus. Dryobalanops aromatica	2

00200258	2'.4'-DIHYDROXYCHALCONE 4'-GLUCOSIDE	C21H22O8	402.40	anthelmintic & antiulcerocenic	aglycone Flamingia chappar. Acacia neovernicosa	2
01500363	LACTULOSE	C12H22O11	342.30	laxative	synthetic	2
01505770	FURALTADONE	C13H16N4O6	324.29	antibacterial	synthetic; NF-260	2
00201136	SAPPANONE A DIMETHYL ETHER	C18H16O5	312.32		Caesalpinia sappan	2
01505324	BISSALICYL FUMARATE	C18H12O8	356.29	crosslinking agent (hemoglobin)	synthetic	2
01503006	BENZYL ISOTHIOCYANATE	C8H7NS	149.21	antineoplastic. antibacterial. antifungal	Tropaeolum majus. Lepidium sativum and other Cruciferae	2
00100688	DIGOXIGENIN	C23H34O5	390.52		aglycon of digitoxin. thevetin. cerberin. echujin. evomonoside; mp 217-218	2
01500241	DIENESTROL	C18H18O2	266.34	estrogen	synthetic	2
00310010	HELENINE	C15H20O2	232.32	anthelmintic. antibacterial. antineoplastic	Inula spp	2
00300531	MELEZITOSE	C18H32O16	504.44		honey & plant exudates	2
01500716	ALANYL-dl-LEUCINE	C9H18N2O3	202.25		synthetic	2
01503083	AMIPRILOSE	C14H28ClNO6	341.83	immunomodulator. antiinflammatory	semisynthetic	2
01505977	ISOETHARINE MESYLATE	C14H25NO6S	335.42	bronchodilator	synthetic	2
01504120	CARNOSIC ACID	C20H28O4	332.44		Salvia spp. Rosmarinus officinalis	2
01500837	CHENODIOL	C24H40O4	392.58	anticholithogenic. antilipemic agent	human bile	2
01500804	ALTHIAZIDE	C11H14ClN3O4S3	383.89	diuretic	synthetic	2
01500263	DOPAMINE HYDROCHLORIDE	C8H12ClNO2	189.64	cardiotonic. antihypotensive	synthetic	2
01505355	LUFENURON	C17H8Cl2F8N2O3	511.15	molt inhibitor. chitin synthesis inhibitor; insecticide	synthetic; CGA-184699	2
01504002	BAICALEIN	C15H10O5	270.24	antiviral (HIV)	*Scutellaria baicalensis*	2
01500223	DAUNORUBICIN	C27H29NO10	527.53	antineoplastic	*Streptomyces peucetius; FI-6339. NDC-0082-4155. RP-13057*	2
01505222	DERACOXIB	C17H14F3N3O3S	397.37	antiinflammatory. antiarthritic. COX-2 inhibitor	synthetic	2

00100305	3beta-HYDROXYISOALLOSPIROST-9(11)-ENE	C27H42O3	414.63		semisynthetic	2
01503602	SALINOMYCIN. SODIUM	C42H69NaO11	773.00	antibacterial	*Streptomyces albus*	2
01504094	TENIPOSIDE	C32H32O13S	656.66	antineoplastic	semisynthetic	2
00100008	CARAPIN	C27H32O7	468.55		*Carapa and Cedrela species; mp 180-185*	2
02300161	AMOXAPINE	C17H16ClN3O	313.78	antidepressant. inhibits norepinephrine uptake	synthetic	2
00300537	XANTHOPTERIN	C6H5N5O2	179.13	cell proliferation inhibitor	human urine. butterfly wing pigment	2
01505764	SPERMINE	C10H26N4	202.34	immune modulator	all animal tissue. fungi	2
00100047	DEACETOXY-7-OXOGEDUNIN	C26H30O6	438.52		*Meliaceae spp*	2
01502095	alpha-CYANO-3-HYDROXYCINNAMIC ACID	C10H7NO3	189.17	inhibit mitochondrial pyruvate transport	synthetic	2
01500597	TRIPELENNAMINE CITRATE	C22H29N3O7	447.49	antihistaminic	synthetic	2
01501128	CAPSAICIN	C18H27NO3	305.42	analgesic (topical). depletes Substance P. neurotoxic	*Capsicum spp*	2
01505368	VALACYCLOVIR HYDROCHLORIDE	C13H21ClN6O4	360.80	antiviral	synthetic; BW-256-U-87	2
00100222	3-DEOXO-3beta-HYDROXYMEXICANOLIDE 16-ENOL ETHER	C28H36O7	484.59		derivative	2
01500870	MYOSMINE	C9H10N2	146.19	mitogen	tobacco. nuts	2
01502107	CISPLATIN	H6Cl2N2Pt	300.05	antineoplastic. convulsant	synthetic	2
01503990	CONESSINE	C24H40N2	356.59		*Holarrhena spp*	2
01500557	TAMOXIFEN CITRATE	C32H37NO8	563.65	estrogen antagonist. antineoplastic	synthetic	2
01504175	CEFUROXIME AXETIL	C20H22N4O10S	510.48	antibacterial	synthetic	2
01503297	HEXAMETHONIUM BROMIDE	C12H30Br2N2	362.19	antihypertensive. ganglionic blocker	synthetic	2
01500876	CORYNANTHINE	C21H26N2O3	354.45		*bark of Pseudocinchona africana Chev.*	2
01500332	HOMATROPINE BROMIDE	C16H22BrNO3	356.26	anticholinergic (opthalmic)	semisynthetic	1
01500807	ADENINE	C5H5N5	135.12	Vitamin B4	widespread in nature	1
00270067	STIGMASTA-4.22-DIEN-3-ONE	C29H46O	410.68		plant constituent	1
01500909	PROTOVERATRINE A	C41H63NO14	793.95	antihypertensive. emetic; LD50 (rat) 5 mg/kg po	*Veratrum album. V viride. V nigrum*	1
01300027	IVERMECTIN	C48H74O14	875.11	antiparasitic	semisynthetic	1

01505248	CANRENONE	C22H28O3	340.466	aldosterone antagonist; antifibrogenic	synthetic	1
00200793	IRIDIN	C24H26O13	522.46		*Iris spp.*	1
00390001	APIOLE	C12H14O4	222.24	antipyretic. diuretic. insecticide	*Petroselimum spp. Anethum graveolens*	1
00100615	CHUKRASIN METHYL ETHER	C43H58O16	830.93		Meliaceae	1
01505263	ALFLUZOSIN	C19H27N5O4	389.45	alpha(1)-adrenergic blocker	synthetic; SL-77499	1
01300029	MEGLUMINE	C7H17NO5	195.21	diagnostic aid	semisynthetic	1
01900040	BARBITAL	C8H12N2O3	184.19	sedative	synthetic	1
00100616	ANGOLENSIN (R)	C16H16O4	272.30		*Pericopsis and Pterocarpus spp*	1
01400131	HAEMATOMMIC ACID	C9H8O5	196.16		*Alectoria spp. Haematomma spp and Lethariella spp*	1
01505709	VECURONIUM BROMIDE	C34H57BrN2O4	637.74	neuromuscular blocker	synthetic; ORG-NC-45	1
02300309	VESAMICOL HYDROCHLORIDE	C17H26ClNO	295.85	acetylcholine transport blocker (vesicular)	synthetic	1
00100537	SOLASODINE	C27H43NO2	413.64	antineoplastic. antiinflammatory	numerous Solanum spp	1
01505140	2'.4-DIHYDROXY-3.4'.6'-TRIMETHOXYCHALCONE	C18H18O6	330.34		*Viscum album (glucoside)*	1
01505109	MANGANESE TETRAKIS(4-CARBOXYPHENYL)PORPHYRIN CHLORDE	C48H28ClMnN4O8	879.17	SOD mimetic	synthetic	1
01500841	CINCHONINE	C19H22N2O	294.39	antimalarial	*Cinchona spp*	1
01502096	alpha-CYANO-4-HYDROXYCINNAMIC ACID	C10H7NO3	189.17	inhibitor of mitochondrial pyruvate transport	synthetic	1
01502070	6.7-DICHLORO-3-HYDROXY-2-QUINOXALINECARBOXYLIC ACID	C9H4Cl2N2O3	259.04	NMDA and kainate receptor antagonist	synthetic	1
01504211	CHLOROGUANIDE HYDROCHLORIDE	C11H17Cl2N5	290.19	antimalarial	synthetic	1
00100517	ENTANDROPHRAGMIN	C43H56O17	844.91		*Entangrophragma spp.*	1
01500739	CHRYSIN DIMETHYL ETHER	C17H14O4	282.29		*Boesenburgia pandurata. Leptosermum scoparium*	1
01500395	METHICILLIN SODIUM	C17H19N2NaO6S	402.40	antibacterial	semisynthetic	1
01505276	CAPSANTHIN	C40H56O3	584.89	antineoplastic	*Capsicum annum*	1
01506069	FINASTERIDE	C23H36N2O2	372.55	anti-androgen. alpha-reductase inhibitor	synthetic; MK=909	1
01502041	CEFAMANDOLE NAFATE	C19H17N6NaO6S2	512.50	antibacterial	semisynthetic	1
01505468	UNDECYLENIC ACID	C11H20O2	184.28	antifungal	semisynthetic	1

01505241	SPARTEINE HYDROIODIDE	C15H27IN2	362.30	oxytocic	*Lupinus spp and other Leguminosae*	1
01500269	DYPHYLLINE	C10H14N4O4	254.24	PDE inhibitor. bronchodilator. vasodilator	synthetic	1
01505514	BIPERIDEN	C21H29NO	311.47	anticholinergic. antiparkinsonia n	synthetic	1
01505992	BUFLOMEDIL HYDROCHLORIDE	C17H26ClNO4	343.85	vasodilator (peripheral)	synthetic	1
01505361	MODAFINIL	C15H15NO2S	273.35	analeptic	synthetic; CRL-40476. CEP-1538	1
01500685	CLOZAPINE	C18H19ClN4	326.83	antipsychotic	synthetic	1
01505299	AMITRAZ	C19H23N3	293.41	scabicide	synthetic; U-36059	1
01505639	TADALAFIL	C22H19N3O4	389.41	erectile dysfunction therapy	synthetic; IC-351	1
00300604	PHLORACETOPHENONE	C8H8O4	168.15		*Prunus domesticus*	1
01502006	CARPROFEN	C15H12ClNO2	273.72	antiinflammator y. analgesic	synthetic	1
01505312	TYLOSIN TARTRATE	C46H77NO17	916.12	antibacterial	*Streptomyces fradiae*	1
01506049	meta-CRESYL ACETATE	C9H10O2	150.17	antiseptic (topical)	synthetic	1
01505325	SOLANESOL	C45H74O	631.09		*Nicotiana tabacum; Betulaprenol 9: mp 42 C*	1
01500674	MYCOPHENOLIC ACID	C17H20O6	320.34	antineoplastic	*Penicillium brevicompactu m and other Penicillium spp*	1
01505762	PYRITINOL	C16H20N2O4S2	368.47	cognition enhancer. nootropic	synthetic; pyrithioxin	1
00300048	KOBUSONE	C14H22O2	222.33		*Cyperus rotundus. Sindora sumatrana*	1
01500163	CEFADROXIL	C16H17N3O5S	363.39	antibacterial	semisynthetic	1
01500668	KETOTIFEN FUMARATE	C23H23NO5S	425.50	antiasthmatic	synthetic	1
01503245	LEVAMISOLE HYDROCHLORIDE	C11H13ClN2S	240.75	immunomodula tor	synthetic	1
01505216	ESCITALOPRAM OXALATE	C22H23FN2O5	414.43	antidepressant. 5HT reuptake inhibitor	synthetic; LU26-054-0	1
01505727	MELENGESTROL ACETATE	C25H32O4	396.53	antineoplastic. progestin	semisynthetic; BDH-1921. 5373. NSC-70968	1
01504180	ASARININ (-)	C20H18O6	354.36	antibacterial (tuberculostatic)	Asarum & Xanthoxylum spp	1
01505683	ROLIPRAM	C16H21NO3	275.35	tranquilizer	synthetic; ZK-62711	1
00100583	EUPHOL ACETATE	C32H52O2	468.77		Euphorbia spp	1
01501204	LAPACHOL	C15H14O3	242.27	antineoplastic. antifungal	heartwood of Bignoniaceae	1

01505107	VALINOMYCIN	C54H90N6O18	1111.39	antibiotic; LD50 (rat. po) 4 mg/kg	*Streptomyces spp*	1
01503077	PRIDINOL METHANESULFONATE	C21H29NO4S	391.53	anticholinergic	synthetic; C-238	1
01505745	ANDROSTERONE	C19H30O2	290.44	androgen	male urine	1
01503038	NICOTINYL ALCOHOL TARTRATE	C10H13NO7	259.21	vasodilator	synthetic	1
01500372	MAFENIDE HYDROCHLORIDE	C7H11ClN2O2S	222.69	antibacterial	synthetic	1
01503804	COLFORSIN	C22H34O7	410.51	adenylate cyclase activator. antiglaucoma. hypotensive. vasodilator	*Coleus forskohlii; HL-362; L-75-1362B*	1
00100572	FRAXIDIN METHYL ETHER	C12H12O5	236.22		derivative	1
00203008	JUAREZIC ACID	C11H10O2	174.20		*propolis and Populus spp*	1
00212064	HYDROXYTOLUIC ACID	C8H8O3	152.15	analgesic. antiseptic	synthetic; 3-MS	1
01502235	ASTAXANTHIN	C40H52O4	596.85		Carotenoid pigment plant and animal sources	1
01505342	7-NITROINDAZOLE	C7H5N3O2	163.13	NO synthetase inhibitor	synthetic	1
01600025	2.6-DIHYDROXY-4-METHOXYTOLUENE	C8H10O3	154.16		synthetic	1
00300053	BROMO-3-HYDROXY-4-(SUCCIN-2-YL)-CARYOLANE gamma-LACTONE	C19H27BrO4	399.32		derivative of caryophyllene	1
01505345	CURCUMIN	C21H20O6	368.39	antiedemic. antiinflammatory. bile stimulant; antibacterial. antifungal. lipo/cyclooxygenase inhibitor	*Curcuma spp*	1
01500459	PAPAVERINE HYDROCHLORIDE	C20H22ClNO4	375.85	muscle relaxant (smooth). cerebral vasodilator	*Papaver somniferum. Rauwolfia serpentina*	1
01505778	BEPHENIUM HYDROXYNAPTHOATE	C28H29NO4	443.54	anthelmintic	synthetic	1
01501137	NEFOPAM	C17H19NO	253.34	analgesic	synthetic	1
01505399	OXYBUTYNIN CHLORIDE	C22H32ClNO3	393.95	anticholinergic	synthetic; MJ-4309-1. 5058	1
01505396	FLUORESCEIN	C20H12O5	332.31	corneal trama indicator	synthetic	1
01500626	DICHLOROPHENE	C13H10Cl2O2	269.12	anthelmintic	synthetic	1
01503721	ENROFLOXACIN	C19H22FN3O3	359.40	antibacterial	synthetic	1
01502036	VIGABATRIN	C6H11NO2	129.16	anticonvulsant	synthetic; MDL-71754	1
01500242	DIETHYLCARBAMAZINE CITRATE	C16H29N3O8	391.42	anthelmintic	synthetic	1
00270083	5alpha-CHOLESTAN-3beta-OL-6-ONE	C27H46O2	402.66		*Mandevilla pentlandiana*	1
01500418	MOXALACTAM DISODIUM	C20H18N6Na2O9S	564.44	antibacterial	semisynthetic	1

01505213	ROSUVASTATIN CALCIUM	C22H27CaFN3O6S	520.61	antihyperlipidemic	synthetic	1
00100139	DEOXYKHIVORIN	C32H42O9	570.68		*Meliaceae spp*	1
01500903	ETOPOSIDE	C29H32O13	588.57	antineoplastic	semisynthetic	1
01500123	AMPICILLIN SODIUM	C16H18N3NaO4S	371.39	antibacterial	semisynthetic	1
01504517	DOXIFLURIDINE	C9H11FN2O5	246.19	antineoplastic	synthetic	1
00380004	DIHYDROCELASTRYL DIACETATE	C33H44O6	536.71	chaperone stimulant	celastrol derivative	1
01500690	MELATONIN	C13H16N2O2	232.28	sleep induction. modifies circadian rhythm	pineal gland	1
01500316	GLUCOSAMINE HYDROCHLORIDE	C6H14ClNO5	215.63	antiarthritic	polysaccharides in bacteria. fungi. higher plants. invertebrates. vertebrates	1
01500427	NEOMYCIN SULFATE	C23H48N6O17S	712.73	antibacterial	*Streptomyces fradiae*	1
01503816	AMCINONIDE	C28H35FO7	502.58	glucocorticoid. antiinflammatory	synthetic	1
01505180	6.2'-DIMETHOXYFLAVONE	C17H14O4	282.29		derivative	1
01503346	URIDINE TRIPHOSPHATE TRISODIUM	C9H12N2Na3O15P3	550.09	psychostimulant	yeast	1
01500166	CEPHALOTHIN SODIUM	C16H15N2NaO6S2	418.42	antibacterial	semisynthetic	1
01505340	AZELASTINE HYDROCHLORIDE	C22H25Cl2N3O	418.37	H1 antihistamine (nonsedating); leucotriene synthesis blocker	synthetic	1
01505996	AZTREONAM	C13H21N5O7S2	423.46	antibacterial	synthetic; SQ-26776	1
01401401	METHYL ROBUSTONE	C22H18O6	378.38		*Derris spp*	1
01505154	3-AMINO-1.2.4-TRIAZOLE	C2H4N4	84.08	catalase inhibitor	synthetic	1
01500116	AMINOSALICYLATE SODIUM	C7H6NNaO3	175.12	antibacterial. tuberculostatic	synthetic	1
02300325	METOLAZONE	C16H16ClN3O3S	365.84	diuretic. antihypertensive	synthetic	1
01500590	TRICHLORMETHIAZIDE	C8H8Cl3N3O4S2	380.65	diuretic. antihypertensive	synthetic	1
01502248	GLUTATHIONE	C10H17N3O6S	307.32	antioxidant	plant and animal tissue	1
01500873	PIPERINE	C17H19NO3	285.34	analeptic. antibacterial	black pepper (Piper nigrum L.)	1
01502013	ISOTRETINON	C20H28O2	300.44	antiacne. antineoplastic	semisynthetic	1
01505763	RIBOFLAVIN 5-PHOSPHATE SODIUM	C17H20N4NaO9P	478.33	vitamin. enzyme cofactor	wdiespread in plants and animals	1
00240929	AVOCADYNE ACETATE	C19H34O4	326.48	antifungal	*Persea spp*	1
01505240	LUPANYL ACID HYDROCHLORIDE	C14H25ClN2O2	288.82		*Anabasis aphylla*	1

01500144	BETAMETHASONE	C22H29FO5	392.47	glucocorticoid. antiinflammatory	semisynthetic	1
01501103	MEFENAMIC ACID	C15H15NO2	241.29	antiinflammatory. analgesic	synthetic	1
01505387	DULOXETINE HYDROCHLORIDE	C18H20ClNOS	333.88	antidepressant	synthetic; LY-248686	1
01504225	2-MERCAPTOBENZOTHIAZOLE	C7H5NS2	167.25	antifungal	synthetic; AG-63	1
01401414	CRESOPIRINE	C10H10O4	194.18	antiinflammatory. antpyretic	synthetic	1
01501145	SULFAQUINOXALINE SODIUM	C14H11N4NaO2S	322.32	antibacterial	synthetic	1
01500550	SULFAMETHOXAZOLE	C10H11N3O3S	253.28	antibacterial. antipneumocystis	synthetic	1
00300533	ISOSAFROLE	C10H10O2	162.19		*Illicium religiosum*	1
01504041	TRIACETYLRESVERATROL	C20H18O6	354.36		*Kirkpatrickia variolosa (sponge)*	1
01500730	4-METHYLDAPHNETIN	C10H8O4	192.17	estrogen. antineoplastic (hormonal)	analog of daphnetin	1
01502130	AMINOCYCLOPROPANECARBOXYLIC ACID	C4H7NO2	101.10	NMDA partial agonist (gly)	synthetic; ACPC	1
01500270	TRISODIUM ETHYLENEDIAMINE TETRACETATE	C10H16N2Na3O8	361.21	chelating agent. antioxidant	synthetic	1
00300106	3-AMINO-beta-PINENE	C10H18ClN	187.71		derivative	1
00300056	LARIXOL	C20H34O2	306.49		*Larix sibiricia*	1
01500460	PARACHLOROPHENOL	C6H5ClO	128.55	topical antibacterial (topical)	synthetic	1
01500778	LIOTHYRONINE	C15H12I3NO4	650.98	thyroid hormone blocker	semisynthetic	1
00100346	PICROTIN	C15H18O7	310.30	GABAa receptor antagonist	nontoxin component of PICROTOXIN	1
00310011	HESPERIDIN	C28H34O15	610.57	capillary protectant	*Citrus spp.*	1
01504051	STIGMASTEROL	C29H48O	412.70		soya and calabar beans; widely distributed in plant oils	1
00300545	CHRYSOPHANOL	C15H10O4	254.24		*Cassia and Rumex spp*	1
01504013	UBIDECARENEONE	C59H90O4	863.37	cardiovascular agent	widespread in aerobic organisms (solubility limits concentration to ~5mM); COENZYME Q10	1
01500439	NORETHYNODREL	C20H26O2	298.42	progestogen. in combination with estrogen as	synthetic	1

				oral contraceptive		
00210658	DEHYDROVARIABILIN	C17H14O4	282.29		*Dalbergia variabilis*	1
01500312	GALLAMINE TRIETHIODIDE	C30H60I3N3O3	891.54	muscle relaxant (skeletal)	synthetic	1
01500275	EQUILIN	C18H20O2	268.35	estrogen	pregnant mare urine	1
01505862	2-AMINOBENZENESULFONAMIDE	C6H8N2O2S	172.20	antibacterial	synthetic	1
01503994	CONVALLATOXIN	C29H42O10	550.65	cardiotonic	Convallaria majalis	1
01500526	RESERPINE	C33H40N2O9	608.69	antihypertensive	*Rauwolfia serpentina*	1
01502039	FOSFOMYCIN CALCIUM	C3H5CaO4P	176.12	antibacterial	*Streptomyces spp*	1
01505449	PRALIDOXIME CHLORIDE	C7H9ClN2O	172.61	cholesterase agonist	synthetis; 2-PAM	1
00307056	MUUROLLADIE-3-ONE	C15H22O	218.34		derivative of muurolene	1
01504525	OROTIC ACID	C5H4N2O4	156.09	hepatoprotectant. uricosuric agent	widespread in animals	1
01504258	NATEGLINIDE	C19H27NO3	317.43	antidiabetic	synthetic	1
01500294	CINEOLE	C10H18O	154.25	anthelminthic. antiseptic. expectorant	eucalyptus and lavender oils	1
01501110	MEBENDAZOLE	C16H13N3O3	295.30	anthelmintic	synthetic	1
01505374	VARDENAFIL HYDROCHLORIDE	C23H33ClN6O4S	525.07	erectile dysfunction. PD5 inhibitor	synthetic	1
01505677	TICLOPIDINE HYDROCHLORIDE	C14H15Cl2NS	300.25	PAF inhibitor	synthetic; 53-32C. 4-C-32	1
01505972	BROMPERIDOL	C21H23BrFNO2	420.32	antipsychotic	synthetic; R-11333	1
01500758	LOBELINE HYDROCHLORIDE	C22H28ClNO2	373.92	antiasthmatic. respiratory stimulant	*Lobelia spp*	1
01500443	NOSCAPINE HYDROCHLORIDE	C22H24ClNO7	449.89	antitussive	*Corydalis cava and Papaver spp*	1
01506027	OXALAMINE CITRATE	C20H27N3O8	437.45	antitussive	synthetic; SKF-9976. AF-438	1
00201696	LANOSTEROL ACETATE	C32H52O2	468.77		derivative of lanosterol	1
00200640	3-METHYLORSELLINIC ACID	C9H10O4	182.17		*Aspergillus terreus*	1
00500580	PIPERONYLIC ACID	C8H6O4	166.13		*Piper longum. Cinnamumum spp*	1
00100096	3-DEOXY-3beta-HYDROXYANGOLENSIC ACID METHYL ESTER	C27H36O7	472.58		*Meliaceae spp*	1
01502242	SCOPOLETIN	C10H8O4	192.17	NO synthesis (inducible) inhibitor. anticoagulant	*Scopolia spp.*	1
01501113	PERUVOSIDE	C30H44O9	548.67	cardiotonic	*Thevetia peruviana*	1

01504614	GYROMITRIN	C4H8N2O	100.12	hepatotoxin. carcinogen	*Gyromitra mushroom species; metabolized to to the hepatotoxin formylmethylhydrazine*	1
00300029	DESOXYCORTICOSTERONE ACETATE	C23H32O4	372.50	mineralocorticoid	adrenocortex	1
01500614	XYLOMETAZOLINE HYDROCHLORIDE	C16H25ClN2	280.84	adrenergic agonist. nasal decongestant	synthetic	1
01501117	MEBEVERINE HYDROCHLORIDE	C25H36ClNO5	466.02	muscle relaxant (smooth)	synthetic	1
01503204	CINNARAZINE	C26H28N2	368.52	H1 antihistamine	synthetic	1
00201604	PYRROMYCIN	C30H35NO11	585.61	antibacterial	*Streptomyces spp*	1
01500126	ANTAZOLINE PHOSPHATE	C17H22N3O4P	363.35	antihistaminic	synthetic	1
01500387	MERCAPTOPURINE	C5H4N4S	152.17	antineoplastic. purine antimetabolite	synthetic	1
01503678	CELECOXIB	C17H14F3N3O2S	381.37	antiarthritic. cyclooxygenase2 inhibitor	synthetic; SC-58635	1
01504123	10-HYDROXYCAMPTOTHECIN	C20H16N2O5	364.36	antineoplastic	*Camptotheca acuminata*	1
01500871	NORHARMAN	C11H8N2	168.19	plant growth inhibitor; mutagen	*Chrysophyllum and Nocardia spp*	1
01500158	CARBACHOL	C6H15ClN2O2	182.65	cholinergic. miotic	synthetic	1
01500555	SULFISOXAZOLE	C11H13N3O3S	267.30	antibacterial	synthetic	1
00200046	GRISEOFULVIN	C17H17ClO6	352.77	antifungal. inhibits mitosis in metaphase	*Penicillium griseofulvum*	1
01500655	ACONITINE	C34H47NO11	645.75	anesthetic (gastric). antipyretic. and cardiotoxin	*Aconitum spp*	1
01500435	NITROMIDE	C7H5N3O5	211.13	antibacterial. coccidiostat	synthetic	1
01504085	PAROXETINE HYDROCHLORIDE	C19H21ClFNO3	365.83	antidepressant	synthetic	1
00310025	LARIXINIC ACID	C6H6O3	126.11		Larix decidua	1
01505038	KASUGAMYCIN HYDROCHLORIDE	C14H26ClN3O9	415.83	antifungal	Streptomyces kasugaensis	1
01504145	GLICLAZIDE	C15H21N3O3S	323.41	antidiabetic. adhesion inhibitor	synthetic; SE-1702	1
01502021	PHTHALYLSULFATHIAZOLE	C17H13N3O5S2	403.43	antibacterial	synthetic	1
01502127	TINIDAZOLE	C8H13N3O4S	247.27	antiprotozoal	synthetic	1
01501151	RANITIDINE	C13H22N4O3S	314.40	H2 antihistamine	synthetic	1
01500279	ERYTHROMYCIN ETHYLSUCCINATE	C43H75NO16	862.07	antibacterial	semisynthetic	1
00200759	IRETOL	C7H8O4	156.13		semisynthetic	1
00211468	DANTHRON	C14H8O4	240.21	cathartic	*Rheum palmatum.*	1

					Xyris semifuscata	
01505321	RIFAXIMIN	C43H51N3O11	785.89	antibacterial. RNA synthesis inhibitor	semisynthetic	1
01505187	QUETIAPINE	C21H25N3O2S	383.51	antipsychotic: 5HT antagonist. dopamine antagonist. H1-antihistamine. alpha adrenergic blocker	synthetic; ICI-204636. ZD-5077. ZM-204639	1
01503805	DESACETYLCOLFORSIN	C20H32O6	368.47		*Coleus forskohlii*; 8.13-epoxy-1.6.7.9-tetrahydroxy-14-labden-11-one	1
01505104	4-(3-BUTOXY-4-METHOXYBENZYL)IMIDAZOLIDIN-2-ONE	C15H22N2O3	278.35	cAMP PDE inhibitor. inhibits cellular adhesion and superoxide & platelet aggregation	synthetic; RO-20-1724	1
01500699	ACETYLTRYPTOPHANAMIDE	C13H15N3O2	245.28	antidepressant. nutrient	synthetic	1
01503986	CAFESTOL ACETATE	C22H30O4	358.48		coffee bean oil	1
01505189	RIZATRIPTAN BENZOATE	C22H25N5O2	391.47	5HT-1B/1D agonist. antimigraine	synthetic; MK-0462 (benzoate)	1
01503100	URAPIDIL HYDROCHLORIDE	C20H30ClN5O3	423.94	antihypertensive	synthetic	1
00100012	DEACETYLGEDUNIN	C26H32O6	440.54		*Khaya. Azadirachta and other West African timbers; mp 264-266*	1
01505392	TERBINAFINE HYDROCHLORIDE	C21H26ClN	327.90	antifungal	synthetic. SF-86-327	1
01501173	ACETANILIDE	C8H9NO	135.16	analgesic. antipyretic	synthetic	1
00300132	3.7-EPOXYCARYOPHYLLAN-6-ONE	C15H24O2	236.35		*derivative Lippia spp*	1
00100298	SMILAGENIN ACETATE	C29H46O4	458.68		*Smilax ornata. Agave & Yucca spp*	1
01503256	AMSACRINE	C22H20N2O3S	392.48	antineoplastic. immune suppressive	synthetic	1
01505433	GLIPIZIDE	C21H27N5O4S	445.54	antidiabetic	synthetic; CP-28720. K-4024	1
01503648	GABOXADOL HYDROCHLORIDE	C6H9ClN2O2	176.60	GABAa agonist. GABAc antagonist	synthetic; THIP hydrochloride	1
00200523	XANTHONE	C13H8O2	196.20		gentian and other flowers	1
00100513	PTAEROXYLIN	C15H14O4	258.27		*Ptaeroxylon obliquum. Cedrelopsis grevei*	1

00100081	UTILIN	C41H52O17	816.86		*Entandrophragma utile*	1
01505397	NIACINAMIDE	C6H6N2O	122.12	Vitamin B3; enzyme cofactor; anti-pellagra	widespread in plants. yeasts. fungi	1
01505114	CLIOQUINOL	C9H5ClINO	305.50	antiseptic. antiamebic	synthetic	1
01503227	PERHEXILINE MALEATE	C23H39NO4	393.57	coronary vasodilator	synthetic	1
01500582	TOLMETIN SODIUM	C15H14NNaO3	279.27	antiinflammatory	synthetic	1
01501172	AZOBENZENE	C12H10N2	182.22	acaricide	synthetic	1
01501010	FENOFIBRATE	C20H21ClO4	360.84	antihyperlipidemic	synthetic	1
01504210	ATOVAQUONE	C22H19ClO3	366.84	antipneumocystic. antimalarial	synthetic	1
00100551	FRIEDELIN	C30H50O	426.73		*Ceratopetalum apetalum D. Don. Cunoniaceae*	1
01502004	BUMETANIDE	C17H20N2O5S	364.42	diuretic	synthetic	1
01505371	CETIRIZINE HYDROCHLORIDE	C21H27Cl3N2O3	461.81	H1 antihistamine	synthetic	1
01503069	AMINOHIPPURIC ACID	C9H10N2O3	194.19	renal function diagnosis	synthetic	1
00501332	PHENACYLAMINE HYDROCHLORIDE	C8H10ClNO	171.62		*Castanopsis cuspidata. Vitis spp*	1
01505010	EUPHORBIASTEROID	C32H40O8	552.67		*Euphorbia lathyris. Macaranga tanarius*	1
01501019	BERBAMINE HYDROCHLORIDE	C37H42Cl2N2O6	681.66	antihypertensive. skeletal muscle relaxant	*Berberis spp*	1
02300176	PHENOXYBENZAMINE HYDROCHLORIDE	C18H23Cl2NO	340.29	alpha adrenergic blocker	synthetic	1
01504511	CHLORALOSE	C8H11Cl3O6	309.53	anesthetic	synthetic	1
01300046	TYROSINE	C9H11NO3	181.19		widespread in nature	1
00100375	alpha-DIHYDROGEDUNOL	C28H38O7	486.61		derivative	1
00201602	PACHYRRHIZIN	C19H12O6	336.30	insecticide	*Pachyrrhizus erosus; mp 206-207 C*	1
01400164	2-HYDROXY-3.4-DIMETHOXYBENZOIC ACID	C9H10O5	198.17	prostaglandin synthetase inhibitor	*Dalbergia odorifera*	1
01502227	CADAVERINE TARTRATE	C7H16N2O6	224.21		putrifaction of lysine; sectrtion of the fox *Vulpes vulpes*	1
01505758	SKATOLE	C9H9N	131.17	insect attractant	feces. beetroot. nectandra wood	1
01500224	DEFEROXAMINE MESYLATE	C26H52N6O11S	656.80	chelating agent (Fe & Al)	*Strptomyces pilosus*	1
01504136	ALAPROCLATE	C13H18ClNO2	255.74	antidepressant	synthetic	1
00211949	APOTOXICAROL	C18H14O7	342.30		derivative	1

01506086	SULFADOXINE	C12H14N4O4S	310.33	antibacterial	synthetic; RO-4-4393	1
01500109	ALVERINE CITRATE	C26H35NO7	473.57	anticholinergic	synthetic	1
01500112	AMILORIDE HYDROCHLORIDE	C6H9Cl2N7O	266.09	Na+ channel inhibitor. diuretic	synthetic	1
01505801	TOPIRAMATE	C12H21NO8S	339.36	anticonvulsant. antimigraine. GABA-A agonist. AMP/kinate glutamate receptor antagonist. carbonic anhydrase inhibitor	synthetic; RWJ-17021	1
01505210	SIBUTRAMINE HYDROCHLORIDE	C17H27Cl2N	316.31	anorexic. antidepressant. uptake inhibitor (5HT. norepinephrine. dopamine)	synthetic	1
00211249	7.4'-DIMETHOXYISOFLAVONE	C17H14O4	282.29		*Dalbergia violaceae. Pterodon apparicioi*	1
01505987	DENATONIUM BENZOATE	C28H34N2O3	446.59	denaturing agent. bitter principle	synthetic	1
01600964	N-METHYLANTHRANILIC ACID	C8H9NO2	151.16		grapefruit peel oil	1
01503239	HYCANTHONE	C20H24N2O2S	356.49	anthelmintic. hepatotoxic	synthetic	1
00310298	GLUCOSAMINIC ACID	C6H13NO6	195.17		oxidation product of glucosamine (01500316)	1
00100529	HETEROPEUCENIN. METHYL ETHER	C16H18O4	274.31		*Ptaeroxylon obliquum. Harrisonia perforate .Neochamaelea pulverulenta.*	1
01504076	YOHIMBIC ACID HYDRATE	C20H26N2O4	358.44		derivative; yohimbinic acid	1
01505576	TERAZOSIN HYDROCHLORIDE	C19H26ClN5O4	423.90	antihypertensive	synthetic; Abbott-45975	1
00201154	DEHYDROROTENONE	C23H20O6	392.41		*Derris spp; rotenone derivative*	1
01504234	18-AMINOABIETA-8.11.13-TRIENE SULFATE	C20H33NO4S	383.55		derivative of abietic acid	1
01500232	DEXAMETHASONE SODIUM PHOSPHATE	C22H28FNa2O8 P	516.41	glucocorticoid. antiinflammator y	semisynthetic	1
01505037	3-BROMO-4-METHYL-3.4-HEXAMETHYLENE-3.4-DIHYDRODIAZETE-1.2-DIOXIDE	C7H11BrN2O2	235.08	guanyl cyclase activator. NO donor. vasodilator	synthetic; DD2	1
01500721	7.4'-DIHYDROXYFLAVONE	C15H10O4	254.24	antioxidant	*Pterocarpus marsupium*	1

01504215	N.N-HEXAMETHYLENEAMILORIDE	C12H18ClN7O	311.77	Na/H+ antiporter inhibitor. diuretic	synthetic	1
01501140	MEPHENESIN	C10H14O3	182.22	muscle relaxant (skeletal)	synthetic	1
01503500	RESORCINOL MONOACETATE	C8H8O3	152.15	antiseborrheic. antipruritic	synthetic	1
01505898	HEMICHOLINIUM BROMIDE	C24H34Br2N2O4	574.35	acetylcholine antagonist	synthetic	1
00307047	3-OXOURSAN (28-13)OLIDE	C30H44O3	452.68		semisynthetic	1
01504524	ORNITHINE	C5H12N2O2	132.16	hepatoprotectant. anticholesteremic	widespread in nature	1
01505336	VALERYL SALYCILATE	C12H14O4	222.24	COX-1 inhibitor	synthetic	1
01502030	OXOLINIC ACID	C13H11NO5	261.23	antibacterial	synthetic	1
01500573	THIOGUANINE	C5H5N5S	167.19	antineoplastic. purine antimetabolite	synthetic	1
01501132	NEROL	C10H18O	154.25	weak estrogen receptor blocker	neroli and bergamot oils	1
01505209	VALSARTAN	C24H29N5O3	435.53	Angiotensin II inhibitor. antihypertensive	synthetic; CGP-48933	1
01500185	CHLORPROPAMIDE	C10H13ClN2O3S	276.74	antidiabetic	synthetic	1
01500486	PHYSOSTIGMINE SALICYLATE	C22H27N3O5	413.47	cholinergic. anticholinesterase. miotic	*Physostigma venenosum*	1
01300010	ARGININE HYDROCHLORIDE	C6H15ClN4O2	210.66	ammonia detoxicant. diagnostic aid	widespread in nature	1
01504104	DIHYDROJASMONIC ACID	C12H20O3	212.29	plant growth regulator	*Jasminium spp and Vicia faba*	1
01500901	AESCULIN	C15H16O9	340.28	antiinflammatory	*Aesculus hippocastanum. Fraxinus spp*	1
01504053	FUCOSTANOL	C29H52O	416.73		*Calendula officinalis*	1
01505984	PROTRYPTYLINE HYDROCHLORIDE	C19H22ClN	299.84	antidepressant	synthetic; MK-240	1
00310001	ACONITIC ACID	C6H6O6	174.11		*Aconitum and Achillea spp*	1
01502256	SHIKIMIC ACID	C7H10O5	174.15		common constituent in plants	1
01502232	CAMPTOTHECIN	C20H16N2O4	348.36	antineoplastic	*Camptotheca acuminata*	1
01500558	TERBUTALINE HEMISULFATE	C12H21NO7S	323.36	betaadrenergic agonist. bronchodilator	synthetic	1
01500164	CEFAZOLIN SODIUM	C14H13N8NaO4S3	476.49	antibacterial	semisynthetic	1
01300038	SODIUM OXYBATE	C4H7NaO3	126.08	anesthetic	synthetic; WY-3478. NSC-84223	1
02300307	VERAPAMIL HYDROCHLORIDE	C27H39ClN2O4	491.07	adrenegic blocker. Ca channel blocker. coronary	synthetic	1

				vasodilator. antiarrhythmic		
01503614	CIPROFLOXACIN	C17H18FN3O3	331.34	antibacterial. fungicide	synthetic	1
01505327	CHLORMADINONE ACETATE	C23H29ClO4	404.93	progestin. antiandrogen	semisynthetic	1
01503339	SULFANITRAN	C14H13N3O5S	335.34	antibacterial	synthetic; NSC-77120	1
01500143	beta-CAROTENE	C40H56	536.89	provitamin A	provitamin A; widespread in plants and animals	1
01503985	BROMPHENIRAMINE MALEATE	C20H23BrN2O4	435.32	H1 antihistamine	synthetic	1
01505895	GLYCOCHOLIC ACID	C26H43NO6	465.63		mammalian bile	1
00350025	APIIN	C26H28O14	564.50		parsley seed; flowers of *Anthemis nobilis*. 80% + other glycosides	1
01505254	THEANINE	C7H14N2O3	174.20		*Thea sinensis*	1
01503609	NITRENDIPINE	C18H20N2O6	360.36	antihypertensive	synthetic	1
00211175	METACETAMOL	C8H9NO2	151.16	analgesic	synthetic; BS-749	1
00100117	HYDROLYSIS PRODUCT OF BUSSEIN	C32H40O14	648.66		structure tentative	1
01500291	ETHINYL ESTRADIOL	C20H24O2	296.41	estrogen. plus progestogen as oral contraceptive	semisynthetic	1
01505168	ETHACRIDINE LACTATE	C18H21N3O4	343.38	antiseptic. abortifaceant	synthetic	1
01500375	MECHLORETHAMINE	C5H11Cl2N	156.05	antineoplastic. alkylating agent	synthetic	1
01500404	METHYLERGONOVINE MALEATE	C24H29N3O6	455.51	oxytocic	semisynthetic	1
01500379	MEDROXYPROGESTERONE ACETATE	C24H34O4	386.53	contraceptive	semisynthetic	1
01502019	FOSCARNET SODIUM	CNa3O5P	191.95	antiviral	synthetic	1
01500811	BERBERINE CHLORIDE	C20H18ClNO4	371.82	antiarrhythmic. alpha2 agonist. cholinesterase. anticonvulsant. antiinflammatory. antibacterial. antifungal. antitrypanosomal. antineoplastic. immunostimulant	*Berberis and Mahonia spp*	1
01500216	CYPROTERONE ACETATE	C24H29ClO4	416.94	antiandrogen	synthetic	0
01500819	BERGENIN	C14H18O10	346.29	hepatoprotectant	*Bergenia spp.*	0
01505165	2-METHYL-4-(PIPERIDIN-1-YLCARBOXY)-5-ISOPROPYLPHENYLTRIMETHYLAMMONIUM CHLORIDE	C19H31ClN2O2	354.92	squalene-2.3-oxide cyclase inhibitor. anticholesterolemic	synthetic	0

01500847	CHOLESTEROL	C27H46O	386.66	emulsifying agent	common animal sterol	0
01505703	ALTRENOGEST	C21H26O2	310.44	progestinantine oplastic	synthetic; RU-2267. A-35957	0
01500412	METRONIDAZOLE	C6H9N3O3	171.15	antiprotozoal	synthetic	0
01501153	SULOCTIDIL	C20H35NOS	337.57	peripheral vasodilator	synthetic	0
01504150	MELOXICAM SODIUM	C14H12N3NaO4 S2	373.38	antiinflammator y	synthetic	0
01503059	FLOXURIDINE	C9H11FN2O5	246.19	antineoplastic. antimetabolite	synthetic	0
01503114	DIBEKACIN	C18H39N5O12S	549.60	antibacterial	semisynthetic	0
01503105	BENFOTIAMINE	C19H23N4O6PS	466.45	vitamin B1	Vitamin B1; 8088-CB	0
00100014	7-DEACETYLKHIVORIN	C30H40O9	544.64		*Khaya species and other West African timbers*	0
00300003	SOLIDAGENONE	C20H28O3	316.44		*Solidago canadensis L.; mp 133-134 C*	0
00330058	CHLORPYRIFOS	C9H11Cl3NO3P S	350.58	insecticide	synthetic; DURSBAN	0
01505991	DECAMETHONIUM BROMIDE	C16H38Br2N2	418.30	neuromuscular blocker	synthetic	0
01500180	CHLOROTHIAZIDE	C7H6ClN3O4S2	295.72	diuretic. antihypertensive	synthetic	0
01500205	COLCHICINE	C22H25NO6	399.44	antimitotic. antigout agent	*Colchicum autumnale*	0
01502150	CARBIDOPA	C10H14N2O4	226.23	decarboxylase inhibitor. antiparkinsonis m	synthetic	0
01503301	SULFANILATE ZINC	C12H12N2O6S2 Zn	409.73	antibacterial	synthetic	0
02300347	PENTYLENETETRAZOL	C6H10N4	138.17	analeptic. circulation stimulant	synthetic	0
00100318	DIOSGENIN	C27H42O3	414.63	antiinflammator y. estrogen; LD50(rat) 4872 mg/kg ip. LD50 (mouse) >8000 mg/kg po	*Clintonia. Dioscorea and Solanum spp. Trillium erectum. Balanites aegyptiaca. Aletris farinosa*	0
01506003	KAEMPFEROL	C15H10O6	286.24		*Citrus. Delphinium. Acacia spp.*	0
01505167	ACADESINE	C9H14N4O5	258.23	glucose uptake stimulant; AMPK activator	synthetic	0
01503101	AZLOCILLIN SODIUM	C20H22N5NaO6 S	483.48	antibacterial	semisynthetic	0
01505200	BENAZEPRIL HYDROCHLORIDE	C24H29ClN2O5	460.96	ACE inhibitor. antihypertensive	synthetic	0
01505820	TOPOTECAN HYDROCHLORIDE	C23H24ClN3O5	457.91	antineoplastic; topoisomerase I inhibitor	semisynthetic	0
01500283	ESTRADIOL CYPIONATE	C26H36O3	396.57	estrogen	semisynthetic	0
01505753	GLYCOPYRROLATE	C19H28BrNO3	398.34	anticholinergic	synthetic; AHR-504	0

01505943	RABEPRAZOLE SODIUM	C18H20N3NaO3S	381.43	gastric acid secretion inhibitor	synthetic; LY-307640. E-3810	0
00200011	MUNDULONE	C26H26O6	434.49		*Mundulea sericea*	0
01503720	SELAMECTIN	C43H63NO11	769.98	antiparasitic. antimite	semisynthetic	0
01503111	CEFTRIAXONE SODIUM TRIHYDRATE	C18H22N8Na2O10S3	652.59	antibacterial	semisynthetic; RO-13-9904	0
00330086	DIBUTYL PHTHALATE	C16H22O4	278.35	plasticiser. suspect endocrine disruptor	synthetic	0
01500840	CHOLIC ACID	C24H40O5	408.58		mammalian bile	0
01500589	TRIAMTERENE	C12H11N7	253.26	diuretic	semisynthetic	0
00310012	HESPERETIN	C16H14O6	302.28		aglycone of hesperidin (00310011)	0
00200034	ATRANORIN	C19H18O8	374.35		*Common lichen metabolite*	0
01501105	PUROMYCIN HYDROCHLORIDE	C22H31Cl2N7O5	544.44	antineoplastic. antiprotozoal	*Streptomyces alboniger*	0
01500305	FLUOROURACIL	C4H3FN2O2	130.07	antineoplastic. pyrimidine antimetabolite	synthetic	0
01500215	CYCLOSERINE	C3H6N2O2	102.09	antibacterial (tuberculostatic)	*Streptomyces spp*	0
01500606	VALPROATE SODIUM	C8H15NaO2	166.19	anticonvulsant	synthetic	0
00200833	ACACETIN DIACETATE	C20H16O7	368.34		derivative of acacetin	0
00211539	ARABITOL(D)	C5H12O5	152.14		*Lecanora sordida & other lichen & fungi*	0
01505331	3.3'-DIINDOLYLMETHANE	C17H14N2	246.31	apoptosis inducer	synthetic	0
01505974	CLOFAZIMINE	C27H22Cl2N4	473.40	antibacterial. antilepretic. antituberculosis	synthetiNSC-141046c; G-30320	0
01502010	DIACERIN	C19H12O8	368.30	antiinflammatory	synthetic	0
00212151	CHLORQUINALDOL	C10H7Cl2NO	228.07	antiinfectant. antifungal	synthetic	0
00100105	8beta-HYDROXYCARAPIN. 3.8-HEMIACETAL	C27H32O8	484.55		derivative	0
01503982	AGMATINE SULFATE	C5H16N4O4S	228.27	NMDA blocker. alpha-2 adrenergic agonist; NO synthase inhibitor	*Ambrosia entemisifolia*	0
01500484	PHENYLPROPANOLAMINE HYDROCHLORIDE	C9H14ClNO	187.67	vasoconstrictor. decongestant. anorexic	synthetic	0
01500319	GRAMICIDIN	C60H92N12O10	1141.47	antibacterial	*Bacillus brevis*	0
01500673	MOLSIDOMINE	C9H14N4O4	242.23	antianginal	synthetic	0
01503214	EDOXUDINE	C11H16N2O5	256.26	antiviral	synthetic; EDU; EUDR; ORF-15817; RWJ-15817	0
00100291	STROPHANTHIDIN	C23H32O6	404.50	cardiotonic	*Strophanthus kombe*	0

01505411	BENZOXIQUINE	C16H11NO2	249.27	antiinfective	synthetic; NSC-3951	0
02300094	ASTEMIZOLE	C28H31FN4O	458.58	H1 antihistamine (nonsedating)	synthetic	0
01501200	XYLAZINE	C12H16N2S	220.33	analgesic	synthetic	0
01500828	CANRENOIC ACID. POTASSIUM SALT	C22H29KO4	396.57	aldosterone antagonist. diuretic	semisynthetic	0
01502103	ANTHRAQUINONE	C14H8O2	208.21	irritant	synthetic	0
01503073	ADIPHENINE HYDROCHLORIDE	C20H26ClNO2	347.88	muscle relaxant (smooth)	synthetic	0
01502012	FOSFOSAL	C7H7O6P	218.10	analgesic. antiinflammator y	synthetic	0
01505920	PANTETHINE	C22H42N4O8S2	554.73	antilipemic	semisynthetic	0
01506075	KAWAIN	C14H14O3	230.26		*Piper methysticum*	0
00330002	MITOMYCIN C	C15H18N4O5	334.33	antineoplastic	*Streptomyces verticillatus*	0
01500507	PROCYCLIDINE HYDROCHLORIDE	C19H30ClNO	323.91	anticholinergic	synthetic	0
01500497	PREDNISOLONE ACETATE	C23H30O6	402.49	glucocorticoid	semisynthetic	0
01500321	GUAIFENESIN	C10H14O4	198.22	expectorant	synthetic	0
01505429	FLUCYTOSINE	C4H4FN3O	129.09	antifungal	synthetic; RO-2-9915	0
00200441	XANTHOXYLIN	C10H12O4	196.20		*Xanthoxylium spp. Artemisia brevifolia*	0
02300219	EDROPHONIUM CHLORIDE	C10H16ClNO	201.69	acetylcholinester ase inhibitor	synthetic	0
01505983	PHENFORMIN HYDROCHLORIDE	C10H16ClN5	241.72	antidiabetic	synthetic	0
01500238	DICLOXACILLIN SODIUM	C19H16Cl2N3N aO5S	492.31	antibacterial	semisynthetic	0
01503142	TENOXICAM	C13H11N3O4S2	337.37	antiinflammator y	synthetic	0
01505622	RALOXIFENE HYDROCHLORIDE	C28H28ClNO4S	510.05	antiestrogen	synthetic-LY-156758	0
00300540	HYMECROMONE METHYL ETHER	C11H10O3	190.20		*Dalbergia volubilis. Eupatorium pauciflorum*	0
01504143	ARTENIMOL	C15H24O5	284.35	antimalarial. antiinflammator y	semisynthetic; dihydroartemi sinin	0
01500231	DEXAMETHASONE ACETATE	C24H31FO6	434.50	glucocorticoid. antiinflammator y	semisynthetic	0
01503229	METHAPYRILENE HYDROCHLORIDE	C14H20ClN3S	297.85	H1 antihistamine	synthetic	0
01500999	DROFENINE HYDROCHLORIDE	C20H32ClNO2	353.93	antispasmodic	synthetic	0
01502001	ACETYL-L-LEUCINE	C8H15NO3	173.21	antivertigo	synthetic	0
01400242	N-METHYLBENZYLAMINE HYDROCHLORIDE	C8H12ClN	157.64		Ephedra sp.	0
00202175	12a-HYDROXY-5-DEOXYDEHYDROMUNDUS ERONE	C19H18O6	342.35		derivative	0
01500280	ERYTHROMYCIN	C37H67NO13	733.94	antibacterial	*Streptomyces erythreus*	0

01500634	IPRONIAZID SULFATE	C9H15N3O5S	277.30	monoamine oxidase inhibitor. antidepressant	synthetic	0
01500595	TRIMETHOPRIM	C14H18N4O3	290.32	antibacterial	synthetic	0
01503265	NICLOSAMIDE	C13H8Cl2N2O4	327.12	anthelmintic. teniacide	synthetic; BAY-2353	0
02300259	NICOTINE DITARTRATE	C18H26N2O12	462.41	nicotinyl acetylchloline receptor agonist. ectoparasiticide	*Nicotiana tabacum*	0
01500944	CARNOSINE	C9H14N4O3	226.23		mammalian skeletal muscle	0
01505842	PIPERIC ACID	C12H10O4	218.21		*Piper nigrum. Minthostachys verticillata. Menta piperita*	0
01504229	METAXALONE	C12H15NO3	221.25	muscle relaxant (skeletal)	synthetic	0
01504231	CLARITHROMYCIN	C38H69NO13	747.97	antibacterial	*Streptomyces erythreus*	0
01300018	GLUTAMINE (L)	C5H10N2O3	146.14	dietary suppliment	beetroot; widely distributed in plants	0
00200070	LECANORIC ACID	C16H14O7	318.28		Common constituent of lichens	0
00100032	GEDUNIN	C28H34O7	482.57	antifeedant; heat shock inducer	numerous *Meliaceae species*	0
01505743	ALEURETIC ACID	C16H32O5	304.43		shellac constituent	0
01500665	ACEBUTOLOL HYDROCHLORIDE	C18H29ClN2O4	372.89	antihypertensive . antianginal. antiarrhythmic	synthetic	0
01504237	HYDROQUINONE	C6H6O2	110.11	antioxidant	synthetic	0
01501026	FENDILINE HYDROCHLORIDE	C23H26ClN	351.92	coronary vasodilator	synthetic	0
01501192	ESTRADIOL-3-SULFATE. SODIUM SALT	C18H23NaO5S	374.43	estrogen	semisynthetic	0
01504023	THERMOPSINE PERCHLORATE	C15H21ClN2O5	344.79		*Anabasis aphylla. Thermopsis spp*	0
01504130	3.7-DIHYDROXYFLAVONE	C15H10O4	254.24		*Platymiscium praecox*	0
01505278	3-HYDROXY-3'.4'-DIMETHOXYFLAVONE	C17H14O5	298.29		synthetic	0
01500598	TRIPROLIDINE HYDROCHLORIDE	C19H23ClN2	314.86	antihistaminic	synthetic	0
01500354	IPRATROPIUM BROMIDE	C20H30BrNO3	412.37	bronchodilator. antiarrhythmic	synthetic	0
00100359	3.16-DIDEOXYMEXICANOLIDE-3beta-DIOL	C27H36O7	472.58		derivative	0
01505802	GEMIFLOXACIN MESYLATE	C19H24FN5O7S	485.49	antibacterial	synthetic	0
01500691	PHENTOLAMINE HYDROCHLORIDE	C17H20ClN3O	317.82	antihypertensive	synthetic	0
01505710	ALISKIREN HEMIFUMARATE	C34H57N3O10	667.84	renin inhibitor	synthetic	0

01502047	LIOTHYRONINE (L- isomer) SODIUM	C15H11I3NNaO 4	672.96	thyroid hormone	synthetic; L-isomer	0
01501207	KINETIN RIBOSIDE	C15H17N5O5	347.33		semisynthetic	0
01505205	OLMESARTAN MEDOXOMIL	C29H30N6O6	558.59	Angiotensin II inhibitor prodrug. antihypertensive	synthetic	0
01503304	URETHANE	C3H7NO2	89.09	antineoplastic. cytotoxic	synthetic; NSC-746	0
01503237	HALCINONIDE	C24H32ClFO5	454.97	glucocorticoid. antiinflammator y	synthetic	0
01505976	DOXAZOSIN MESYLATE	C24H29N5O8S	547.59	antihypertensive	synthetic; UK-33274-27	0
00210925	METHYL ORSELLINATE	C9H10O4	182.17		lichens and lichen acids	0
01505864	3-ACETYLCOUMARIN	C11H8O3	188.18		synthetic	0
01504244	BETAMIPRON	C10H11NO3	193.20	sweetener	synthetic	0
01500480	PHENOLPHTHALEIN	C20H14O4	318.33	cathartic	synthetic	0
01500368	LINCOMYCIN HYDROCHLORIDE	C18H35ClN2O6 S	443.01	antibacterial	*Streptomyces lincolnensis. S espinosus. S variabilis*	0
01500333	HOMATROPINE METHYLBROMIDE	C17H24BrNO3	370.28	anticholinergic (opthalmic)	semisynthetic	0
01503279	OXETHAZAINE	C28H41N3O3	467.65	anesthetic (local)	synthetic	0
01505712	PREDNISOLONE SODIUM PHOSPHATE	C21H27Na2O8P	484.39	antiinflammator y. glucocorticoid	semisynthetic	0
01505678	TICARCILLIN DISODIUM	C15H14N2Na2O 6S2	428.39	antibacterial	synthetic	0
01506052	TROLOX	C14H18O4	250.29	antioxidant	synthetic	0
00200743	ERGOSTEROL	C28H44O	396.66		yeast	0
01502037	LOMEFLOXACIN HYDROCHLORIDE	C17H20ClF2N3 O3	387.81	antibacterial	synthetic	0
01500437	NORETHINDRONE	C20H26O2	298.42	progestogen	synthetic	0
01500545	SULFACETAMIDE	C8H10N2O3S	214.24	antibacterial	synthetic	0
01505704	BISOPROLOL FUMARATE	C22H35NO8	441.52	beta-blocker. antihypertensive	synthetic; CL-297939	0
01505994	ETHAMIVAN	C12H17NO3	223.27	CNS & respiratory stimulant	synthetic; NCS-406087	0
00300111	2-METHOXY-5 (6)EPOXY-TETRAHYDROCARYOPHYL LENE	C16H28O2	252.40		derivative	0
01502231	CELLOBIOSE (D[+])	C12H22O11	342.30		enzymatic hydrolysis of cellulose	0
01503104	BENDROFLUMETHIAZIDE	C15H14F3N3O4 S2	421.41	diuretic. antihypertensive	synthetic	0
01500130	ASPIRIN	C9H8O4	180.16	analgesic. antipyretic. antiinflammator y	synthetic	0
01500222	DAPSONE	C12H12N2O2S	248.30	antibacterial. leprostatic. dermatitis herpetiformis suppressant	synthetic	0
01501114	PIMETHIXENE MALEATE	C23H23NO4S	409.50	H1 antihistamine	synthetic; BP-400	0

01503807	ISOSORBIDE MONONITRATE	C6H9NO6	191.14	antianginal	semisynthetic; BM-22145. IS-5-MN. AHR-4698	0
00310018	QUINIC ACID	C7H12O6	192.17		*Cinchona spp*	0
01505306	ASPARTAME	C14H18N2O5	294.30	sweetener	synthetic	0
01500649	THEOBROMINE	C7H8N4O2	180.16	diuretic. bronchodilator. cardiotonic	*Camelia. Theobroma. Cola spp*	0
01503931	MORANTEL CITRATE	C18H24N2O7S	412.46	anthelmintic	synthetic	0
01503210	BETAMETHASONE 17.21-DIPROPIONATE	C28H37FO7	504.60	glucocorticoid. antiinflammatory	semisynthetic	0
01500602	TUBOCURARINE CHLORIDE	C37H41ClN2O6	645.20	muscle relaxant (skeletal)	*Chondodendron spp*	0
01500599	TROPICAMIDE	C17H20N2O2	284.36	anticholinergic (opthalmic)	synthetic	0
01503215	ENOXACIN	C15H17FN4O3	320.32	antibacterial	synthetic	0
01503801	NAPROXOL	C14H16O2	216.28	antinflammatory. analgesic. antipyretic	synthetic	0
01505474	CEFONICID SODIUM	C18H17N6NaO8S3	564.55	antibacterial	semisynthetic; SK&F-D-75073-Z	0
01504617	ISAXONINE	C7H11N3	137.18	nerve growth stimulant	synthetic	0
01500656	AJMALINE	C20H26N2O2	326.44	antiarrhythmic (Class Ia): inhibits glucose uptake by mitochondria. & PAF blocker	*Rauwolfia spp.. Melodinus balansae. Tonduzia longifolia*	0
00300021	GIBBERELLIC ACID	C19H22O6	346.38		*Gibberella fujikuroi*	0
01500444	NOVOBIOCIN SODIUM	C31H35N2NaO11	634.62	antibacterial	*Streptomyces niveus and S griseus*	0
01502042	CEFOPERAZONE SODIUM	C25H26N9NaO8S2	667.65	antibacterial	semisynthetic	0
01500482	PHENYLBUTAZONE	C19H20N2O2	308.38	antiinflammatory	synthetic	0
01505314	SARAFLOXACIN HYDROCHLORIDE	C20H18ClF2N3O3	421.83	antibacterial	synthetic	0
02300166	ALPRENOLOL	C15H24ClNO2	285.81	betaadrenergic blocker	synthetic	0
02300345	NIPECOTIC ACID	C6H11NO2	129.16	GABA uptake inhibitor	synthetic	0
00200484	DEOXYSAPPANONE B 7.4'-DIMETHYL ETHER	C18H18O5	314.34		*Caesalpinia sappan*	0
00100173	EPOXYGEDUNIN	C28H34O8	498.57		*Meliaceae spp*	0
01500839	CINCHONIDINE	C19H22N2O	294.39	antimalarial	*Cinchona spp*	0
01505701	FUMAZENIL	C15H14FN3O3	303.29	benzodiazepine antagonist	synthetic; Ro-15-1788/000	0
01503234	BETA-PROPIOLACTONE	C3H4O2	72.06	antiinfective	synthetic; NSC-21626	0
00310030	RHODINYL ACETATE	C12H22O2	198.30		common constituent of plant essential oils	0
01500717	6.4'-DIHYDROXYFLAVONE	C15H10O4	254.24	antihaemorrhagic	*Cassia spp* as glycoside	0

01505169	DESOXYPEGANINE HYDROCHLORIDE	C11H13ClN2	208.69	acetylcholinesterase inhibitor. antiParkinsonism	derivative of peganine *Peganum spp*	0
01504078	SENNOSIDE A	C42H38O20	862.75	cathartic	*Cassia & Rheum spp*	0
01500235	DIBENZOTHIOPHENE	C12H8S	184.26	keratolytic	synthetic	0
01500494	PRAZIQUANTEL	C19H24N2O2	312.41	anthelmintic	synthetic	0
01500322	GUANABENZ ACETATE	C10H12Cl2N4O2	291.13	antihypertensive	synthetic	0
01505692	PANCURONIUM BROMIDE	C35H60Br2N2O4	732.68	neuromuscular blocker	synthetic; ORG-NA-97	0
01505705	FLUDARABINE PHOSPHATE	C10H13FN5O7P	365.21	antineoplastic	synthetic;	0
01505772	BUCETIN	C12H17NO3	223.27	analgesic	synthetic	0
00100563	OXONITINE	C33H43NO12	645.71		derivative of aconitine	0
01500609	VIDARABINE	C10H13N5O4	267.24	antiviral	synthetic	0
01500193	CLINDAMYCIN HYDROCHLORIDE	C18H34Cl2N2O5S	461.45	antibacterial. inhibits protein synthesis	semisynthetic; U-21251	0
01504179	FEXOFENADINE HYDROCHLORIDE	C32H40ClNO4	538.13	nonsedating H1-antihistamine	synthetic	0
01504401	PIOGLITAZONE HYDROCHLORIDE	C19H21ClN2O3S	392.90	antidiabetic	synthetic	0
01501203	RETINOL	C20H30O	286.46	vitamin A	fish & liver oils. eggs. milk	0
00100432	DEOXYGEDUNIN	C28H34O6	466.57		*Meliaceae spp*	0
01503092	GLUCONOLACTONE	C6H10O6	178.14	chelating agent	synthetic	0
01500209	CRESOL	C7H8O	108.14	antiinfectant	coal tar	0
01503925	KETOROLAC TROMETHAMINE	C19H24N2O6	376.41	antiinflammatory	synthetic	0
01503078	ANIRACETAM	C12H13NO3	219.24	cognitive enhancer	synthetic	0
01502085	CYCLOCREATINE	C5H9N3O2	143.14	regulator of creatine biosynthesis	heart & muscle tissue	0
01500832	CARYLOPHYLLENE OXIDE	C14H22O	206.33		clove. cinnamon and many other oils	0
00240942	ARTHONIOIC ACID	C29H36O9	528.60		Arthonia impolita	0
01505195	EPITESTOSTERONE	C19H28O2	288.43	androgen	cooccurs with testosterone in mammals	0
01500330	HEXYLRESORCINOL	C12H18O2	194.27	anthelmintic. topical antiseptic	synthetic	0
01500579	TOBRAMYCIN	C18H37N5O9	467.52	antibacterial. inhibits protein synthesis	*Streptomyces spp*	0
01500607	VANCOMYCIN HYDROCHLORIDE	C67H77Cl3N8O24	1484.75	antibacterial	*Streptomyces orientalis*	0
01503422	SEMUSTINE	C10H18ClN3O2	247.72	antineoplastic	synthetic	0
01505726	DESONIDE	C24H32O6	416.51	antiinflammatory. glucocorticoid	semisynthetic; D-2083	0
00211066	2-METHOXYRESORCINOL	C7H8O3	140.14		*Peltophorum africanum*	0

01504009	LEVULINIC ACID. 3-BENZYLIDENYL-	C12H12O3	204.22		derivative	0
01300043	SORBITOL	C6H14O6	182.17	sweetening agent and humectant	fruits of Sorbus and *Crataegus spp*	0
01506073	IDAZOXAN HYDROCHLORIDE	C11H13ClN2O2	240.69	alpha2-adrenergic blocker	synthetic	0
01500543	STREPTOZOSIN	C8H15N3O7	265.22	antineoplastic. alkylating agent	synthetic	0
01505262	SERTRALINE HYDROCHLORIDE	C17H18Cl3N	342.69	antidepressant. 5HT uptake inhibitor	synthetic	0
00307123	ESTRONE BENZOATE	C25H26O3	374.48		semisynthetic	0
00107108	5alpha-ANDROSTAN-3.17-DIONE	C19H28O2	288.43	androgen	human urine & adrenal cortex	0
00100101	PRENYLETIN	C14H14O4	246.26		*Ptaeroxylon obliquum*	0
01502083	N- (9-FLUORENYLMETHOXYCARBONYL)-L-LEUCINE	C21H23NO4	353.42	antiinflammatory	synthetic; NPC-15199	0
01500353	IODOQUINOL	C9H5I2NO	396.95	antiamebic	synthetic	0
00107023	SITOSTERYL ACETATE	C31H52O2	456.75		widespread in plants	0
01500415	MINOXIDIL	C9H15N5O	209.25	antihypertensive. antialopecia agent	synthetic	0
01501021	FENSPIRIDE HYDROCHLORIDE	C15H21ClN2O2	296.79	antiinflammatory. bronchodilator	synthetic	0
01500678	gamma-AMINOBUTYRIC ACID	C4H9NO2	103.12	antihypertensive	widely distributed in higher plants	0
00100060	MEXICANOLIDE	C27H32O7	468.55		*Meliaceae spp*	0
00201364	DUARTIN. DIMETHYL ETHER	C20H24O6	360.41		derivative of DUARTIN (00201177)	0
01505252	PALMATINE	C21H24NO5	370.42	uterine contractant. antibacterial. antiarrhythmic. inotropic. adrenocorticotropic. anticholinesterase. analgesic	*Jateorhesa palmata. Berberis spp*	0
01503929	MEXILETINE HYDROCHLORIDE	C11H18ClNO	215.72	antiarrhythmic	synthetic; KO-1173	0
01505292	NONOXYNOL-9	C33H60O10	616.84	spermatocide. contraceptive	synthetic	0
01500734	3.4'-DIMETHOXYFLAVONE	C17H14O4	282.29		derivative *Millettia flowers*	0
01501000	ETHAVERINE HYDROCHLORIDE	C24H30ClNO4	431.96	antispasmodic	synthetic	0
00100650	DESACETYL (7)KHIVORINIC ACID. METHYL ESTER	C28H40O10	536.62		semisynthetic	0
00300553	PELLETIERINE HYDROCHLORIDE	C8H16ClNO	177.67		*Punica granatum*	0

01501116	MEBHYDROLIN NAPHTHALENESULFONATE	C29H28N2O6S2	564.68	H1 antihistamine	synthetic	0
00240736	2-METHOXYXANTHONE	C14H10O3	226.23		*Mammea and Keilmeyera spp.*	0
01500335	HYDROCHLOROTHIAZIDE	C7H8ClN3O4S2	297.74	diuretic	semisynthetic	0
01505681	TOLTRAZURIL	C18H14F3N3O4S	425.38	coccidiostat	synthetic; BAY-Vi-9142	0
01505435	GUANFACINE	C9H9Cl2N3O	246.09	antihypertensive	synthetic; BS-100-141	0
01500633	HEXETIDINE	C21H45N3	339.61	antifungal	synthetic	0
00307033	3-HYDROXY-4-(SUCCIN-2-YL)-CARYOLANE delta-LACTONE	C19H28O4	320.43		derivative of caryophyllene	0
00300423	DIFUCOL HEXAMETHYL ETHER	C18H22O6	334.37		derivative	0
01500168	CEPHRADINE	C16H19N3O4S	349.41	antibacterial	semisynthetic	0
01500544	SULFABENZAMIDE	C13H12N2O3S	276.31	antibacterial	synthetic	0
01500413	MICONAZOLE NITRATE	C18H15Cl4N3O4	479.14	antifungal (topical)	synthetic	0
01500308	FLURBIPROFEN	C15H13FO2	244.26	antiinflammatory. analgesic	synthetic	0
01503002	BENZYL BENZOATE	C14H12O2	212.25	scabicide	synthetic	0
00200035	GANGALEOIDIN	C18H14Cl2O7	413.21		*Lecanora gangaleoides*	0
00300013	PIMPINELLIN	C13H10O5	246.22	GABA receptor antagonist. phototoxin	Heracleum maximum root; mp 118-119 C	0
01505711	ACAMPROSATE CALCIUM	C10H20CaN2O8S2	400.48	alcohol antagonist	synthetic	0
01505470	CLINDAMYCIN PALMITATE HYDROCHLORIDE	C34H64Cl2N2O6S	699.86	antibacterial. inhibits protein synthesis	semisynthetic; U-25179E	0
01503705	ANETHOLE	C10H12O	148.20	expectorant. gastric stimulant. insecticide	anise. fennel and other plant oils	0
00100005	ANTHOTHECOL	C28H32O7	480.56		also as 11-ACETOXYCEDRELONE	0
00200010	HAEMATOXYLIN	C16H14O6	302.28		*Haemotoxylon campechianum*	0
01502073	IMIDAZOL-4-YLACETIC ACID SODIUM SALT	C5H5N2NaO2	148.09	GABAc antagonist	synthetic	0
01500355	ISONIAZID	C6H7N3O	137.14	antibacterial. tuberculostatic	synthetic	0
01503393	PIPOBROMAN	C10H16Br2N2O2	356.05	antineoplastic. alkylating agent	synthetic	0
01503679	AZITHROMYCIN	C38H72N2O12	749.01	antibacterial	semisynthetic	0
01505706	MUPIROCIN	C26H44O9	500.63	antibacterial. antimycoplasmal. isoleucyl-tRNA synthetase inhibitor	*Pseudomonas fluorescens; BRL-4910A*	0
01500261	DISOPYRAMIDE PHOSPHATE	C21H32N3O5P	437.48	antiarrhythmic	synthetic	0
01500251	DIMENHYDRINATE	C24H28ClN5O3	469.97	antiemetic	synthetic	0
01503607	THALIDOMIDE	C13H10N2O4	258.23	hypnotic	synthetic	0

01503107	BROMHEXINE HYDROCHLORIDE	C14H21Br2ClN2	412.59	expectorant	synthetic	0
01505391	MONTELUKAST SODIUM	C34H33ClNNaO3S	594.15	leucotriene antagonist. antiasthmatic	synthetic; MK-476	0
01500362	KETOCONAZOLE	C26H28Cl2N4O4	531.44	antifungal	synthetic	0
01503076	QUINAPRIL HYDROCHLORIDE	C25H31ClN2O5	474.98	antihypertensive. ACE inhibitor	synthetic	0
01505293	DIALLYL SULFIDE	C6H10S	114.21	anitbacterial. antifungal. antineoplastic. antihypercholesterolaemic. hepatoprotectant	*Allium spp. Wasabia japonica*	0
01500746	NARINGENIN	C15H12O5	272.25	antiulcer. gibberellin antagonist	widely distributed in plants	0
00210186	CITRININ	C13H14O5	250.25	antibacterial	*Penicillium citrinum*	0
01500998	ETHOXYQUIN	C14H19NO	217.31	antioxidant	synthetic	0
01501201	TOLAZAMIDE	C14H21N3O3S	311.40	antidiabetic	synthetic	0
01504190	AVOBENZONE	C20H22O3	310.39	sunscreen	synthetic	0
01504172	CITALOPRAM	C20H21FN2O	324.40	antidepressant. 5HT reuptake inhibitor	synthetic	0
02300206	DIAZOXIDE	C8H7ClN2O2S	230.67	antihypertensive. diuretic. activates K channels and AMPA receptors	synthetic; SCH-6783; NSC-64198	0
01505434	HALOTHANE	C2HBrClF3	197.38	anesthetic	synthesis	0
00300547	PHLORIDZIN	C21H24O10	436.41	induces experimental glucosuria. antifeedant	*Rosaceae spp*	0
01504174	BUPROPION	C13H19Cl2NO	276.20	antidepressant	synthetic	0
01505652	ARSANILIC ACID	C6H8AsNO3	217.05	antibacterial	synthetic; AS-101	0
01506043	SOTALOL HYDROCHLORIDE	C12H21ClN2O3S	308.82	beta-adrenergic agonist	synthetic; MJ-1999	0
01500618	ACRIFLAVINIUM HYDROCHLORIDE	C14H14ClN3	259.74	antiinfective. intercalating agent	synthetic	0
01505310	REBAMIPIDE	C19H15ClN2O4	370.79	antiulcer. antioxidant	synthetic	0
01500877	KYNURAMINE	C9H12N2O	164.20		urine of various animals	0
01503136	THIAMPHENICOL	C12H15Cl2NO5S	356.22	antibacterial	synthetic	0
01504260	LEVOFLOXACIN	C18H20FN3O4	361.37	antibacterial	synthetic	0
00100528	PEUCENIN	C15H16O4	260.29		*Peucedanum ostruthium. Ptaeroxylon obliquum*	0
01503711	ORBIFLOXACIN	C19H20F3N3O3	395.38	antibacterial	synthetic	0
01503207	CYCLOBENZAPRINE HYDROCHLORIDE	C20H22ClN	311.85	muscle relaxant (skeletal)	synthetic	0

00300133	CLOVANEDIOL DIACETATE	C19H30O4	322.44		*derivative Dipterocarpus pilosus. Salvia canariensis. Viguiera oaxacana. Sindora sumatrana*	0
00240914	PSEUDO-ANISATIN	C15H22O6	298.33	GABA antagonist	*Illicium anisatum*	0
01505123	21-ACETOXYPREGNENOLONE	C23H34O4	374.52	precursor in corticoid biosynthesis. derivative	semisynthetic	0
01500605	URSODIOL	C24H40O4	392.58	anticholelithogenic; LD50(rat) 890 mg/kg ip	bear bile	0
01505420	DEXPANTHENOL	C9H19NO4	205.25	cholinergic	semisynthetic	0
01501125	3-AMINOPROPANESULPHONIC ACID	C3H9NO3S	139.17	antibacterial; GABA agonist	synthetic	0
01501139	PRAMOXINE HYDROCHLORIDE	C17H28ClNO3	329.87	anesthetic (topical)	synthetic	0
01504269	LITHIUM CITRATE	C6H5Li3O7	209.91	antidepressant	synthetic	0
01505715	PANTOTHENIC ACID(d) Na salt	C9H16NNaO5	241.22	vitamin B5	rice bran. queen bee jelly	0
01505740	LIPOAMIDE	C8H15NOS2	205.34	hepatoprotectant	semisynthetic; thioctic acid amide	0
00100520	LUNARINE	C25H31N3O4	437.54		*Lunaria spp*	0
01505320	INDOLE-3-CARBINOL	C9H9NO	147.17	antineoplastic	*Brassica spp*	0
01505366	RANOLAZINE	C24H33N3O4	427.54	antianginal. antiischemic	synthetic; RS-43285-003; CVT-303	0
01500139	BENZOCAINE	C9H11NO2	165.19	anesthetic (topical)	synthetic	0
01501170	ACETAMINOSALOL	C15H13NO4	271.27	analgesic. antipyretic	synthetic	0
00200243	GRISEOFULVIC ACID	C16H15ClO6	338.74		semisynthetic	0
01501197	PRIMULETIN	C15H10O3	238.24		*Primula spp*	0
01505908	MANGOSTIN TRIMETHYL ETHER	C27H32O6	452.55		derivative	0
00107013	SMILAGENIN	C27H44O3	416.64		*Smilax spp.*	0
01505610	LAMOTRIGINE	C9H7Cl2N5	256.09	anticonvulsant	synthetic; BW-430C	0
01504171	VENLAFAXINE	C17H27NO2	277.41	antidepressant	synthetic	0
00100303	ALLOPREGNANOLONE	C21H34O2	318.50		semisynthetic	0
01500397	METHOCARBAMOL	C11H15NO5	241.24	muscle relaxant (skeletal)	synthetic	0
01500341	HYDROFLUMETHIAZIDE	C8H8F3N3O4S2	331.29	antihypertensive. diuretic	synthetic	0
01503221	ETHISTERONE	C21H28O2	312.45	progestogen	synthetic; NSC-9565	0
01502024	PIPEMIDIC ACID	C14H17N5O3	303.32	antibacterial	synthetic	0
00201078	VIOLASTYRENE	C17H18O3	270.33		*Dalbergia miscolobium*	0
01506004	LACTOBIONIC ACID	C12H22O12	358.30	food additive	semisynthetic	0

01502005	CARBENOXOLONE SODIUM	C34H48Na2O7	614.73	antiinflammatory. antisecretory. antiulcer	semisynthetic	0
01500155	CAFFEINE	C8H10N4O2	194.19	CNS stimulant	*Cofea. Theobroma. Camelia and Cola spp*	0
01504243	OXCARBAZEPINE	C15H12N2O2	252.27	antipsychotic	synthetic	0
01505178	ROPINIROLE	C16H24N2O	260.38	dopamine receptor agonist. antiParkinsonian	synthetic; SK&F-101468	0
01503934	PERPHENAZINE	C21H26ClN3OS	403.97	antipsychotic	synthetic	0
01503273	HYDROCORTISONE BUTYRATE	C25H36O6	432.56	glucocorticoid. antiinflammatory	semisynthetic	0
01503611	PENTOXIFYLLINE	C13H18N4O3	278.31	PDE inhibitor. bronchodilator. vasodilator	synthetic	0
00300542	SANTONIN	C15H18O3	246.30		*Artemisia spp.*	0
00240944	HAEMATOXYLIN PENTAACETATE	C26H24O12	528.47		derivative	0
01503983	BATYL ALCOHOL	C21H44O3	344.58		fish oils	0
01501143	SULFAPHENAZOLE	C15H14N4O2S	314.36	antibacterial	synthetic	0
01502195	PIRACETAM	C6H10N2O2	142.15	antinauseant	synthetic	0
01500300	FLUMETHAZONE PIVALATE	C27H36F2O6	494.58	glucocorticoid. antiinflammatory	semisynthetic	0
01500248	DIHYDROERGOTAMINE MESYLATE	C34H41N5O8S	679.79	vasoconstrictor. antimigraine	semisynthetic	0
01500288	ETHAMBUTOL HYDROCHLORIDE	C10H26Cl2N2O2	277.23	antibacterial (tuberculostatic)	synthetic	0
01500496	PREDNISOLONE	C21H28O5	360.45	glucocorticoid	semisynthetic	0
01505530	DEXCHLORPHENIRAMINE MALEATE	C20H23ClN2O4	390.87	antihistamine	synthetic	0
01504272	GATIFLOXACIN	C19H22FN3O4	375.40	antibacterial	synthetic; BMS-206584-01. AM-1155	0
01502084	PROADIFEN HYDROCHLORIDE	C23H32ClNO2	389.96	cytochrome P450 inhibitor. Ca antagonist	synthetic; SKF-525A	0
01800031	URSOLIC ACID	C30H48O3	456.71	diuretic. antineoplastic. antiulcer	*Rhododendron spp. Epigaea asiatica. surface wax of fruits*	0
00231043	1.3.5-TRIMETHOXYBENZENE	C9H12O3	168.19	spasmolytic	synthetic	0
00200215	2-METHYL-5.7.8-TRIMETHOXYISOFLAVONE	C19H18O5	326.35		derivative	0
01505347	RIBOFLAVIN	C17H20N4O6	376.37	Vitamin B2; Vitamin cofactor; LD50(rat) 560 mg/kg ip	retina. whey and urine	0
01500360	KANAMYCIN A SULFATE	C18H38N4O15S	582.58	antibacterial	*Streptomyces kanamyceticus*	0
01505172	ACARBOSE	C25H43NO18	645.61	alpha-glucosidase & saccharase inhibitor. antidiabetes.	*Actinoplanes spp*	0

				antihyperlipidaemia. antiobesity		
01300099	CYSTINE	C6H12N2O4S2	240.30	nutrient	widespread in plants and animals	0
00270043	3beta-HYDROXY-23.24-BISNORCHOL-5-ENIC ACID	C22H34O3	346.51		semisynthetic	0
00100162	KHIVORIN	C32H42O10	586.68		Khaya and other West African timbers	0
00100254	5beta-12-METHOXY-4.4-BISNOR-8.11.13-PODOCARPATRIEN-3-ONE	C16H20O2	244.33		semisynthetic	0
01502229	ABIETIC ACID	C20H30O2	302.46		common diterpene acid in conifers	0
01503042	ARTEMISININ	C15H22O5	282.33	antimalarial	*Artemisia annua*	0
01501017	FERULIC ACID	C10H10O4	194.18	antineoplastic. choleretic. food preservative	widely distributed in plants	0
01500576	THIOTHIXENE	C23H29N3O2S2	443.63	antipsychotic	synthetic	0
01503419	NAFRONYL OXALATE	C26H35NO7	473.57	vasodilator	synthetic	0
01503250	MEPHENTERMINE SULFATE	C11H19NO4S	261.34	vasoconstrictor	synthetic	0
01505155	3-HYDROXYTYRAMINE	C8H11NO2	153.18	dopaminergic	synthetic	0
00310009	EPIANDROSTERONE	C19H30O2	290.44		in normal human urine	0
01505031	GARDENIN B	C19H18O7	358.35		*Gardenia lucida; Brickellia. Citrus and Mentha spp*	0
00201466	MANDELIC ACID. METHYL ESTER	C9H10O3	166.17		free acid found in Poria spp.	0
01504232	2.3-DIHYDROXY-6.7-DICHLOROQUINOXALINE	C8H4Cl2N2O2	231.03	NMDA receptor antagonist (gly)	synthetic	0
01500350	INDOMETHACIN	C19H16ClNO4	357.79	antiinflammatory. antipyretic. analgesic	synthetic	0
01500184	CHLORPROMAZINE	C17H19ClN2S	318.87	antiemetic. antipsychotic	synthetic	0
01505003	PIZOTYLINE MALATE	C23H27NO5S	429.53	5HT antagonist. antimigraine	synthetic; PIZOTIFEN; BC-105	0
01505722	DESOXYMETASONE	C22H29FO4	376.47	antiinflammatory	semisynthetic; HOE-304. R-2113. A-41-304	0
00270029	PRASTERONE ACETATE	C21H30O3	330.47	adrenocortical hormone. antidepressant	semisynthetic	0
00200015	ISOROTENONE	C23H22O6	394.42		semisynthetic	0
01503977	LOVASTATIN	C24H36O5	404.55	antihyperlipidemic. HMGCoA reductase inhibitor	*Aspergillus spp; mevinolin*	0
01500992	FLUMEQUINE	C14H12FNO3	261.25	antibacterial	synthetic	0
01504216	ZOXAZOLAMINE	C7H5ClN2O	168.58	muscle relaxant. antirrheumatic	synthetic	0

01505202	AMLODIPINE BESYLATE	C26H31ClN2O8S	567.06	Ca channel blocker	synthetic	0
02300165	AMIODARONE HYDROCHLORIDE	C25H30ClI2NO3	681.78	adrenergic agonist. coronary vasodilator. Ca channel blocker	synthetic	0
01500736	3.6-DIMETHOXYFLAVONE	C17H14O4	282.29		semisynthetic	0
01506053	TYLOXAPOL	C70H124O13	1173.76	polymeric nonionic detergent	synthetic	0
01504504	CARSALAM	C8H5NO3	163.13	analgesic	synthetic	0
00100455	MEROGEDUNIN	C21H28O4	344.45		derivative	0
00300146	VULPINIC ACID	C19H14O5	322.32	antiinflammatory. antibacterial. plant growth inhibitor	numerous lichens. e.g. *Letharia vulpina*	0
01505475	RHIZOCARPIC ACID	C28H23NO6	469.49		*Rhizocarpon spp. Calicium hyperelium; mp 178 C*	0
01505018	ASTRAGALOSIDE IV	C41H68O14	784.99		*Astragalus species*	0
01500666	ACEMETACIN	C21H18ClNO6	415.83	antiinflammatory	synthetic	0
01500564	TETRACAINE HYDROCHLORIDE	C15H25ClN2O2	300.83	anesthetic (local)	synthetic	0
01500292	ETHIONAMIDE	C8H10N2S	166.24	antibacterial. tuberculostatic	*synthetic*	0
01400156	ISOPEONOL	C9H10O3	166.17		*Paeonia spp*	0
01506091	MENAQUINONE-4	C21H24O2	308.42	antioxidant. alkaline phosphatase enhancer	synthetic; VITAMIN K2	0
01500857	BILIRUBIN	C33H36N4O6	584.67		pigment mamalian gallstones. blood and urine	0
01503431	CYTIDINE	C9H13N3O5	243.22		nucleoside	0
01500986	GITOXIN	C41H64O14	780.95	cardiotonic	*Digitalis spp.*	0
01505816	PREGABALIN	C8H17NO2	159.23	anticonvulsant	synthetic; CI-1008	0
01500578	TIMOLOL MALEATE	C17H28N4O7S	432.49	betaadrenergic blocker	synthetic	0
01500525	RACEPHEDRINE HYDROCHLORIDE	C10H16ClNO	201.69	bronchodilator. decongestant	synthetic	0
01505445	METHYLATROPINE NITRATE	C18H26N2O6	366.41	anticholinergic	semisynthetic	0
01504142	ACETRIAZOIC ACID	C9H6I3NO3	556.86	X-ray contrast medium	synthetic	0
01503080	AMBROXOL HYDROCHLORIDE	C13H19Br2ClN2O	414.57	expectorant	synthetic	0
00270078	5alpha-CHOLESTANOL	C27H48O	388.68		eggs. faces. gall stones. adrenal tissue	0
01505725	BETAMETHAZONE SODIUM PHOSPHATE	C22H28FNa2O8P	516.41	antiinflammatory. glucocorticoid	semisynthetic	0
01505005	L-DEOXYALLIIN	C6H11NO2S	161.22	antineoplastic	*Allium spp*	0
00202130	D-PERSEITOL	C7H16O7	212.20		*Persia spp.*	0

01505896	GRAMINE	C11H14N2	174.24		*Arundo. Hordeum. Phalaris spp*	0
01500860	S-ISOCORYDINE (+)	C20H23NO4	341.41	sedative. cholinergic	*Dicentra canadensis. Artabotyris suaveoleus and Cordyalis species*	0
01505921	PANGAMIC ACID SODIUM	C10H18NNaO8	303.24		vitamin B15	0
01500989	18alpha-GLYCYRRHETINIC ACID	C30H46O4	470.69	antiinflammator y	epimer of aglycone *Glycyrrhiza glabra*	0
01500410	METOCLOPRAMIDE HYDROCHLORIDE	C14H23Cl2N3O 2	336.26	antiemetic	synthetic	0
01500441	NORGESTREL	C21H28O2	312.45	progestogen	synthetic	0
01500212	CYCLOPENTOLATE HYDROCHLORIDE	C17H26ClNO3	327.85	mydriatic	synthetic	0
01504188	PIRENPERONE	C23H24FN3O2	393.46	5HT2 receptor antagonist	synthetic	0
01505425	ACETOHEXAMIDE	C15H20N2O4S	324.40	antidiabetic	synthetic; 33006	0
01505590	ANAGRELIDE HYDROCHLORIDE	C10H8Cl3N3O	292.55	antithrombotic	synthetic; BL-4162a	0
02300218	EFAROXAN HYDROCHLORIDE	C13H17ClN2O	252.74	insulin secretagogue. alpha2 adrenorecetor antagonist	synthetic	0
01505899	HYDROQUININE HYDROBROMIDE HYDRATE	C20H29BrN2O3	425.36	depigmentor	semisynthetic; derivatice of quinine	0
00201181	IRIGENIN. DIBENZYL ETHER	C32H28O8	540.57		derivative of Irigenin	0
01500438	NORETHINDRONE ACETATE	C22H28O3	340.46	Oral contraceptive (in combination with estrogen)	synthetic	0
01505437	LEVOCARNITINE	C7H15NO3	161.20	cofactor for fatty acid metabolism. replenisher in arterial disease	semisynthetic	0
01505656	PANTHENOL	C9H19NO4	205.25	vitamin B5 precursor. radioprotectant	various plants	0
01505690	PIPAMPERONE	C21H30FN3O2	375.49	antipsychotic	synthetic; R-3345	0
02300229	GLYBURIDE	C23H28ClN3O5 S	494.01	antihyperglyce mic	synthetic	0
01503219	DIOSMIN	C28H32O15	608.55	vascular protectant	*Zanthoxylum avicennae*	0
00200208	ASARYLALDEHYDE	C10H12O4	196.20	fly attractant	*Daucus carota. Acorus calamus. Asarum europaeum*	0
01505326	SOLANESYL ACETATE	C47H76O2	673.12		*Nicotiana tabacum; Murraya exotica; Pinus spp*	0

01506067	ENILCONAZOLE	C14H14Cl2N2O	297.18	antifungal	synthetic; R-23979	0
01500129	APOMORPHINE HYDROCHLORIDE	C17H18ClNO2	303.79	emetic	synthetic	0
01500511	PROPANTHELINE BROMIDE	C23H30BrNO3	448.40	anticholinergic	synthetic	0
01502197	PIPERIDOLATE HYDROCHLORIDE	C21H26ClNO2	359.89	antispasmodic	synthetic	0
00200141	2-ETHOXYCARBONYL-2-ETHOXYOXALOYLOXYDIHYDROCHRYSIN DIMETHYL ETHER	C24H24O9	456.45		mp 111-114 C	0
00300007	EUPARIN	C13H12O3	216.23		*Eupatorium macculatum*	0
00240828	3.4-DIMETHOXYDALBERGIONE	C17H16O4	284.31	induces dermatitis	*Dalbergia spp. Machaerium spp*	0
01502128	CYCLOLEUCINE	C6H11NO2	129.16	NMDA receptor antagonist (gly)	synthetic	0
00100541	DICTAMNINE	C12H9NO2	199.21		*Dictamnus albus and other Rutaceae*	0
01504200	ISOLIQUIRITIGENIN	C15H12O4	256.26	aldose reductase inhibitor. antineoplastic. antiinflammatory	widespread in Fabaceae	0
02300228	KAINIC ACID	C10H15NO4	213.23	glutamate receptor agonist. anthelmintic	*Digenia simplex*	0
00310002	ADONITOL	C5H12O5	152.14		Adonis spp	0
01503127	DEQUALINIUM CHLORIDE	C30H40Cl2N4	527.58	antiinfectant	synthetic; BAQD-10	0
01505156	N-PHENYLANTHRANILIC ACID	C13H11NO2	213.23	ion channel (Cl) blocker	synthetic	0
01500120	AMOXICILLIN	C16H19N3O5S	365.41	antibacterial	semisynthetic	0
01500677	ALBUTEROL (+/-)	C13H21NO3	239.31	bronchodilator. tocolytic	synthetic	0
01500314	GENTAMICIN SULFATE	C21H45N5O11S	575.68	antibacterial	*Micromonospora spp*	0
01504303	MOXIFLOXACIN HYDROCHLORIDE	C23H29ClFN3O4	465.95	antibacterial	synthetic	0
01501179	ESTRADIOL PROPIONATE	C21H28O3	328.45	estrogen	semisynthetic	0
01505870	4-HYDROXYANTIPYRINE	C11H12N2O2	204.23	analgesic	synthetic	0
01505788	NIFUROXAZIDE	C12H9N3O5	275.22	antiseptic	synthetic	0
01505955	COLISTIN SULFATE	C52H102N16O21S2	1351.61	antibacterial	*Bacillus polymyxa colistinus; polymyxin E*	0
01600300	CREATININE	C4H7N3O	113.11	metabolic enhancer	metabolite in muscle and urine; renal metabolite	0
01503639	RAUWOLSCINE HYDROCHLORIDE	C21H27ClN2O3	390.91	alpha2 adrenergic antagonist	*Rauwolfia. Aspidosperma and Vinca spp*	0
01504001	CAFESTOL	C20H28O3	316.44		coffee bean oil	0
01505915	PROXYPHYLLINE	C10H14N4O3	238.24	PDE inhibitor. bronchodilator. vasodilator	synthetic	0
01505774	OXELAIDIN CITRATE	C26H41NO10	527.61	antitussive	synthetic	0

01500119	AMODIAQUINE DIHYDROCHLORIDE	C20H24Cl3N3O	428.79	antimalarial	synthetic	0
01501109	PROBUCOL	C31H48O2S2	516.85	antihyperlipidemic	synthetic	0
01500613	WARFARIN	C19H16O4	308.33	anticoagulant. rodenticide	synthetic	0
01505201	FAMCICLOVIR	C14H19N5O4	321.33	antiviral	synthetic	0
01505723	BETAMETHASONE ACETATE	C24H31FO6	434.50	antiinflammatory	semisynthetic	0
00100358	1.7-DIDEACETOXY-1.7-DIOXO-3-DEACETYLKHIVORIN	C26H32O7	456.54		*Meliaceae spp*	0
00270088	CHOLESTAN-3-ONE	C27H46O	386.66		minor mammalian sterol	0
01505484	LUPEOL	C30H50O	426.73	antineoplastic	widespread in plants	0
01504024	LAGOCHILIN	C20H36O5	356.50		*Lagochilus inebrians*	0
00100325	DIGITONIN	C56H92O29	1229.34		*Digitalis purpurea*	0
01501184	ESTRADIOL ACETATE	C20H26O3	314.42	estrogen	semisynthetic	0
01500424	NAPHAZOLINE HYDROCHLORIDE	C14H15ClN2	246.74	adrenergic agonist. nasal decongestant	synthetic	0
01500812	BEKANAMYCIN SULFATE	C18H39N5O14S	581.60	antibacterial	semisynthetic; *Streptomyces kanamyceticus; NK-1006*	0
01503267	NOMIFENSINE MALEATE	C20H22N2O4	354.40	antidepressant. dopamine uptake inhibitor	synthetic	0
01505907	METHYLPHENIDATE HYDROCHLORIDE	C14H20ClNO2	269.77	CNS stimulant	synthetic	0
01503630	SPAGLUMIC ACID	C11H16N2O8	304.25	neurotransmitter; mGluR3 receptors	brain tissue	0
00300005	SPHONDIN	C12H8O4	216.19		*Heracleum* maximum root; mp 191-192 C	0
01504185	TETRANDRINE	C38H42N2O6	622.76	analgesic. antineoplastic. antihypertensive. lymphotoxin	*Triclisia and Cyclea spp*	0
01400666	METHOXYVONE	C17H14O3	266.29	anabolic	synthetic	0
01501119	PROGLUMIDE	C18H26N2O4	334.41	anticholinergic	synthetic	0
01501108	MEFEXAMIDE	C15H25ClN2O3	316.83	stimulant (central)	synthetic	0
01500801	ALLANTOIN	C4H6N4O3	158.11	wound healing agent	allantoic fluid; widely distributed in plants	0
01500434	NITROFURAZONE	C6H6N4O4	198.13	antiinfective (topical)	synthetic	0
01503262	NALTREXONE HYDROCHLORIDE	C20H23NO4	341.41	morphine antagonist	synthetic	0
01505466	TRIMETHADIONE	C6H9NO3	143.14	anticonvulsant	synthetic	0
01503420	QUIPAZINE MALEATE	C17H19N3O4	329.35	antidepressant. oxytocic	synthetic	0

01506077	METICRANE	C10H13NO4S2	275.34	diuretic. antihypertensive	synthetic; SD-17102	0
01505985	NIZATIDINE	C12H21N5O2S2	331.46	antiulcer. H2 antagonist	synthetic; LY-139037	0
00310019	CHRYSANTHEMIC ACID. ETHYL ESTER	C12H20O2	196.29	insecticide	pyrethrum flowers	0
00201595	OSAJIN	C25H24O5	404.46		*Maclura pomifera*	0
01505476	HELICIN	C13H16O7	284.26		Spiraea spp	0
01500108	ALLOPURINOL	C5H4N4O	136.11	antihyperuricemia. antigout. antiurolithic	synthetic	0
01500211	CYCLIZINE	C18H22N2	266.38	H1 antihistamine	synthetic	0
01500230	DEXAMETHASONE	C22H29FO5	392.47	glucocorticoid	semisynthetic	0
01505346	KETANSERIN TARTRATE	C22H22FN3O3	395.43	5HT2/5HT1C seritonin antagonist	synthetic; R41468	0
01503908	PACLITAXEL	C47H51NO14	853.92	antineoplastic	*Taxus breviofolia*	0
01505113	FLUNIXIN MEGLUMINE	C21H28F3N3O7	491.46	analgesic. antiinflammatory	synthetic; SCH-14714	0
01503084	AMRINONE	C10H9N3O	187.20	cardiac stimulant	synthetic	0
01502015	NIFLUMIC ACID	C13H9F3N2O2	282.22	analgesic. antiinflammatory	synthetic; UP-83	0
01300028	MANNITOL	C6H14O6	182.17	diuretic. sweetener. diagnostic aid	manna and seaweeds; exudates of olive and plane trees	0
01505234	AVOCADYNE	C17H32O3	284.44	antibacterial. antifungal	*Persea spp*	0
01503254	6alpha-METHYLPREDNISOLONE ACETATE	C24H32O6	416.51	glucocorticoid	semisynthetic	0
01505672	VINCRISTINE SULFATE	C46H58N4O14S	923.05	antineoplastic	*Vinca rosea; 37231. NSC-67574*	0
01500616	ACETARSOL	C8H10AsNO5	275.09	antiprotozoal; diethylamine salt as antiphilitic	synthetic	0
01503086	TIAPRIDE HYDROCHLORIDE	C15H25ClN2O4S	364.89	neuroleptic	synthetic	0
01502260	ABAMECTIN	C48H72O14	873.10	antiparisitic (avermectin A1a shown)	*Streptomyces avermitilis*	0
01503423	SPIRAMYCIN	C43H74N2O14	843.07	antibacterial	*Streptomyces ambofaciens*	0
01500195	CLOFIBRIC ACID	C10H11ClO3	214.65	antihyperlipoproteinemic	synthetic	0
01500281	ERYTHROMYCIN STEARATE	C55H103NO15	1018.43	antibacterial	*Streptomyces erythreus*	0
00300055	CADIN-4-EN-10-OL	C15H26O	222.37		*Chamaecyparis spp and Juniperus spp.*	0
01501210	HUMULENE (alpha)	C15H24	204.35		hops and clove oils	0
01505398	PHENYLETHYL ALCOHOL	C8H10O	122.16	antimicrobial	synthetic	0

01503722	ATORVASTATIN CALCIUM	C33H33CaFNO5	582.71	antihyperlipidemic. HMGCoA reductase inhibitor	synthetic	0
01500440	NORFLOXACIN	C16H18FN3O3	319.33	antibacterial	synthetic	0
01505364	CEFPROZIL	C18H19N3O5S	389.43	antibacterial	semisynthetic; BMY-28100-03-800	0
01500304	FLUOROMETHOLONE	C22H29FO4	376.47	glucocorticoid. antiinflammatory	semisynthetic	0
01500667	ADENOSINE PHOSPHATE	C10H14N5O7P	347.22	vasodilator. neuromodulator	widespread in living tissue	0
01500358	ISOSORBIDE DINITRATE	C6H8N2O8	236.13	antianginal	semisynthetic	0
01505448	NITHIAMIDE	C5H5N3O3S	187.17	antibacterial	synthetic; CI-5279	0
01501182	ESTRADIOL BENZOATE	C25H28O3	376.50	estrogen	semisynthetic	0
01502053	ALRESTATIN	C14H9NO4	255.23	aldose reductase inhibitor	synthetic; AY-22284A	0
01505779	DICHLORISONE ACETATE	C23H28Cl2O5	455.38	antipruretic	semisynthetic; R-25788	0
01600654	CHAULMOSULFONE	C48H76N2O4S	777.21	antilepretic	synthetic	0
01504021	LUPININE	C10H19NO	169.26	antifeedant. antiinflammatory. oxytoxic	*Anabasis aphylla. Lupinus spp*	0
01401406	DERRUSNIN	C19H16O7	356.33		*Derris robusta*	0
00100599	2-METHYL GRAMINE	C12H16N2	188.27		derivative	0
01500848	LITHOCHOL-11-ENIC ACID	C24H38O3	374.56		*Curvularia spp.*	0
01500107	ADENOSINE	C10H13N5O4	267.24	antiarrhythmic. cardiac depressant	widespread in living tissue	0
01503428	CHOLINE CHLORIDE	C5H14ClNO	139.62	choleretic. lipotropic. hepatoprotectant	widespread in the plant and animal kingdoms	0
01504144	DIRITHROMYCIN	C42H78N2O14	835.09	antibacterial	semisynthetic	0
01500676	OUABAIN	C29H44O12	584.66	antiarrhythmic. cardiotonic. hypertensive. Na/K ATPase inhibitor	*Acokanthera and Strophanthus spp*	0
01500220	DANAZOL	C22H27NO2	337.46	anterior pituitary suppressant	synthetic	0
01503968	TACROLIMUS	C44H69NO12	804.04	immune suppressant. antifungal	*Streptomyces tsukubaensis*	0
01504273	MIGLITOL	C8H17NO5	207.22	alpha-glucosidase inhibitor. antidiabetic	synthetic; BAYm-1099	0
02300242	LOXAPINE SUCCINATE	C22H24ClN3O5	445.90	antipsychotic	synthetic	0
01505447	OCTODRINE	C8H19N	129.24	vasoconstrictor. anesthetic (local)	synthetic; SK&F-51	0
02300292	MIANSERIN HYDROCHLORIDE	C18H21ClN2	300.83	5HT antagonist	synthetic	0
00100287	TOTAROL	C20H30O	286.46		*Podocarpus spp. Dacrydium cupressinum. Tetraclinis*	0

					articulata and Thujopsis dolabrata	
01503922	TRYPTAMINE	C10H12N2	160.22	psychotropic	*Acacia spp. Lens esculenta. Prosopsis juliflora and fungi Ponaeolus foenisicii and Coprinus micaceus*	0
01501135	NICARDIPINE HYDROCHLORIDE	C26H30ClN3O6	515.99	vasodilator	synthetic	0
01500491	PIROXICAM	C15H13N3O4S	331.35	antiinflammator y	synthetic	0
01500313	GEMFIBROZIL	C15H22O3	250.34	antihyperlipopr oteinemic	synthetic	0
01500810	AMINACRINE	C13H10N2	194.23	local antiseptic	synthetic	0
01503429	CLOFIBRATE	C12H15ClO3	242.70	antihyperlipide mic	synthetic	0
01504501	beta-NAPHTHOL	C10H8O	144.17	anthelmintic. antiseptic	synthetic	0
01501199	ZAPRINAST	C13H13N5O2	271.28	cGMP phosphodiestera se inhibitor	synthetic; M&B-22948	0
01505488	SALIDROSIDE	C15H22O7	314.33		*Salix triandra. Rhodiola spp*	0
01500340	HYDROCORTISONE PHOSPHATE TRIETHYLAMINE	C33H61N2O8P	644.83	glucocorticoid	semisynthetic	0
01500356	ISOPROPAMIDE IODIDE	C23H33IN2O	480.43	anticholinergic	synthetic	0
01505413	BENZOYLPAS	C14H11NO4	257.24	antibacterial. tuberculostatic	synthetic	0
01505463	TAURINE	C2H7NO3S	125.14	neuroprotectant. inhibitory neurotransmitte r. GABA agonist	widespread in animal tissue	0
01505492	METHYSERGIDE MALEATE	C25H31N3O6	469.54	vasoconstrictor. antimigraine	semisynthetic	0
01503085	PYRITHYLDIONE	C9H13NO2	167.20	hypnotic. sedative	synthetic	0
01505786	TRICLABENDAZOLE	C14H9Cl3N2OS	359.66	anthelmintic	synthetic	0
01503222	FIPEXIDE HYDROCHLORIDE	C20H22Cl2N2O 4	425.31	psychostimulant	synthetic	0
01505122	AZTREONAM	C13H17N5O8S2	435.43	antibiotic	semisynthetic; SQ-26776	0
01500641	PENTAMIDINE ISETHIONATE	C23H36N4O10S 2	592.69	antiprotozoal. inhibits nucleic acid & protein synthesis	synthetic; MB-800. RP-2512	0
01600075	QUERCETIN PENTAMETHYL ETHER	C20H20O7	372.37		derivative	0
01505884	ESEROLINE FUMARATE	C17H22N2O5	334.37	μ-opioid receptor agonist. analgesic	*Coynanthe yohimbe*	0
01502162	p-CHLOROPHENYLALANINE	C9H10ClNO2	199.63	Irreversible inhibitor of tryptophan hydroxylase	synthetic	0
01500472	PHENACEMIDE	C9H10N2O2	178.19	anticonvulsant	synthetic	0
01500135	BACLOFEN	C10H12ClNO2	213.66	muscle relaxant (skeletal)	synthetic	0

01502028	CEPHALEXIN	C16H17N3O4S	347.39	antibacterial	semisynthetic	0
01500600	TRYPTOPHAN	C11H12N2O2	204.23	antidepressant. nutrient; LD50(rat) 1634 mg/kg ip	many plants. animal protein	0
01500585	TRIACETIN	C9H14O6	218.20	antifungal (topical)	synthetic	0
01505497	SOLIFENACIN SUCCINATE	C27H32N2O6	480.56	muscarinic M3 antagonist	synthetic; YM-905. YM-67905	0
01504505	CARZENIDE	C7H7NO4S	201.20	diuretic. carbonic anhydrase inhibitor	synthetic	0
01505995	BUTYL PARABEN	C11H14O3	194.23	antifungal. preservative	synthetic	0
00201177	DUARTIN (-)	C18H20O6	332.35		*Dalbergia variabilis*	0
00300160	3-PINANONE OXIME	C10H17NO	167.25		derivative	0
00100609	XANTHYLETIN	C14H12O3	228.24		*Brosimum rubescens. Ruta. Boenninghausenia. Flindersia. Zanthoxylum. and Luvunga spp.*	0
01505255	HUPERZINE A	C15H18N2O	242.32	anticholinesterase. cognition enhancer	*Lycopodium spp*	0
01500855	CITRULLINE	C6H13N3O3	175.18		*Citrullis vulgaris and the alga Grateloupia filicina*	0
01503954	TULOBUTEROL	C12H19Cl2NO	264.19	bronchodilator. beta adrenergic agonist	synthetic	0
01501194	TOLPERISONE HYDROCHLORIDE	C16H24ClNO	281.82	muscle relaxant (skeletal)	synthetic; N-553	0
00200331	SALSALATE	C14H10O5	258.23	analgesic	synthetic	0
01504184	AMINOLEVULINIC ACID HYDROCHLORIDE	C5H10ClNO3	167.59	antineoplastic	synthetic	0
01500400	METHOXSALEN	C12H8O4	216.19	antipsoriatic. pigmentation agent	amni visnaga	0
01500225	SODIUM DEHYDROCHOLATE	C24H33NaO5	424.51	choleretic	semisynthetic	0
01500548	SULFAMETHAZINE	C12H14N4O2S	278.33	antibacterial	synthetic	0
01500536	SISOMICIN SULFATE	C19H39N5O11S	545.61	antibacterial. binds to ribosomes	*Micromonospora myoensis*	0
01502230	ASCORBIC ACID	C6H8O6	176.12	antiscorbutic. antiviral	Vitamin C	0
01502258	MONENSIN SODIUM (monensin A is shown)	C37H63NaO10	690.89	antibacterial	*Streptomyces cinnamonensis*	0
00100434	DIHYDRODEOXYGEDUNIN	C28H36O6	468.59		*Meliaceae spp*	0
00300556	CHRYSAROBIN	C15H12O3	240.26		*Andira araroba* (glacial AcOH)	0
01502114	p-FLUOROPHENYLALANINE	C9H10FNO2	183.18	amino acid antagonist. protein	synthetic	0

				synthesis inhibitor		
01500430	NIACIN	C6H5NO2	123.11	antihyperlipidemic. vitamin (enzyme cofactor)	widespread in the plant and fungal kingdom	0
01500171	CHLORAMBUCIL	C14H19Cl2NO2	304.21	antineoplastic. alkylating agent	synthetic	0
01502026	TRANEXAMIC ACID	C8H15NO2	157.21	hemostatic	synthetic	0
01500477	PHENINDIONE	C15H10O2	222.24	anticoagulant	synthetic	0
01504218	ACRISORCIN	C25H28N2O2	388.51	antifungal	synthetic	0
01500233	DEXTROMETHORPHAN HYDROBROMIDE	C18H26BrNO	352.31	antitussive	synthetic	0
01500267	DOXYLAMINE SUCCINATE	C21H28N2O5	388.46	antihistaminic. hypnotic	synthetic	0
01505309	BIFONAZOLE	C22H18N2	310.40	antifungal. calmodulin antagonist	synthetic	0
01502255	SALICIN	C13H18O7	286.28	analgesic. antipyretic	*Salix spp*	0
01505480	IFOSFAMIDE	C7H15Cl2N2O2P	261.08	antineoplastic	synthetic; MJF-9325. Z-4942. NSC-109724	0
01504187	TELENZEPINE HYDROCHLORIDE	C19H23ClN4O2S	406.93	antiulcer. M1 muscarinic antagonist	synthetic	0
01503917	CLENBUTEROL HYDROCHLORIDE	C12H19Cl3N2O	313.65	bronchodilator. beta2 adrenergic agonist	synthetic; NAB-365	0
01506065	CLORGILINE HYDROCHLORIDE	C13H16Cl3NO	308.63	MAO-A inhibitor. antidepressant. antiparkinsonian	synthetic	0
01500833	CANAVANINE	C5H12N4O3	176.17	NO synthase inhibitor	*Canavalia ensiformis*	0
00100612	TOTAROL-19-CARBOXYLIC ACID. METHYL ESTER	C21H30O3	330.47		*Podocarpus spp*	0
00100310	HECOGENIN ACETATE	C29H44O5	472.67		*Hechtia texensis. Agave & Yucca spp*	0
01500264	DOXEPIN HYDROCHLORIDE	C19H22ClNO	315.84	antidepressant	synthetic	0
01500646	SULFANILAMIDE	C6H8N2O2S	172.20	antibacterial	synthetic	0
01505354	FIPRONIL	C12H4Cl2F6N4OS	437.15	GABA Cl channel agonist. antiparasitic	synthetic; RM-1601. MB-46030	0
01503257	MIDODRINE HYDROCHLORIDE	C12H19ClN2O4	290.74	antihypertensive. vasoconstrictor	synthetic	0
00330009	SODIUM FLUOROACETATE	C2H2FNaO2	100.02	inhibits citric acid cycle	synthetic	0
00100566	DEOXYCHOLIC ACID	C24H40O4	392.58		bile constituent	0
00100031	FISSINOLIDE	C29H36O8	512.60		Cedrela fissilis. Khaya grandifoliola	0
01501003	FAMOTIDINE	C8H15N7O2S3	337.44	H2 antihistamine	*synthetic*	0
01500523	QUINIDINE GLUCONATE	C26H36N2O9	520.58	antiarrhythmic. antimalarial	*Cinchona spp*	0

01500253	DIMETHADIONE	C5H7NO3	129.11	anticonvulsant	*synthetic*	0
01500567	TETRAHYDROZOLINE HYDROCHLORIDE	C13H17ClN2	236.74	adrenergic agonist. nasal decongestant	*synthetic*	0
01502112	CYCLOHEXIMIDE	C15H23NO4	281.35	protein synthesis inhibitor	*synthetic*	0
01503252	METHAZOLAMIDE	C5H8N4O3S2	236.27	carbonic anhydrase inhibitor	*synthetic*	0
01505450	PREDNISOLONE HEMISUCCINATE	C25H32O8	460.52	antiinflammatory. glucocorticoid	*semisynthetic*	0
01300048	XYLOSE	C5H10O5	150.13	diagnostic aid	*many woods. straw. corncobs. cottonseed hulls and pecan shells.*	0
01505827	BECLAMIDE	C10H12ClNO	197.66	anticonvulsant. antiepileptic	synthetic; mp 94 deg C	0
00300119	2-HYDROXY-5 (6)EPOXY-TETRAHYDROCARYOPHYLLENE	C15H26O2	238.37		derivative	0
00300110	3-NOR-3-OXOPANASINSAN-6-OL	C14H22O2	222.33		derivative Panax ginseng	0
00200139	ISOTECTORIGENIN. 7-METHYL ETHER	C18H16O6	328.32		Dalbergia spp	0
00310021	PHYTOL	C19H38O	282.51		nettles	0
01502209	PHENETHYL CAFFEATE (CAPE)	C17H16O4	284.31	antineoplastic. antiinflammatory. immunomodulator. NFkB blocker	synthetic	0
01500113	POTASSIUM p-AMINOBENZOATE	C7H6KNO2	175.23	ultraviolet screen	*synthetic*	0
01900005	THIOPENTAL SODIUM	C11H17N2NaO2S	264.32	anesthetic	*synthetic*	0
01501156	SULFAMETHOXYPYRIDAZINE	C11H12N4O3S	280.30	antibacterial	*synthetic*	0
01500453	OXYMETAZOLINE HYDROCHLORIDE	C16H25ClN2O	296.84	adrenergic agonist. nasal decongestant	*synthetic*	0
01500344	HYDROXYUREA	CH4N2O2	76.05	antineoplastic. inhibits ribonucleoside diphosphate reductase	*synthetic*	0
01501002	DROPERIDOL	C22H22FN3O2	379.43	neuroleptic	*synthetic*	0
01505124	CLAVULANATE LITHIUM	C8H10LiNO5	207.11	beta-lactamase inhibitor	*semisynthetic*	0
01505166	ALENDRONATE SODIUM	C4H19NNaO10P2	326.13	bone resorption inhibitor. farnesyldiphosphate synthetase inhibitor. antimetastatic	*synthesis*	0
01504257	CARVEDILOL	C24H26N2O4	406.48	beta-adrenergic blocker	*synthetic*	0
01505460	RIMANTADINE HYDROCHLORIDE	C12H22ClN	215.76	antiviral	*synthetic; EXP-126*	0

00100568	SARMENTOSIDE B	C34H48O13	664.75		Strophanthus sarmentosis; mp 193-195	0
00300558	GEDUNOL	C28H36O7	484.59		Meliaceae spp	0
00200115	PECTOLINARIN	C29H34O15	622.58		Cirsium and Linaria spp. Kickxia elatine. Duranta plumieri	0
01505006	DIHYDROMYRISTICIN	C11H14O3	194.23	GSH transferase inducer	derivative; myristicin	0
01500869	MIMOSINE	C8H10N2O4	198.17	depilatory agent	Mimosa and Leucena spp	0
00212061	PYROCATECHUIC ACID	C7H6O4	154.12	antioxidant	Erythraea centaurium. Gentiana lutea	0
01500447	ORPHENADRINE CITRATE	C24H31NO8	461.51	muscle relaxant (skeletal). antihistaminic	*synthetic*	0
01501171	SACCHARIN	C7H5NO3S	183.18	sweetener	*synthetic*	0
01500128	ANTIPYRINE	C11H12N2O	188.23	analgesic	*synthetic*	0
01505281	ZOLMITRIPTAN	C16H21N3O2	287.36	antimigraine. 5HT[1B/1D] agonist	*synthetic*	0
01500272	EMETINE	C29H42Cl2N2O 4	553.57	inhibits RNA. DNA and protein synthesis	*Uragoga ipecacuanha*	0
01500663	YOHIMBINE HYDROCHLORIDE	C21H27ClN2O3	390.91	alpha adrenergic blocker. mydriatic. antidepressant	*Corynanthe spp*	0
01501007	FENOTEROL HYDROBROMIDE	C17H22BrNO4	384.27	betaadrenergic agonist	*synthetic*	0
01500584	TRANYLCYPROMINE SULFATE	C9H13NO4S	231.27	antidepressant. MAO inhibitor	*synthetic*	0
01503014	AKLOMIDE	C7H5ClN2O3	200.58	antiprotozoal. coccidiostat	*synthetic*	0
01503134	MENTHOL(-)	C10H20O	156.27	analgesic (topical). antipruritic agent	*Mentha piperita and other Mentha spp*	0
01503007	BETAINE HYDROCHLORIDE	C5H12ClNO2	153.60	antiarteriosclero tic. hypolipaemic. hepatoprotectan t	*widespread in fungi and plants*	0
01503231	NIMESULIDE	C13H12N2O5S	308.31	antiinflammator y	*synthetic; R-805*	0
02300104	TACRINE HYDROCHLORIDE	C13H15ClN2	234.73	anticholinesteras e. cognitive adjuvant. K channel blocker	synthetic; CL-970	0
01505981	ORNIDAZOLE	C7H10ClN3O3	219.62	antiinfective	synthetic; 16773-42-5	0
00200499	ACACETIN	C16H12O5	284.27	antiinflammator y. spasmolytic agent. antioxidant	Robinia pseudoacacia	0
00100550	OLEANOIC ACID	C30H48O3	456.71		leaves of Olea europea and	0

					Viscum album L.	
00100006	BUSSEIN	C43H54O18	858.89		Entandrophragma species	0
00100315	TIGOGENIN	C27H44O3	416.64		Agavaceae. Dioscoreaceae. Solanaceae. Scrophulariaceae. Liliaceae genera	0
01501212	LINALOOL (+)	C10H18O	154.25		Mentha arvensis and related essential oils	0
01504005	TRIPTOPHENOLIDE	C20H24O3	312.41		Tripterygium wilfordii	0
01504223	FENBUTYRAMIDE	C10H13NO	163.22	antihyperlipidemic	synthetic	0
01501217	LISINOPRIL	C21H31N3O5	405.49	ACE inhibitor	*synthetic*	0
00305025	PHENYL AMINOSALICYLATE	C13H11NO3	229.23	antibacterial (tuberculostatic)	*synthetic; NSC-40144*	0
01500178	CHLOROCRESOL	C7H7ClO	142.58	antiinfectant	*synthetic*	0
01500401	METHSCOPOLAMINE BROMIDE	C18H24BrNO4	398.30	anticholinergic	*semisynthetic*	0
01500339	HYDROCORTISONE HEMISUCCINATE	C25H34O8	462.54	glucocorticoid	*semisynthetic*	0
01503330	TETROQUINONE	C6H4O6	172.09	keratolytic	*synthetic*	0
01505975	BENZYDAMINE HYDROCHLORIDE	C19H24ClN3O	345.87	analgesic. antipyretic. antiinflammatory	*synthetic; AF-864*	0
01504520	EPIESTRIOL	C18H24O3	288.39	estrogen	semisynthetic	0
01505958	CLONAZEPAM	C15H10ClN3O3	315.71	anticonvulsant. sedative	synthetic	0
01503973	2-THIOURACIL	C4H4N2OS	128.15	thyroid depressant	synthetic	0
00201227	NONIC ACID	C9H16O4	188.22		hydroysis product of actinonin	0
01500706	ARCAINE SULFATE	C6H18N6O4S	270.31		Arca noae	0
01503802	AZADIRACHTIN	C35H44O16	720.73	antifeedant. insecticide	Melia azadirach and Azadirachta indica	0
01505846	11alpha-HYDROXYPROGESTERONE HEMISUCCINATE	C25H34O6	430.54	glucocorticoid	semisynthetic	0
01501187	FLUNISOLIDE	C24H31FO6	434.50	antiinflammatory	*semisynthetic*	0
01503978	HYDROXYCHLOROQUINE SULFATE	C18H28ClN3O5S	433.95	antimalarial. lupus suppressant	*synthetic*	0
01500152	BUSULFAN	C6H14O6S2	246.30	antineoplastic. alkylating agent	*synthetic*	0
01500442	NORTRIPTYLINE	C19H21N	263.38	antidepressant	*synthetic*	0
01500227	DESIPRAMINE HYDROCHLORIDE	C18H23ClN2	302.85	antidepressant	*synthetic*	0
01500551	SULFAPYRIDINE	C11H11N3O2S	249.29	antibacterial	*synthetic*	0
01500282	ESTRADIOL	C18H24O2	272.39	estrogen	*pregnancy urine*	0

01503240	PYRIDOSTIGMINE BROMIDE	C9H13BrN2O2	261.12	cholinergic	*synthetic*	0
01505426	ETHOXZOLAMIDE	C9H10N2O3S2	258.31	carbonic anhydrase inhibitor. antiulcer. antiglaucoma	*synthetic*	0
02300214	DILTIAZEM HYDROCHLORIDE	C22H27ClN2O4 S	450.98	Ca channel blocker. coronary vasodilator	synthetic	0
01504512	DEFERIPRONE	C7H9NO2	139.15	iron chelating agent	synthetic	0
01500679	AMINOPTERIN	C19H20N8O5	440.42	antineoplastic. antirheumatic. folic acid antagonist	synthetic; NSC-739	0
00100024	DIHYDROGEDUNIN	C28H36O7	484.59		Meliaceae spp.	0
01500733	ALPINETIN METHYL ETHER	C17H16O4	284.31		Eucalyptus spp	0
00310028	QUASSIN	C22H28O6	388.46	insecticide. antiamoebic	Quassia amara. Picrasma excelsa and Ailanthus glandulosa	0
00100286	PODOTOTARIN	C40H58O2	570.90		Podocarpus spp	0
00107022	beta-SITOSTEROL	C29H50O	414.72		widespread in plants	0
01500445	NYLIDRIN HYDROCHLORIDE	C19H26ClNO2	335.87	vasodilator (peripheral)	*synthetic*	0
01500462	PARGYLINE HYDROCHLORIDE	C11H14ClN	195.69	antihypertensive	*synthetic*	0
01505691	PENFLURIDOL	C28H27ClF5NO	523.97	antipsychotic	*synthetic; McN-JR-16341. R-16341*	0
01505438	HYDROCORTISONE VALERATE	C26H38O6	446.58	antiinflammatory. glucocorticoid	*semisynthetic*	0
02300270	PINACIDIL	C13H19N5	245.32	K channel agonist. antihypertensive	*synthetic*	0
01505270	PROPRANOLOL HYDROCHLORIDE (+/-)	C16H22ClNO2	295.81	antihypertensive. antianginal. antiarrhythmic	*synthetic*	0
01500518	PYRAZINAMIDE	C5H5N3O	123.11	antibacterial. tuberculostatic	*synthetic*	0
01500546	SULFADIAZINE	C10H10N4O2S	250.28	antibacterial	*synthetic*	0
01505483	DOXORUBICIN	C27H29NO11	543.53	antineoplastic	*semisynthetic*	0
01505125	ALCLOMETAZONE DIPROPIONATE	C28H37ClO7	521.05	antiinflammatory. glucocorticoid	*semisynthetic; SCH-22219*	0
02300348	1-(2-METHOXYPHENYL)PIERAZINE HYDROCHLORIDE	C11H17ClN2O	228.72	5HT1 receptor agonist	synthetic	0
01503044	CHROMOCARB	C10H6O4	190.15	vascular protectant	synthetic	0
01501004	DROPROPIZINE	C13H20N2O2	236.31	antitussive	synthetic; UCB-1967	0
01505485	PHYTONADIONE	C31H46O2	450.71	vitamin	semisynthetic	0
01600537	CATECHIN PENTAACETATE	C25H24O11	500.46		derivative	0

01500879	KYNURENINE	C10H12N2O3	208.21		mamalian urine	0
00100552	beta-AMYRIN ACETATE	C32H52O2	468.77		latof various species of rubber tree	0
01503051	RETINYL ACETATE	C22H32O2	328.49	vitamin precursor	semisynthetic	0
01504124	LINAMARIN	C10H17NO6	247.25		Linum usitatissimum. Manihot utilissimus	0
01505766	SYRINGIC ACID	C9H10O5	198.17		Glycine max; widespread in plants	0
01504155	APHYLLIC ACID	C15H26N2O2	266.38		*Anabasis aphylla*	0
01505382	5-HYDROXY-2'.4'.7.8-TETRAMETHOXYFLAVONE	C19H18O7	358.35		*Citrus spp. Limnophilia rugosa*	0
01505814	METFORMIN HYDROCHLORIDE	C4H12ClN5	165.62	antidiabetic	synthetic; LA-6023	0
01500161	CARBINOXAMINE MALEATE	C20H23ClN2O5	406.86	antihistaminic	synthetic	0
01500124	AMPROLIUM	C14H20Cl2N4	315.24	coccidiostat	synthetic	0
01500104	ACETYLCHOLINE	C7H16ClNO2	181.66	antiarrhythmic. miotic. vasodilator (peripheral)	synthetic	0
01500994	FLUPHENAZINE HYDROCHLORIDE	C22H28Cl2F3N3OS	510.45	H1 antihistamine	synthetic	0
01500541	STREPTOMYCIN SULFATE	C21H41N7O16S	679.66	antibacterial (tuberculostatic)	Streptomyces griseus	0
01502198	ANISINDIONE	C16H12O3	252.27	anticoagulant	synthetic	0
01505173	BAMBUTEROL HYDROCHLORIDE	C18H30ClN3O5	403.90	beta adrenergic agonist. bronchodilator. cholinesterase inhibitor	synthetic	0
00310041	VISNAGIN	C13H10O4	230.22		*Ammi visnaga*	0
01500737	3.7-DIMETHOXYFLAVONE	C17H14O4	282.29		*Pongamia pinnata*	0
00100267	TOTAROL ACETATE	C22H32O2	328.49		*Podocarpus totara*	0
00202178	EPIAFZELECHIN (2R.3R)(-)	C15H14O5	274.27		*Larix sibirica. Actinidia chinensis. Juniperus communis. Cassia javanica*	0
01504412	PODOPHYLLIN ACETATE	C24H24O9	456.45		derivative	0
01505274	3.4-DIHYDROXYCARANE	C10H18O2	170.25		monoterpene	0
00100652	DIHYDROXY (3alpha.12alpha)PREGNAN-20-ONE	C21H34O3	334.50		semisynthetic	0
00100360	beta-AMYRIN	C30H50O	426.73		widespread in plants	0
01501022	FARNESOL	C15H26O	222.37		major component in oil of *Hibiscus abelmoschus*	0
01500880	TRIGONELLINE	C7H7NO2	137.13	antihyperglycemic	*Trigonella foenumgraecum*	0

					and in coffee beans	
01502101	PERILLIC ACID (-)	C10H14O2	166.22	inhibits posttranslational cys isoprenylation. blocks G-protein	*Salvia dorisiana*	0
01505883	alpha-ERGOCRYPTINE	C32H41N5O5	575.71		ergot	0
01400151	ETHYL PARABEN	C9H10O3	166.17	antifungal	synthetic	0
01500114	AMINOCAPROIC ACID	C6H13NO2	131.17	hemostatic	synthetic	0
01500772	IODIPAMIDE	C12H11I3N2O4	627.94	radioopaque agent	synthetic; L-isomer: spectrum	0
01500530	ROXARSONE	C6H6AsNO6	263.03	antibacterial	synthetic	0
01500520	PYRIMETHAMINE	C12H13ClN4	248.71	antimalarial	synthetic	0
01500684	CIMETIDINE	C10H16N6S	252.34	antiulcer	synthetic	0
01500533	SODIUM SALICYLATE	C7H5NaO3	160.10	keratolytic	synthetic	0
01500642	PHENACETIN	C10H13NO2	179.22	analgesic. antipyretic	synthetic	0
01500357	ISOPROTERENOL HYDROCHLORIDE	C11H18ClNO3	247.72	bronchodilator	synthetic	0
01500255	DIOXYBENZONE	C14H12O4	244.24	ultraviolet screen	synthetic	0
01505244	CITICOLINE	C14H26N4O11P2	488.33	cognition enhancer. phosphocholine cytidyltransferase activator	cytidine-5'-diphosphocholine	0
01505760	OCTISALATE	C15H22O3	250.34	sunscreen	synthetic	0
01300044	TARTARIC ACID	C4H6O6	150.08	pharmaceutical aid	common plant constituent	0
01503416	MIZORIBINE	C9H13N3O6	259.22	immunosuppressant	synthetic	0
01500987	GLUTAMINE (D)	C5H10N2O3	146.14		widely distributed in plants	0
01503636	N-METHYL-D-ASPARTIC ACID (NMDA)	C5H9NO4	147.13	NMDA agonist	synthetic	0
01505130	3.4-DIMETHOXYCINNAMIC ACID	C11H12O4	208.21		*Piper methysticum. Veronica virginica*	0
01503970	METHOXYAMINE HYDROCHLORIDE	CH6ClNO	83.51	hydroxymethyltransferase inhibitor	synthetic	0
01505328	4'-DEMETHYLEPIPODOPHYLLOTOXIN	C21H20O8	400.38	antineoplastic	semisynthetic	0
01505041	BISPHENOL A	C15H16O2	228.29	endocrine disruptor. plastic monomer	synthetic	0
01501011	FENOPROFEN	C15H14O3	242.27	antiinflammatory	synthetic	0
01500327	HETACILLIN POTASSIUM	C19H22KN3O4S	427.57	antibacterial	semisynthetic	0
01500993	FLUNARIZINE HYDROCHLORIDE	C26H28Cl2F2N2	477.42	vasodilator	synthetic	0
01500237	DICLOFENAC SODIUM	C14H10Cl2NNaO2	318.13	antiinflammatory	synthetic	0
01500249	DIHYDROSTREPTOMYCIN SULFATE	C21H43N7O16S	681.67	antibacterial. tuberculostatic	*Streptomyces humidus*	0

01500592	TRIHEXYPHENIDYL HYDROCHLORIDE	C20H32ClNO	337.93	anticholinergic. antiparkinsonia n	synthetic	0
01501005	ETODOLAC	C17H21NO3	287.36	antiinflammator y	synthetic	0
01500295	EUCATROPINE HYDROCHLORIDE	C17H26ClNO3	327.85	anticholinergic (opthalmic)	semisynthetic	0
01500323	GUANETHIDINE SULFATE	C10H24N4O4S	296.39	antihypertensive . mitotic agent	synthetic	0
01505650	AMINOPENTAMIDE	C19H26N2O5S	394.49	antispasmodic. antiemetic	synthetic; NND-1962	0
01505537	ETIDRONATE DISODIUM	C2H6Na2O7P2	249.99	bone resorption inhibitor	synthetic	0
01500645	PREGNENOLONE	C21H32O2	316.48	glucocortcoid. antiinflammator y	semisynthetic	0
01504070	PHYSCION	C16H12O5	284.27	antibacterial. cathartic	*Xanthoria lichens. Rumex spp and various Aspergillus spp.*	0
01504616	MEPIROXOL	C6H7NO2	125.12	antihyperlipemi c	synthetic	0
00300384	5.7.4'-TRIMETHOXYFLAVONE	C18H16O5	312.32		*Cassia siamia. Citrus reticulata*	0
01504256	EPICATECHIN PENTAACETATE	C25H24O11	500.46		derivative	0
00300012	ISOPIMPINELLIN	C13H10O5	246.22		*Hercaleum maximum; mp 150-153 C dimorphic (142-145 C)*	0
00300047	ANISODAMINE HYDROBROMIDE	C17H23NO4	305.37	anticholinergic. antispasmodic	*Scopolia tanguticus*	0
01504025	SALSOLINE	C11H15NO2	193.24	antihypertensive . antihistamine	*Salsola richteri*	0
01500167	CEPHAPIRIN SODIUM	C17H16N3NaO6 S2	445.45	antibacterial	semisynthetic	0
00300618	SALICYLANILIDE	C13H11NO2	213.23	antipyretic. fungicide	synthetic	0
01500575	THIORIDAZINE HYDROCHLORIDE	C21H27ClN2S2	407.04	antipsychotic	synthetic	0
01500505	PROCHLORPERAZINE EDISYLATE	C22H30ClN3O6 S3	564.14	antiemetic. antipsychotic. treatment of vertigo	synthetic	0
01500581	TOLBUTAMIDE	C12H18N2O3S	270.35	antidiabetic	synthetic	0
01503412	ETANIDAZOLE	C7H10N4O4	214.18	antineoplastic. hypoxic cell radiosensitizer	synthetic	0
01505115	CLORSULON	C8H8Cl3N3O4S 2	380.65	antiparasitic. fasciolicide	synthetic; MK-401	0
01504073	UVAOL	C30H50O2	442.73	antineoplastic	*Arctostaphylos spp. Leucothoe keiskei. Crataegus cuneata. Osmanthus fragrans. Ilex latifolia*	0
00300104	2-METHYLENE-5-(2.5-DIOXOTETRAHYDROFURA N-3-YL)-6-OXO--10.10-	C18H24O4	304.38		derivative	0

	DIMETHYLBICYCLO[7: 2: 0]UNDECANE					
01503053	PIPENZOLATE BROMIDE	C22H28BrNO3	434.37	spasmolytic	synthetic	0
01505082	CYCLANDELATE	C17H24O3	276.37	vasodilator	synthetic; BS-572	0
01502102	QUINOLINIC ACID	C7H5NO4	167.12	differentiates between cerebral and forebrain NMDA receptors	synthetic	0
01505430	FLUROTHYL	C4H4F6O	182.06	central stimulant. convulsant	synthetic; SK&F-6539	0
01600759	4-HYDROXY-6-METHYLPYRAN-2-ONE	C6H6O3	126.11		*Pennicillium stipitatum*	0
00100009	CEDRELONE	C26H30O5	422.52		*Cedrela species*	0
00500123	CYCLOVERATRYLENE	C27H30O6	450.53		*Halopythus spp*	0
00203010	beta-TOXICAROL	C23H22O7	410.42		*Derris species & isomeration of alpha-toxicarol*	0
01501107	PICROTOXININ	C15H16O6	292.29	convulsant. GABA receptor antagonist. ichthyotoxin	*Anamirta cocculus. Menispermum cocculus*	0
00201697	EUPHOL	C30H50O	426.73		*Euphorbia spp.*	0
01500710	ACETYLPHENYLALANINE	C11H13NO3	207.23		synthetic	0
01500133	AZATHIOPRINE	C9H7N7O2S	277.26	immunosuppressant. antineoplastic. antirheumatic	synthetic	0
01501196	FLUMETHASONE	C22H28F2O5	410.46	antiinflammatory	semisynthetic	0
01500473	PHENAZOPYRIDINE HYDROCHLORIDE	C11H12ClN5	249.70	analgesic	synthetic	0
01500476	PHENELZINE SULFATE	C8H14N2O4S	234.27	antidepressant	synthetic	0
02300241	LOPERAMIDE HYDROCHLORIDE	C29H34Cl2N2O2	513.51	Ca channel blocker	synthetic	0
01500682	CAPTOPRIL	C9H15NO3S	217.28	antihypertensive	synthetic	0
01500277	ERGONOVINE MALEATE	C23H27N3O6	441.48	oxytocic. 5HT antagonist	*ergot and Convolvulvaceae spp*	0
01500580	TOLAZOLINE HYDROCHLORIDE	C10H13ClN2	196.68	adrenergic blocker	synthetic	0
01503243	LABETALOL HYDROCHLORIDE	C19H25ClN2O3	364.87	adrenergic blocker	synthetic	0
01503263	CYCLOTHIAZIDE	C14H16ClN3O4S2	389.88	diuretic	synthetic	0
01503270	PRILOCAINE HYDROCHLORIDE	C13H21ClN2O	256.77	anesthetic (local)	synthetic	0
01503255	METHYLPREDNISOLONE SODIUM SUCCINATE	C26H33NaO8	496.53	glucocorticoid. antiinflammatory	semisynthetic	0
01900004	CHLORDIAZEPOXIDE	C16H14ClN3O	299.76	minor tranquilzer. sedative	synthetic	0
00107113	ANDROSTERONE ACETATE	C21H32O3	332.48	androgen	semisynthetic	0
01505810	PEONIFLORIN	C23H30O11	482.48	antiinflammatory.	*Peonia spp*	0

				antispasmodic. antihypertensive . antidiuretic		
00200846	APIGENIN	C15H10O5	270.24	antispasmodic. antineoplastic. topoisomerase I inhibitor	parsley seed	0
00300566	CHRYSANTHEMYL ALCOHOL	C10H18O	154.25		reduction product of pyrrethrin constituent	0
00100584	GITOXIGENIN DIACETATE	C27H38O7	474.59		aglycon of GITOXIN	0
00270051	METHYL DEOXYCHOLATE	C25H42O4	406.61		rabbit bile & feces	0
01500707	CITROPTEN	C11H10O4	206.20	photosensitizing agent	Bergamot oil	0
00100129	8-HYDROXYCARAPINIC ACID	C26H30O8	470.52		*Meliaceae spp*	0
01500843	LATHOSTEROL	C27H46O	386.66		*Austeria rubens*	0
01503989	GLUCITOL-4-GUCOPYANOSIDE	C12H24O11	344.31		derivative cotton	0
01500704	N-ACETYLPROLINE	C7H11NO3	157.17	antirheumatic	synthetic	0
01505297	PERILLYL ALCOHOL	C10H16O	152.23	antineoplastic. apoptosis inducer; skin irritant. LD50(rat) 2100 mg/kg po	*Ocimum gratissimum*	0
01500485	PHENYTOIN SODIUM	C15H11N2NaO2	274.25	anticonvulsant. antieleptic	synthetic	0
02300009	TILORONE	C25H34N2O3	410.56	antiviral	synthetic	0
01501129	CARBETAPENTANE CITRATE	C26H39NO10	525.60	antitussive	synthetic	0
01500504	PROCAINE HYDROCHLORIDE	C13H21ClN2O2	272.77	anesthetic (local)	synthetic	0
01500519	PYRILAMINE MALEATE	C21H27N3O5	401.46	antihistaminic	synthetic	0
01503708	TERFENADINE	C32H41NO2	471.68	H1 antihistamine. nonsedating	synthetic	0
01505464	THIAMINE	C12H18Cl2N4O S	337.27	vitamin B1. enzyme cofactor	rice husks. wheat germ. yeast	0
01504214	TRIMETOZINE	C14H19NO5	281.31	sedative. neurosedative	synthetic	0
01503269	PERGOLIDE MESYLATE	C20H30N2O3S2	410.60	dopamine receptor agonist	semisynthetic	0
01505756	ITRACONAZOLE	C35H38Cl2N8O 4	705.65	antifungal	synthetic; R51211	0
01504116	FORMESTANE	C20H28O2	300.44	antineoplastic. aromatase inhibitor	synthetic; CGP-32349	0
01505755	IDEBENONE	C19H30O5	338.44	cognition enhancer. nootropic	synthetic	0
01505729	DOCOSANOL	C22H46O	326.61	antiviral	*Pygeum africanum*	0
01505688	PROPARACAINE HYDROCHLORIDE	C16H27ClN2O3	330.85	local anesthetic	synthetic	0
01505892	FUSARIC ACID	C9H11NO2	165.19	antiproliferative. dopamine beta-	*Fusarium spp*	0

				hydroxylase inhibitor		
01504515	DIACETAMATE	C10H11NO3	193.20	analgesic. antiinflammatory	synthetic	0
01505851	ARIPIPRAZOLE	C23H27Cl2N3O2	448.39	antipsychotic. 5HT2A antagonist	synthetic; OPC-14597. OPC-31	0
01505323	CARBIMAZOLE	C7H10N2O2S	186.23	antithyroid	synthetic	0
01504098	PHENOTHRIN	C23H26O3	350.46	ectoparasiticide	synthetic; S-2539-F	0
00300058	EPI(13)TORULOSOL	C20H34O2	306.49		*Cryptomeria japonica and Larix sibirica*	0
00201310	2'-METHOXYFORMONETIN	C17H14O5	298.29		*Eschscholtzia californica*	0
01500764	KINETIN	C10H9N5O	215.21	auxin. plant growth regulator. plant cell division promotor	yeast	0
01503391	D-PHENYLALANINE	C9H11NO2	165.19	antidepressant	synthetic	0
01500851	CHOLEST-4.6-DIEN-3-ONE	C27H42O	382.63		*Pinus spp*	0
01503928	5-METHYLFURMETHIDE	C9H16INO	281.13	muscarinic agonist	synthetic	0
01505250	MADECASSIC ACID	C30H48O6	504.71	wound healing	*Centella asiatica*	0
01500159	CARBAMAZEPINE	C15H12N2O	236.27	analgesic. anticonvulsant	synthetic	0
01500433	NITROFURANTOIN	C8H6N4O5	238.16	antibacterial	synthetic	0
01505385	ATOMOXETINE HYDROCHLORIDE	C16H20ClNO	277.79	norepinephrine reuptake inhibitor	synthetic; LY-139603	0
01501144	SULFADIMETHOXINE	C12H14N4O4S	310.33	antibacterial	synthetic	0
01502025	SUCCINYLSULFATHIAZOLE	C13H13N3O5S2	355.39	antibacterial	synthetic	0
01504263	ROSIGLITAZONE	C18H19N3O3S	357.43	antidiabetic	synthetic	0
01500141	BENZTHIAZIDE	C15H14ClN3O4S3	431.94	diuretic. antihypertensive	synthetic	0
01500182	CHLOROXYLENOL	C8H9ClO	156.61	antibacterial. topical and urinary antiseptic	synthetic	0
01500192	CLIDINIUM BROMIDE	C22H26BrNO3	432.36	anticholinergic	synthetic	0
01500302	FLUOCINOLONE ACETONIDE	C24H30F2O6	452.49	glucocorticoid. antiinflammatory	semisynthetic	0
01500247	DIGOXIN	C41H64O14	780.95	cardiac stimulant	*Digitalis lanata or D. orientalis Lam.. Scrophulariaceae*	0
01500201	CLOXACILLIN SODIUM	C19H17ClN3NaO5S	457.87	antibacterial	semisynthetic	0
01500213	CYCLOPHOSPHAMIDE HYDRATE	C7H17Cl2N2O3P	279.10	antineoplastic. alkylating agent	synthetic	0
01500346	HYOSCYAMINE	C17H23NO3	289.37	anticholinergic. analgesic	*Atropa. Datura and Hyoscyamus spp*	0
01505417	CAPOBENIC ACID	C16H23NO6	325.36	antiarrhythmic	synthetic; C-3	0

01505432	FOMEPIZOLE HYDROCHLORIDE	C4H7ClN2	118.56	alcohol dehydrogenase inhibitor	synthetic; 4-MP	0
02300154	ACECAINIDE HYDROCHLORIDE	C15H24ClN3O2	313.83	antiarrhythmic	synthetic	0
00310006	HYDRASTININE HYDROCHLORIDE	C11H14ClNO3	243.69	cardiotonic. uterine hemostatic	derivative of hydrastine (01501009)	0
01503939	RIBOSTAMYCIN SULFATE	C17H36N4O14S	552.55	antibacterial	*Streptomyces ribosidificus*	0
00200428	ROCCELLIC ACID	C17H32O4	300.44		*Lecanora and Roccella spp*	0
01601021	PAEONOL	C9H10O3	166.17	antibacterial	*Paeonia montan. Xanthorrhoea spp*	0
01700330	MUNDULONE ACETATE	C28H28O7	476.53		derivative	0
01601000	MEVASTATIN	C23H34O5	390.52	antihyperlipidemic. HMGCoA reductase inhibitor	*Penicillium brevicompactum; Compactin*	0
01503043	BUCLADESINE	C18H24N5O8P	469.39	vasodilator	synthetic	0
01503620	SAFROLE	C10H10O2	162.19	anesthetic (topical) and antiseptic. pediculicide	*sassafras officinale*	0
01500147	BISACODYL	C22H19NO4	361.40	cathartic	synthetic	0
01501016	FENBENDAZOLE	C15H13N3O2S	299.35	anthelmintic	synthetic	0
01500151	BROMOCRIPTINE MESYLATE	C33H44BrN5O8S	750.71	prolactin inhibitor. antiparkinsonian	semisynthetic	0
01505803	PRAVASTATIN SODIUM	C23H35NaO7	446.52	antihyperlipidemic. HMGCoA reductase inhibitor	CS-514; SQ-31000	0
01500286	ESTRONE	C18H22O2	270.37	estrogen	pregnacy urine. dates and *Punica granatum* seeds	0
01500670	BETAHISTINE HYDROCHLORIDE	C8H14Cl2N2	209.12	vasodilator	synthetic	0
01500348	IMIPRAMINE HYDROCHLORIDE	C19H25ClN2	316.87	antidepressant	synthetic	0
01504183	TRIFLURIDINE	C10H11F3N2O5	296.20	antiviral (opthalmic)	synthetic	0
01505498	ACEPROMAZINE MALEATE	C23H26N2O5S	442.53	sedative	synthetic	0
01503036	DINITOLMIDE	C8H7N3O5	225.16	antiprotozoal	synthetic	0
01504502	BITOSCANATE	C8H4N2S2	192.26	anthelmintic	synthetic; 16842	0
00300001	ORSELLINIC ACID	C8H8O4	168.15		common lichen constituent	0
00201727	EVERNINIC ACID	C8H8O5	184.15		oak moss lichen	0
00270049	alpha-HYDROXYDEOXYCHOLIC ACID	C24H40O4	392.58		pig bile	0
01500765	NARINGIN	C27H32O14	580.54	antihaemorrhagic.	*Citrus spp*	0

				antiinflammatory		
00300032	ISOBERGAPTENE	C12H8O4	216.19		*Heracleum and other Umbelliferaceae; mp 223-224 C*	0
01505002	LAPPACONITINE	C32H44N2O8	584.71	analgesic. antiarrhythmic	*Aconitum spp and Delphinium cashmirianum*	0
01504029	LIGUSTILIDE	C12H14O2	190.24	antispasmodic. smooth muscle relaxant	Ligusticum and Angelica spp	0
01500146	BETHANECHOL CHLORIDE	C7H17ClN2O2	196.67	cholinergic	synthetic	0
01501154	RONIDAZOLE	C6H8N4O4	200.15	antiprotozoal	synthetic	0
01500162	CARISOPRODOL	C12H24N2O4	260.33	muscle relaxant (skeletal)	synthetic	0
01505389	TRAMADOL HYDROCHLORIDE	C16H26ClNO2	299.84	analgesic	synthetic; U-26225A. CG-315E	0
01501202	GALANTHAMINE HYDROBROMIDE	C17H22BrNO3	368.27	anticholinesterase. analgesic. antiAlzheimer	*Galanthus. Narcissus and other Lillaceae*	0
01500451	OXYBENZONE	C14H12O3	228.24	ultraviolet screen	synthetic	0
01500428	NEOSTIGMINE BROMIDE	C12H19BrN2O2	303.20	cholinergic	synthetic	0
01506066	DEBRISOQUIN SULFATE	C10H15N3O4S	273.31	anti-hypertensive	synthetic; RO-5-3307/1	0
01500553	SULFATHIAZOLE	C9H9N3O2S2	255.31	antibacterial	synthetic	0
01500503	PROCAINAMIDE HYDROCHLORIDE	C13H22ClN3O	271.79	antiarrhythmic	synthetic	0
01500351	INDOPROFEN	C17H15NO3	281.31	analgesic. antiinflammatory	synthetic	0
01500210	CROMOLYN SODIUM	C23H14Na2O11	512.34	antiasthmatic. antiallergy	synthetic	0
01500689	LIDOCAINE HYDROCHLORIDE	C14H23ClN2O	270.80	anesthetic (local). antiarrhythmic	synthetic	0
01500324	HALAZONE	C7H5Cl2NO4S	270.09	antiinfectant	synthetic	0
01505708	EPIRUBICIN HYDROCHLORIDE	C27H30ClNO11	579.99	antineoplastic	synthetic; IMI-28	0
01505453	PYRIDOXINE	C8H11NO3	169.18	vitamin B6. enzyme cofactor	rice husks. wheat germ. yeast	0
01504912	OLSELTAMIVIR PHOSPHATE	C15H29N2O8P	396.38	antiviral	synthetic	0
02300173	BUSPIRONE HYDROCHLORIDE	C21H32ClN5O2	421.97	5HT1a receptor agonist. anxiolytic	synthetic	0
01506064	CHLOROPYRAMINE HYDROCHLORIDE	C16H21Cl2N3	326.27	antihistamine	synthetic	0
01505906	ZILEUTON	C11H12N2O2S	236.29	5-lipoxygenase inhibitor	synthetic; Abbott-64077	0
00201477	MUNDOSERONE	C19H18O6	342.35		*Mundulea suberosa; mp 156-161 C*	0
00300020	1-MONOPALMITIN	C19H38O4	330.51		*Asparagus. Momodica spp;* widespread in plants	0

01500754	XANTHURENIC ACID	C10H7NO4	205.17	caspase activator. guanylyl cyclase stimulant	pathological metabolite of tryptophan and kynurenine	0
00210211	EPIAFZELECHIN TRIMETHYL ETHER	C18H20O5	316.35		derivative	0
01504166	ISOOSAJIN	C25H24O5	404.46		*Maclura pomifera*	0
01502244	AMYGDALIN	C20H27NO11	457.43	antiinflammator y. experimental antineoplastic	*Rosaceae spp*	0
01504132	6.3'-DIMETHOXYFLAVONE	C17H14O4	282.29		*Pimelia decora*	0
00210567	DIMETHYLCAFFEIC ACID	C11H12O4	208.21		*Piper methysticum. Veronica virginica*	0
01505269	TANGERITIN	C20H20O7	372.37		*Citrus spp. Fortunella japonica*	0
01503987	CAFFEIC ACID	C9H8O4	180.16		widespread in plants	0
00300607	RUTOSIDE (rutin)	C27H30O16	610.53	vascular protectant	*Ruta graveolens. widespread in plants*	0
01500396	METHIMAZOLE	C4H6N2S	114.16	antihyperthyroi d	synthetic	0
00300034	TESTOSTERONE PROPIONATE	C22H32O3	344.49	androgen. antineoplastic	semisynthetic	0
01504526	FIROCOXIB	C17H20O5S	336.40	analgesic. antiinflammator y. antipyretic. COX-II inhibitor	synthetic; ML-1785713	0
02300332	PODOFILOX	C22H22O8	414.41	antineoplastic. inhibits microtubule assembly. and human DNA topoisomerase II; antimitotic agent	*Podophylum peltatum; podophylotoxin*	0
01500502	PROBENECID	C13H19NO4S	285.36	uricosuric	synthetic	0
01500258	DIPHENYLPYRALINE HYDROCHLORIDE	C19H24ClNO	317.86	antihistaminic	synthetic	0
01503276	ROXITHROMYCIN	C41H76N2O15	837.06	antibacterial	semisynthetic; RU-28965. RU-965	0
01505761	OXYTHIAMINE CHLORIDE HYDROCHLORIDE	C12H17Cl2N3O 2S	338.25	thiamine antagonist	synthetic	0
00300564	MENTHONE	C10H18O	154.25		pennyroyal and peppermint oils	0
00201342	DEOXYSAPPANONE B 7.3'-DIMETHYL ETHER ACETATE	C20H20O6	356.37		derivative *Caesalpinia sappan*	0
00231084	UMBELLIFERONE	C9H6O3	162.14	antifungal. phytoalexin	*Angelica. Artemisia. Coronilla. Ferula and Ruta spp*	0

01504176	RHETSININE	C19H17N3O2	319.36		*Zanthoxylum rhetsa. Evodia rutaecarpa*	0
01500657	HYDROQUINIDINE	C20H26N2O2	326.44	antiarrhythmic. antimalarial	*Cinchona bark*	0
01503906	ANISOMYCIN	C14H19NO4	265.31	antiprotozoal. antifungal. protein synthesis inhibitor	*Streptomyces griseolus*	0
01503815	CEVADINE	C32H49NO9	591.74	antihypertensive	*Veratrum alba*; contains 20% veratridine	0
01505001	PUERARIN	C21H20O9	416.38	beta-adrenergic blocker	*Pueraria spp*	0
01506088	TRIMETAZIDINE DIHYDROCHLORIDE	C14H24Cl2N2O 3	339.26	anti-anginal	synthetic; 400045	0
01500391	METHACHOLINE CHLORIDE	C8H18ClNO2	195.69	cholinergic. diagnostic aid	synthetic	0
01505376	ACEDAPSONE	C16H16N2O4S	332.38	antimalarial. leprostatic	synthetic; CI-556. CN-1883. DADDS. PAM-MAR-1165	0
01500464	PENICILLAMINE	C5H11NO2S	149.21	chelating agent (Cu). antirheumatic	semisynthetic	0
01500110	AMANTADINE HYDROCHLORIDE	C10H18ClN	187.71	antiviral. antiparkinsonian; treatment of drug-induced extrapyrimidal reactions	synthetic	0
01500406	METHYLPREDNISOLONE	C22H30O5	374.48	glucocorticoid	semisynthetic	0
01500208	COTININE	C10H12N2O	176.21	antidepressant	*Nicotiana tabacum*	0
01500352	INOSITOL	C6H12O6	180.15	growth factor	lipotropic polyol widely distributed in plants and animals	0
01500532	SALICYLAMIDE	C7H7NO2	137.13	analgesic	synthetic	0
01500310	FUROSEMIDE	C12H11ClN2O5 S	330.74	diuretic. antihypertensive	synthetic	0
01500217	CYTARABINE	C9H13N3O5	243.22	antineoplastic. antiviral. antimetabolite	synthetic	0
01500767	BUTAMBEN	C11H15NO2	193.24	anesthetic (local)	synthetic	0
01505482	NETILMICIN SULFATE	C21H45N5O15S 2	671.74	antibacterial	semisynthetic; SCH-20569	0
01502029	CEFSULODIN SODIUM	C22H19N4NaO8 S2	554.53	antibacterial	semisynthetic	0
01505865	6-HYDROXYTROPINONE	C8H13NO2	155.19		synthetic	0
01503108	BROMOPRIDE	C14H22BrN3O2	344.25	antiemetic	synthetic	0
01506024	NAFTOPIDIL DIHYDROCHLORIDE	C24H30Cl2N2O 3	465.42	antihypertensive	synthetic	0
01505315	6-AMINONICOTINAMIDE	C6H7N3O	137.14	antineoplastic. apoptosis inducer	synthetic	0
01400136	N-METHYLISOLEUCINE	C7H15NO2	145.20		*Phasealus vulgaris*	0

01505959	DIPTERYXIN	C17H14O6	314.29		*Dipteryx odorata*	0
00100618	3alpha-HYDROXY-4.4-BISNOR-8.11.13-PODOCARPATRIENE	C15H20O	216.32		semisynthetic	0
01505302	GENETICIN	C20H44N4O18S2	692.71	antibacterial	*Micromonospora spp; G-418*	0
00100743	HOMOPTEROCARPIN	C17H16O4	284.31		*mp 82-84 C; Pterocarpus santalinus*	0
01504022	SALSOLIDINE	C12H17NO2	207.27	antihypertensive	*Salsola Richteri*	0
01504135	TRIMEDLURE	C12H21ClO2	232.75	arthropod pheromone	synthetic; 5-Cl isomer present	0
01505930	SR-2640	C23H18N2O3	370.41	LTD4/ LTE4 antagonist	synthetic; 2[3-(quinolin-2-ylmethoxy)phenylamino]benzoic acid	0
01500160	CARBENICILLIN DISODIUM	C17H16N2Na2O6S	422.37	antibacterial	semisynthetic	0
01500615	ZOMEPIRAC SODIUM	C15H14ClNO3	291.73	analgesic. antiinflammatory	synthetic	0
01506084	PROSCILLARIDIN	C29H40O9	532.63	cardiotonic	*Scilla spp; A-32686. 2936*	0
01500102	ACETAZOLAMIDE	C4H6N4O3S2	222.24	carbonic anhydrase inhibitor. diuretic. antiglaucoma	synthetic	0
01500244	DIETHYLSTILBESTROL	C18H20O2	268.35	estrogen	synthetic	0
01500587	TRIAMCINOLONE ACETONIDE	C24H31FO6	434.50	antiinflammatory	semisynthetic	0
01500246	DIGITOXIN	C41H64O13	764.95	inotropic. cardiotonic	*Digitalis spp*	0
01500236	DIBUCAINE HYDROCHLORIDE	C20H30ClN3O2	379.93	anesthetic (local)	synthetic	0
01500198	CLONIDINE HYDROCHLORIDE	C9H10Cl3N3	266.55	antihypertensive	synthetic	0
01500601	TUAMINOHEPTANE SULFATE	C7H19NO4S	213.29	adrenergic agonist	synthetic	0
01505203	EZETIMIBE	C24H21F2NO3	409.43	sterol absorption inhibitor	synthetic	0
01501130	FAMPRIDINE	C5H6N2	94.116	K channel blocker; multiple sclerosis therapy	synthetic	0
01502254	MENADIONE	C11H8O2	172.18	prothrombogenic agent	*Asplenium and Juglans spp.*	0
01501152	SPIPERONE	C23H26FN3O2	395.48	antipsychotic	synthetic; R-5147	0
01503205	CITIOLONE	C6H9NO2S	159.20	hepatoprotectant	synthetic	0
01500647	VINCAMINE	C21H26N2O3	354.45	vasodilator	*Vinca minor*	0
01505372	SUMATRIPTAN	C14H21N3O2S	295.40	5HT agonist	synthetic; GR-43175	0
00100223	1.2alpha-EPOXYDEACETOXYDIHYDROGEDUNIN	C26H32O7	456.54		*Meliaceae spp*	0
01401419	DERRUSTONE	C18H14O6	326.30		*Derris robusta*	0

00210369	GALLIC ACID	C7H6O5	170.12	antineoplastic. astringent. antibacterial	insect galls	0
01500996	GLAFENINE	C19H17ClN2O4	372.81	analgesic	synthetic; R-11707	0
01505334	SECURININE	C13H15NO2	217.27	GABAA receptor blocker. CNS stimulant	*Securinega spp and Phyllanthus discoides*	0
01505135	PIPLARTINE	C17H19NO5	317.34	anti-asthma. antibronchitis	*Piper spp*	0
00240645	RETUSIN 7-METHYL ETHER	C17H14O5	298.29		derivative *Dalbergia spp*	0
01502003	BUFEXAMAC	C12H17NO3	223.27	antiinflammatory. analgesic. antipyretic	synthetic; CP-1044-J3	0
01506044	THIOGUANOSINE	C10H13N5O4S	299.31	antineoplastic	synthetic	0
01500172	CHLORAMPHENICOL PALMITATE	C27H42Cl2N2O6	561.55	antibacterial. antirickettsial	semisynthetic	0
01500448	OXACILLIN SODIUM	C19H18N3NaO5S	423.42	antibacterial	semisynthetic	0
01500156	CAMPHOR (1R)	C10H16O	152.23	analgesic. antiinfective. antipruritic	*Cinnamomum camphora*	0
01501215	KETOPROFEN	C16H14O3	254.28	antiinflammatory	synthetic	0
01500524	QUININE SULFATE	C20H26N2O6S	422.50	antimalarial. skeletal muscle relaxant	*Cinchona spp*	0
01500347	IBUPROFEN	C13H18O2	206.28	antiinflammatory	synthetic	0
01500349	INDAPAMIDE	C16H16ClN3O3S	365.84	diuretic. antihypertensive	synthetic	0
01501126	5-AMINOPENTANOIC ACID HYDROCHLORIDE	C5H12ClNO2	153.60	GABAb antagonist	synthetic	0
01503320	TIMONACIC	C4H7NO2S	133.16	hepatoprotectant	synthetic	0
01503381	PASINIAZID	C13H14N4O4	290.28	antibacterial (tuberculostatic)	synthetic; RD-325	0
01503628	PRONETALOL HYDROCHLORIDE	C15H20ClNO	265.78	beta adrenergic agonist. antiarrhythmic	synthetic; ICI-38174. AY-6204	0
01505245	TROXERUTIN	C33H42O19	742.69	vasoprotectant	derivative; rutin 7.3'.4'-trihydroxyethyl ether; Z-6000. THR	0
01503822	RAMIFENAZONE	C14H20ClN3O	281.78	analgesic. antipyretic. antiinflammatory	synthetic	0
01504100	METHOPRENE (S)	C19H34O3	310.48	ectoparasiticide	synthetic	0
01500872	PALMATINE CHLORIDE	C21H22ClNO4	387.86	antibacterial. antimalarial. uterine contractant	*Jateorhiza palmata (Calumba root)*	0
01505242	LUPANINE PERCHLORATE	C15H25ClN2O5	348.82		*Lupinus and Cystisus spp*	0
01503947	CACODYLIC ACID	C2H7AsO2	137.99	antieczema. dermatologic. herbicide	synthetic	0
01500705	O-BENZYL-l-SERINE	C10H13NO3	195.22		synthetic	0

01505337	ELAIDYLPHOSPHOCHOLIN E	C23H48NO4P	433.61	antineoplastic	synthetic	0
01505317	CARMOFUR	C11H16FN3O3	257.26	antineoplastic	synthetic	0
02300061	CLOMIPRAMINE HYDROCHLORIDE	C19H24Cl2N2	351.32	antidepressant	synthetic	0
01506082	PENTETIC ACID	C14H23N3O10	393.35	chelating agent. diagnostic aid	synthetic; DTPA	0
00300548	SPARTEINE SULFATE	C15H28N2O4S	332.46	oxytocic	*Lupinus spp and other Leguminosae*	0
01500111	AMIKACIN SULFATE	C22H47N5O21S 2	781.76	antibacterial	semisynthetic	0
01500174	CHLORAMPHENICOL	C11H12Cl2N2O 5	323.13	antibacterial. antirickettsial. inhibits protein synthesis	*Streptomyces venezuelae*	0
01502040	CEFMETAZOLE SODIUM	C15H16N7NaO5 S3	493.52	antibacterial	semisynthetic	0
01501121	MEMANTINE HYDROCHLORIDE	C12H22ClN	215.76	muscle relaxant (skeletal)	synthetic	0
00330071	LINDANE	C6H6Cl6	290.83	insecticide	synthetic; gamma-BHC	0
01500157	CAPREOMYCIN SULFATE	C25H46N14O12 S	766.79	antibacterial. tuberculostatic	*Streptomyces capreolis*	0
01500527	RESORCINOL	C6H6O2	110.11	keratolytic. antiseborheic	coal tar	0
01500651	STRYCHNINE	C21H22N2O2	334.42	central stimulant	*Strychnos nux-vomica and other Strychnos spp*	0
01500531	SALICYL ALCOHOL	C7H8O2	124.14	anesthetic (local). antiinflammator y	*Populus sieboldii*	0
01500200	CLOTRIMAZOLE	C22H17ClN2	344.84	antifungal	synthetic	0
01500604	UREA	CH4N2O	60.05	diuretic	synthetic	0
01500687	HYDRASTINE (1R. 9S)	C21H21NO6	383.40	antihypertensive . sedative. antibacterial	*Corydalis spp*	0
01900002	THIAMYLAL SODIUM	C12H17N2NaO2 S	276.33	anesthetic	synthetic	0
01506056	ZARDAVERINE	C12H10F2N2O3	268.22	PDE III & IV inhibitor. antiallergic	synthetic	0
01505819	ELETRIPTAN HYDROBROMIDE	C22H27BrN2O2 S	463.44	5-HT agonist. anti-migrane	synthetic; UK-116044-04	0
01503230	NIFENAZONE	C17H16N4O2	308.34	analgesic. antiinflammator y	synthetic	0
01503218	HEPTAMINOL HYDROCHLORIDE	C8H20ClNO	181.70	vasodilator	synthetic	0
01500821	BICUCULLINE (+)	C20H17NO6	367.36	GABAa antagonist	*Dicentra cucullaria. Corydalis spp*	0
01504115	HIERACIN	C15H10O7	302.24		*Ginkgo biloba. Hieracium pilosella* and *Isoetes spp*	0
01505129	PLUMBAGIN	C11H8O3	188.18	antibacterial. antifungal. tuberculostatic; antifeedant	Aristea. Diospyros & Plumbago spp;	0

				(African army worms)	Dyerophyton. Drosera. Dioncophyllum. Nepenthe. Sisyrinchium and Sparaxis tricolor	
01800009	GUAIOL(-)	C15H26O	222.37		*Callitris intratropica. Eucalyptus maculata. Drimys lancelolata*	0
01502129	ISOGUVACINE HYDROCHLORIDE	C6H10ClNO2	163.60	GABA agonist	synthetic	0
01503403	EXALAMIDE	C13H19NO2	221.30	antifungal	synthetic	0
01503425	ZOPICLONE	C17H17ClN6O3	388.81	hypnotic. sedative	synthetic	0
01500173	CHLORAMPHENICOL HEMISUCCINATE	C15H16Cl2N2O8	423.20	antibacterial. antirickettsial. inhibits protein synthesis	semisynthetic	0
01501120	MINAPRINE HYDROCHLORIDE	C17H24Cl2N4O	371.31	psychotropic	synthetic	0
01500422	NALOXONE HYDROCHLORIDE	C19H22ClNO4	363.84	narcotic antagonist	synthetic	0
01505384	METHYLDOPATE HYDROCHLORIDE	C12H18ClNO4	275.73	antihypertensive	synthetic	0
01500334	HYDRALAZINE HYDROCHLORIDE	C8H9ClN4	196.64	antihypertensive	semisynthetic	0
01500539	SPIRONOLACTONE	C24H32O4S	416.58	diuretic	synthetic	0
01500516	PSEUDOEPHEDRINE HYDROCHLORIDE	C10H16ClNO	201.69	decongestant	synthetic	0
01505440	LOBENDAZOLE	C10H11N3O2	205.21	anthelmintic	synthetic; SK&F-24529; NSC-42044	0
01506085	OXEDRINE	C9H13NO2	167.20	anti-obesity	citrus aurantium; SYNEPHRINE	0
01504518	EFLOXATE	C19H16O5	324.33	coronary vasodilator	synthetic	0
01505957	ARSENIC TRIOXIDE	As4O6	395.68	antineoplastic. antileukemia	mineral	0
00201538	DECAHYDROGAMBOGIC ACID	C38H54O8	638.84		derivative	0
00100102	CARAPIN-8(9)-ENE	C27H30O7	466.53		*Carapa and Cedrela species*	0
01504016	HEDERAGENIN	C30H48O4	472.71		*Clematis. Hedera spp*	0
01501138	PIRENZEPINE HYDROCHLORIDE	C19H23Cl2N5O2	424.33	antiulcer	synthetic	0
01501115	NALBUPHINE HYDROCHLORIDE	C21H28ClNO4	393.91	analgesic. narcotic antagonist	synthetic	0
01500680	ARECOLINE HYDROBROMIDE	C8H14BrNO2	236.11	anthelmintic (Cestodes). hypotensive. cathartic	betel nuts *(Arica catechu)*	0
01500483	PHENYLEPHRINE HYDROCHLORIDE	C9H14ClNO2	203.67	mydriatic. decongestant	synthetic	0
01500240	DICYCLOMINE HYDROCHLORIDE	C19H36ClNO2	345.95	anticholinergic	synthetic	0

01500515	PROPYLTHIOURACIL	C7H10N2OS	170.23	antihyperthyroid	synthetic	0
01501133	NICERGOLINE	C24H26BrN3O3	484.39	vasodilator	synthetic	0
00201331	DEOXYSAPPANONE B TRIMETHYL ETHER	C19H20O5	328.36		*Caesalpinia sappan*	0
01500867	HARMINE	C13H12N2O	212.25	antiparkinsonian. CNS stimulant	*Peganium harmala*	0
00310015	CEDRYL ACETATE	C17H28O2	264.41		semisynthetic	0
00100058	6-HYDROXYANGOLENSIC ACID METHYL ESTER	C27H34O8	486.56		*Meliaceae spp*	0
01505268	NOBILETIN	C21H22O8	402.40	matrix metaloproteinase inhibitor; antineoplastic	*Citrus spp*	0
01505380	3.4'.5.6.7-PENTAMETHOXYFLAVONE	C20H20O7	372.37		*Citrus spp*	0
01504068	DIOSMETIN	C16H12O6	300.27		*Valeriana. Digitalis spp*	0
01505080	SALVINORIN A	C23H28O8	432.47	k-opiod receptor agonist. psychotropic	*Salvia divinorum*	0
01500399	METHOXAMINE HYDROCHLORIDE	C11H18ClNO3	247.72	alpha1 adrenoreceptor agonist. vasoconstrictor	synthetic	0
01500568	THEOPHYLLINE	C7H8N4O2	180.16	bronchodilator	*Camelia. thea. Paullinia cupana*	0
01501127	ATENOLOL	C14H22N2O3	266.34	beta adrenergic blocker	synthetic	0
01505020	HOMOSALATE	C16H22O3	262.35	UV screen. analgesic	synthetic	0
01500179	CHLOROQUINE DIPHOSPHATE	C18H32ClN3O8P2	515.87	antimalarial. antiamebic. antirheumatic. intercalating agent	synthetic	0
01500591	TRIFLUOPERAZINE HYDROCHLORIDE	C21H26Cl2F3N3S	480.42	antipsychotic	synthetic	0
01500538	SPECTINOMYCIN HYDROCHLORIDE	C14H26Cl2N2O7	405.27	antibacterial	*Streptomyces spectabilis*	0
01505414	BROMINDIONE	C15H9BrO2	301.14	anticoagulant	synthetic	0
01505728	PHTHALYLSULFACETAMIDE	C16H14N2O6S	362.36	antibacterial	synthetic	0
01505979	ETHYNODIOL DIACETATE	C24H32O4	384.52	progestin	semisynthetic; SC-11800	0
00100749	STROPHANTHIDINIC ACID LACTONE ACETATE	C25H32O7	444.52		semisynthetic	0
00200848	DEOXYSAPPANONE B 7.3'-DIMETHYL ETHER	C18H18O5	314.34		*Caesalpinia sappan*	0
01502261	QUEBRACHITOL	C7H14O6	194.18		common in dicotyledons	0
00300532	ANDROGRAPHOLIDE	C20H30O5	350.45		*Adrographis peniculata*	0
01500703	ACETYLGLUTAMIC ACID	C7H11NO5	189.16	excitatory aminoacid	synthetic	0
01500398	METHOTREXATE(+/-)	C20H22N8O5	454.44	antineoplastic. antirheumatic. folic acid antagonist	synthetic; AMETHOPTERIN	0

01500495	PRAZOSIN HYDROCHLORIDE	C19H22ClN5O4	419.87	antihypertensive	synthetic	0
01502018	BRETYLIUM TOSYLATE	C18H24BrNO3S	414.36	inhibitor of norepinephrine release	synthetic	0
01503260	NADOLOL	C17H27NO4	309.40	betaadrenergic blocker	synthetic	0
00300062	METAMECONINE	C10H10O4	194.18		*Accacia crombei*	0
01505781	SELEGILINE HYDROCHLORIDE	C13H18ClN	223.74	antidepressant. MAO inhibitor. antiparkinsonia n	synthetic	0
01500898	EMODIN	C15H10O5	270.24	antibacterial. antineoplastic. cathartic. tyrosine kinase inhibitor	Cascara. Rheum and Rhammnus species	0
00310008	DJENKOLIC ACID	C7H14N2O4S2	254.32		djenkol bean (*Pithecolobium lobatum*)	0
00102007	FORMONONETIN	C16H12O4	268.27	phytoestrogen	soyabean and clover species	0
01505176	AURAPTENE	C19H22O3	298.38	antineoplastic. apoptosis inducer	*Citrus aurantium. Feronia elephantum. Aegle marmelos. Libanotis intermedia*	0
00100576	TRIDESACETOXYKHIVORIN	C26H36O7	460.57		*Khaya spp*	0
01505381	SINENSETIN	C20H20O7	372.37		*Citrus spp*	0
01500136	BECLOMETHASONE DIPROPIONATE	C28H37ClO7	521.05	antiasthmatic. topical antiinflammator y	synthetic	0
01501161	SUPROFEN	C14H12O3S	260.31	antiinflammator y	synthetic	0
01505602	FENRETINIDE	C26H33NO2	391.55	antineoplastic	synthetic; MCN-R-1967	0
01500425	NAPROXEN(+)	C14H14O3	230.26	antiinflammator y. analgesic. antipyretic	synthetic	0
01500183	CHLORPHENIRAMINE (S) MALEATE	C20H23ClN2O4	390.87	antihistaminic	synthetic	0
01502014	MESNA	C2H5NaO3S2	164.17	mucolytic	synthetic	0
01500938	PROTOVERATRINE B	C41H63NO15	809.95	antihypertensive . emetic; LD50(mouse) 0.21 mg/kg sc	*Veratrum album. V viride*	0
00300105	beta-CARYOPHYLLENE ALCOHOL	C15H26O	222.37		palmarosa oil. *Cymbopogon martini*	0
01505117	ESTRAGOLE	C10H12O	148.20	insect atttractant. skin irritant. carcinogen	numerous plant essential oils	0
01500984	GINKGOLIDE A	C20H24O11	440.40	antibacterial	*Ginkgo biloba*	0
01504015	alpha-MANGOSTIN	C24H26O6	410.47		*Garcinia mangostana. Hydnocarpus*	0

					octandra. H venenata	
01506051	TRIMETHYLCOLCHICINIC ACID	C19H21NO5	343.38	antineoplastic	derivative	0
01500728	5.7-DIHYDROXY-4-METHYLCOUMARIN	C10H8O4	192.17		semisynthetic	0
01500534	SCOPOLAMINE HYDROBROMIDE	C17H22BrNO4	384.27	anticholinergic. treatment of motion sickness	*Scopolia. Datura. Atropa spp*	0
01506009	METHIMAZOLE	C4H6N2S	114.16	thyroid inhibitor	synthetic	0
01505271	CROTAMITON	C13H17NO	203.28	antipruritic. scabicide	synthetic	0
01504268	LOSARTAN	C22H23ClN6O	422.92	antihypertensive. AT1 angiotensin II antagonist	synthetic	0
01500117	AMITRIPTYLINE HYDROCHLORIDE	C20H24ClN	313.87	antidepressant	synthetic	0
01500202	CLOXYQUIN	C9H6ClNO	179.60	antibacterial. antifungal	synthetic	0
01500499	PREDNISONE	C21H26O5	358.43	glucocorticoid	semisynthetic	0
01500489	PIPERACILLIN SODIUM	C23H26N5NaO7S	539.54	antibacterial	semisynthetic	0
01500675	OLEANDOMYCIN PHOSPHATE	C35H64NO16P	785.87	antibacterial	*Streptomyces antibioticus*	0
01505436	D-LACTITOL MONOHYDRATE	C12H26O12	362.33	sweetener. treatment of portoencephalopathy	semisynthetic	0
01505562	NORGESTIMATE	C23H31NO3	369.50	progestin	semisynthetic	0
01503032	DIPYROCETYL	C11H10O6	238.19	antirheumatic. analgesic	synthetic	0
00210220	CATECHIN TETRAMETHYLETHER	C19H22O6	346.38		derivative	0
01504800	CHRYSANTHEMIC ACID	C10H16O2	168.23	esters as insecticide	esters as constituent of pyrethrum flowers	0
00100205	3alpha-ACETOXYDIHYDRODEOXYGEDUNIN	C30H40O7	512.64		*Meliaceae spp*	0
01504020	EVOXINE	C18H21NO6	347.37		*Evodia xanthoxyloides*	0
01504153	NORCANTHARIDIN	C8H8O4	168.15	antineoplastic. protein phosphatase inhibitor	synthetic	0
00300024	HYDROCORTISONE	C21H30O5	362.47	glucocorticoid. antiinflammatory	adrenal glands	0
01501147	SULFAMONOMETHOXINE	C11H12N4O3S	280.30	antibacterial	synthetic	0
01501134	PIMOZIDE	C28H29F2N3O	461.55	antipsychotic	synthetic	0
01500206	COLISTIMETHATE SODIUM	C57H103N16Na5O28S5	1735.81	antibacterial	*Bacillus colistinus*	0
01505116	ESTROPIPATE	C22H32N2O5S	436.57	estrogen	semisynthetic	0
01503009	BIOTIN	C10H16N2O3S	244.31	vitamin B complex	Vitamin B complex	0
01503920	CLOPERASTINE HYDROCHLORIDE	C20H25Cl2NO	366.33	antitussive	synthetic; HT-11	0
00200873	IRIGENIN TRIMETHYL ETHER	C21H22O8	402.40		derivative	0

01701001	ANDROSTA-1.4-DIEN-3.17-DIONE	C19H24O2	284.40		mammary neoplasms; cholesterol metabolite	0
01504167	URSINOIC ACID	C15H16O5	276.29		*Angelica ursina*	0
01500861	CORALYNE CHLORIDE	C22H22ClNO4	399.87	cytostatic. intercalating agent	semisynthetic	0
01501208	KARANJIN	C17H10O4	278.26		*Derris and Tephrosia spp.*	0
01501012	3-HYDROXYFLAVONE	C15H10O3	238.24		cabbage	0
01503637	METITEPINE MALEATE	C24H28N2O4S2	472.62	5HT1&2 receptor antagonist	synthetic	0
01502108	ANTIMYCIN A (A1 shown)	C27H38N2O9	534.61	antifungal. antiviral. interferes in cytochrome oxidation	*Streptomyces spp*	0
01504519	7-HYDROXYFLAVONE	C15H10O3	238.24	antifungal. analgesic	*Virola spp*	0
01503672	SODIUM THIOGLYCOLATE	C2H3NaO2S	114.09	dipilatory. irritant	synthetic	0
01505253	SINOMENINE	C19H23NO4	329.39	weak abortifacient. immunosuppres sant. analgesic. antiinflammator y; LD50 (po) 580 mg/kg; (ip) 285 mg/kg(mouse)	*Sinomenium acutum* and *Stephania cepharantha*	0
00307023	TESTOSTERONE	C19H28O2	288.43	androgen. antineoplastic	mamallian male hormone	0
01500177	CHLORHEXIDINE	C22H32Cl4N10	578.37	antibacterial (topical). disinfectant	synthetic	0
01500309	FURAZOLIDONE	C8H7N3O5	225.16	antibacterial	NF-180; synthetic	0
01500501	PRIMIDONE	C12H14N2O2	218.25	anticonvulsant	synthetic	0
01503600	NIMODIPINE	C21H26N2O7	418.45	vasodilator	synthetic	0
01503045	ACEXAMIC ACID	C8H15NO3	173.21	wound healing agent	synthetic	0
01505863	3-ACETAMIDOCOUMARIN	C11H9NO3	203.19		synthetic	0
00201606	RUTILANTINONE	C22H20O9	428.39	coccidiostat	*Streptomyces* species	0
01504018	HEDERACOSIDE C	C59H96O26	1221.40		*Hedera helix*	0
01504410	PICROPODOPHYLLIN	C22H22O8	414.41	Insulin growth factor 1 receptor inhibitor. antineoplastic	*Podophylum peltatum*; epimer of podophyllotox in; 10% cytotoxicity of podophyllotox in	0
01502207	L-LEUCYL-L-ALANINE	C9H18N2O3	202.25	ubiquitin blocker. neurite growth inhibitor	synthetic	0
00100465	DIHYDROFISSINOLIDE	C29H38O8	514.62		derivative of fissinolide (00100031)	0

01502092	5-FLUOROINDOLE-2-CARBOXYLIC ACID	C9H6FNO2	179.15	NMDA receptor antagonist (gly)	synthetic	0
01503641	1-PHENYLBIGUANIDE HYDROCHLORIDE	C8H12ClN5	213.67	5HT3 receptor agonist	synthetic	0
01504228	CRUSTECDYSONE	C27H44O7	480.64	insect molting hormone	silkworm moth *Bombyx mori* & the plant *Achyranthes fauriei*	0
01500902	SODIUM DEOXYCHOLATE	C24H39NaO4	414.56	choleretic	bile constituent	0
01500419	NADIDE	C21H27N7O14P2	663.43	alcohol and narcotic antagonist	synthetic	0
01501214	ENALAPRIL MALEATE	C24H32N2O9	492.53	ACE inhibitor. antihypertensive	synthetic	0
01500588	TRIAMCINOLONE DIACETATE	C25H31FO8	478.51	antiinflammatory	semisynthetic	0
01500596	TRIOXSALEN	C14H12O3	228.24	melanizing agent. antipsoriatic	synthetic	0
01501124	ACECLIDINE	C9H15NO2	169.22	cholinergic	synthetic	0
01504235	ROFECOXIB	C17H14O4S	314.36	COX2 inhibitor. antiinflammatory. antiarthritic	synthetic	0
01502202	CYCLOSPORINE	C62H111N11O12	1202.64	immunosuppressant	*Tolypocladium inflatum*	0
01503242	ISOXICAM	C14H13N3O5S	335.34	antiinflammatory	synthetic	0
01505805	GABAPENTIN	C9H17NO2	171.24	anticonvulsant	synthetic; CI-945; GOE-3450	0
01505777	DIATRIZOIC ACID	C11H9I3N2O4	613.91	radiopaque agent	synthetic	0
00240740	4-ACETOXYPHENOL	C8H8O3	152.15	antioxidant	*Ferulago aucheri. Salvia yosgadensis*	0
01701060	HYDROXYPROGESTERONE	C21H30O3	330.47	progestagen	urine and blood	0
01500659	ARTEMISIN	C16H20O4	276.33	anthelmintic	*Artemesia spp*	0
01506041	SALSOLINOL HYDROBROMIDE	C10H14BrNO2	260.13	prolactin-releasing factor	dopamine derivative	0
01504182	3-METHYLXANTHINE	C6H6N4O2	166.14	phosphodiesterase inhibitor	synthetic	0
01500376	MECLIZINE HYDROCHLORIDE	C25H29Cl3N2	463.88	antiemetic	synthetic	0
01400208	COUMARIN	C9H6O2	146.14	antineoplastic. antiinflammatory. antihyperglycaemic	*Coumarouna odorata. tonka beans. lavender oil*	0
01500103	ACETOHYDROXAMIC ACID	C2H5NO2	75.067	urease inhibitor. antiurolithic. antbacterial	synthetic	0
01500411	METOPROLOL TARTRATE	C19H31NO9	417.46	antihypertensive. antianginal	synthetic	0
01505407	ATROPINE OXIDE	C17H25NO4	307.39	anticholinergic	semisynthetic	0
01500467	PENICILLIN V POTASSIUM	C16H17KN2O5S	388.49	antibacterial	semisynthetic	0
01500188	CHLORZOXAZONE	C7H4ClNO2	169.56	muscle relaxant (skeletal)	synthetic	0

01500296	EUGENOL	C10H12O2	164.20	analgesic (topical). antiseptic. antifungal	common in plant essential oils	0
01500338	HYDROCORTISONE ACETATE	C23H32O6	404.50	glucocorticoid. antiinflammatory	semisynthetic	0
01505765	TRICHLORFON	C4H8Cl3O4P	257.43	anthelmintic	synthetic; Bayer-L-1359. DETF	0
01500899	ESCULETIN	C9H6O4	178.14	antifungal	*Aesculus and Solanaceae spp*	0
01502223	RESVERATROL	C14H12O3	228.24	antifungal. antibacterial	*Veratrum grandiflorum. Pinus sibirica. Vitis vinifera. Arachis hypogaea*	0
00200002	ORSELLINIC ACID. ETHYL ESTER	C10H12O4	196.20		*Roccella and Lecanora lichens*	0
01505339	L-PHENYLALANINOL	C9H13NO	151.21	antiulcer	synthetic	0
01505456	PARAMETHADIONE	C7H11NO3	157.17	anticonvulsant	synthetic	0
01500187	CHLORTHALIDONE	C14H11ClN2O4S	338.77	diuretic. antihypertensive	synthetic	0
01500311	FUSIDIC ACID	C31H48O6	516.72	antibacterial	*Fusidium spp*	0
01504300	ORLISTAT	C29H53NO5	495.74	reversible lipase inhibitor. antiobesity	synthetic	0
01504081	BETULINIC ACID	C29H46O3	442.68	antineoplastic	*Betula spp.*	0
00201138	DEGUELIN(-)	C23H22O6	394.42	antineoplastic. antiviral. insecticide	*Tephrosia & Derris spp*	0
01500862	BOLDINE	C19H21NO4	327.38		*Peumus boldus*	0
01505383	HEXAMETHYLQUERCETAGETIN	C21H22O8	402.40		*Citrus spp*	0
01505039	2.5-DI-t-BUTYL-4-HYDROXYANISOLE	C15H24O2	236.35	antioxidant	synthetic	0
01505785	CAMYLOFINE DIHYDROCHLORIDE	C19H34Cl2N2O2	393.40	anticholinergic	synthetic	0
01505849	ETHIONINE	C6H13NO2S	163.24	antineoplastic; inhibitor DNA methylation	synthetic	0
01500374	MECAMYLAMINE HYDROCHLORIDE	C11H22ClN	203.75	antihypertensive	synthetic	0
00310039	alpha-TOCHOPHEROL	C29H50O2	430.72	vitamin E	soya. wheat germ and other plant oils	0
00330001	DACTINOMYCIN	C62H86N12O16	1255.44	antineoplastic. intercalating agent	*Actinomyces spp*	0
01500175	CHLORCYCLIZINE HYDROCHLORIDE	C18H22Cl2N2	337.29	H1-antihistamine	synthetic	0
01500403	METHYLDOPA	C10H13NO4	211.21	antihypertensive	synthetic	0
01500488	PINDOLOL	C14H20N2O2	248.32	antihypertensive. antianginal. antiarrhythmic. antiglaucoma agent	synthetic	0
01503424	ALFAXALONE	C21H32O3	332.48	anesthetic	semisynthetic	0

01501159	SULMAZOLE	C14H13N3O2S	287.34	cardiotonic	synthetic	0
01504507	CHLORINDIONE	C15H9ClO2	256.69	antcoagulant. Vitamine K antagonist	synthetic; G25766	0
01500904	CHOLIC ACID. METHYL ESTER	C25H42O5	422.61		acid as primary bile constituent	0
01800005	MENTHYL BENZOATE	C17H24O2	260.37		derivative	0
01501142	SULFACHLORPYRIDAZINE	C10H9ClN4O2S	284.72	antibacterial	synthetic	0
01500101	ACETAMINOPHEN	C8H9NO2	151.16	analgesic. antipyretic	synthetic	0
01500115	AMINOGLUTETHIMIDE	C13H16N2O2	232.28	aromatase inhibitor. antineoplastic. testosterone suppressant	synthetic	0
01500207	CORTISONE ACETATE	C23H30O6	402.49	glucocorticoid	semisynthetic	0
01500521	PYRVINIUM PAMOATE	C49H43N3O6	769.90	anthelmintic	synthetic	0
01500487	PILOCARPINE NITRATE	C11H17N3O5	271.27	antiglaucoma agent. miotic	*Pilocarpus spp*	0
01500189	CICLOPIROX OLAMINE	C14H24N2O3	268.35	antifungal	synthetic	0
01500643	PHENETHICILLIN POTASSIUM	C17H19KN2O5S	402.51	antibacterial	semisynthetic	0
01502106	CARBOPLATIN	C6H12N2O4Pt	371.26	antineoplastic. convulsant	synthetic	0
01505780	DIPERODON HYDROCHLORIDE	C22H28ClN3O4	433.93	analgesic. anesthetic	synthetic	0
00300015	CHOLESTANE	C27H48	372.68		semisynthetic	0
00240958	4'-METHOXYFLAVONE	C16H12O3	252.27		*Sapindus saponaria.*	0
01505011	TRIMEBUTINE MALEATE	C26H33NO9	503.55	antispasmodic. opiod receptor agonist	synthetic	0
01501155	SULFAMETER	C11H12N4O3S	280.30	antibacterial	synthetic	0
01503932	OXYPHENCYCLIMINE HYDROCHLORIDE	C20H29ClN2O3	380.91	anticholinergic	synthetic	0
01503975	FLUCONAZOLE	C13H12F2N6O	306.27	antifungal	synthetic	0
01504152	NILUTAMIDE	C12H10F3N3O4	317.22	antiandrogen	synthetic	0
01500382	MELPHALAN	C13H18Cl2N2O 2	305.20	antineoplastic. alkylating agent	synthetic	0
01501175	FLURANDRENOLIDE	C24H33FO6	436.52	antiinflammator y	semisynthetic	0
01503017	AMINOTHIAZOLE	C3H4N2S	100.14	antithyroid agent	synthetic; RP-2921	0
01800018	LIMONIN	C26H30O8	470.52		*Citrus. Evodia. Dictamnus and Luvunga spp*	0
01500388	MESTRANOL	C21H26O2	310.44	estrogen. with progesterone as oral contraceptive	semisynthetic	0
01500105	ACETYLCYSTEINE	C5H9NO3S	163.19	mucolytic	synthetic	0
01500490	PIPERAZINE	C4H10N2	86.13	anthelmintic	synthetic	0
01500285	ESTRIOL	C18H24O3	288.39	estrogen	mammalian hormone	0
01500835	URSOCHOLANIC ACID	C24H40O2	360.58		*Abrus pecatorius*	0
01500373	MAPROTILINE HYDROCHLORIDE	C20H24ClN	313.87	antidepressant	synthetic	0

01500510	PROMETHAZINE HYDROCHLORIDE	C17H21ClN2S	320.88	antihistaminic	synthetic	0
01506035	REPAGLINIDE	C27H36N2O4	452.59	antidiabetic	synthetic; AG-EE-623-ZW	0
00330062	TEMEFOS	C16H20O6P2S3	466.47	insecticide	synthetic	0
01500190	CINOXACIN	C12H10N2O5	262.22	antibacterial	synthetic	0
00210477	ACTINONIN	C19H35N3O5	385.50	antibacterial	*Streptomyces spp.*	0
01506015	MEVALONIC ACID LACTONE	C6H10O3	130.14		common primary metabolite	0
00310040	alpha-TOCHOPHERYL ACETATE	C31H52O3	472.75	vitamin E	derivative	0
01500611	VINBLASTINE SULFATE	C46H60N4O13S	909.07	antineoplastic. spindle poison	*Vinca rosea*	0
01500191	CLEMASTINE	C25H30ClNO5	459.97	antihistaminic	synthetic	0
01500593	TRIMEPRAZINE TARTRATE	C22H28N2O6S	448.54	antipruritic	synthetic	0
01505030	DEMETHYLNOBILETIN	C20H20O8	388.37		*Citrus. Sideritis. Heteropappus and Thymus spp; Mentha piperita. Amaracus pampanini*	0
01506020	N-ACETYLMURAMIC ACID	C11H19NO8	293.27		bacterial cell wall component	0
01500715	ACETYLGLUCOSAMINE	C8H15NO6	221.21	antiarthritic	synthetic	0
01504151	NIMUSTINE	C9H13ClN6O2	272.69	antineoplastic	synthetic	0
01500145	BETAMETHASONE VALERATE	C27H37FO6	476.59	glucocorticoid	semisynthetic	0
01503935	PROPAFENONE HYDROCHLORIDE	C21H28ClNO3	377.91	antiarrhythmic	synthetic	0
01505840	STRYCHNINE METHIODIDE	C22H25IN2O2	476.36	neuromuscular blocker	derivative	0
01500866	HARMANE	C12H10N2	182.22	intercalating agent. sedative	*Arariba rubra.*	0
02300253	1R.2S-PHENYLPROPYLAMINE	C9H13NO	151.21	decongestant	Ephedra vulgaris (MaHuang)	0
01504221	PHENYLBUTYRATE SODIUM	C10H11NaO2	186.18	antihyperlipidemic	synthetic; R-757. Th-22	0
01500594	TRIMETHOBENZAMIDE HYDROCHLORIDE	C21H29ClN2O5	424.92	antiemetic	synthetic	0
01501148	SULCONAZOLE NITRATE	C18H16Cl3N3O3S	460.76	antifungal	synthetic	0
01500181	CHLOROTRIANISENE	C23H21ClO3	380.87	estrogen	synthetic	0
01500849	CHOLEST-5-EN-3-ONE	C27H44O	384.65		animal fats; mp 79-80 C	0
00100540	OBLIQUIN	C14H12O4	244.24		sneezewood or umtati. *Ptaeroxylon Obliquum*	0
01506078	MOROXYDINE HYDROCHLORIDE	C6H14ClN5O	207.66	antiviral	synthetic; SKF-8898A. ABOB	0
01505083	PRASTERONE	C19H28O2	288.43	androgen. estrogen. treatment of	adrenal glands. male urine	0

				menopausal syndrome		
01501146	SULFAGUANIDINE	C7H10N4O2S	214.24	antibacterial	synthetic	0
01503991	CLEBOPRIDE MALEATE	C24H28ClN3O6	489.96	antiemetic. antispasmodic	synthetic	0
01500456	OXYQUINOLINE HEMISULFATE	C9H9NO5S	243.23	antiinfectant	synthetic	0
01500381	MEGESTROL ACETATE	C24H32O4	384.52	progestogen. antineoplastic	semisynthetic	0
01500293	ETHOPROPAZINE HYDROCHLORIDE	C19H25ClN2S	348.94	antiparkinsonia n. anticholinergic	synthetic	0
00200013	ROTENONE	C23H22O6	394.42	acaricide. ectoparasiticide. antineoplastic. mitochondrial poison	Derris spp	0
01505880	DIFLORASONE DIACETATE	C26H32F2O7	494.53	antiinflammator y. glucocorticoid	semisynthetic; U-34865	0
01505316	PROTIONAMIDE	C9H12N2S	180.27	antibacterial	synthetic; TH-1321. RP-9778	0
01500629	FLOPROPIONE	C9H10O4	182.17	antispasmodic	synthetic	0
01500380	MEDRYSONE	C22H32O3	344.49	glucocorticoid	semisynthetic	0
01500583	TOLNAFTATE	C19H17NOS	307.41	antifungal	synthetic; SCH-10144	0
01501149	RITODRINE HYDROCHLORIDE	C17H22ClNO3	323.82	muscle relaxant (smooth)	synthetic	0
00330082	MITOTANE	C14H10Cl4	320.04	insecticide. antineoplastic	synthetic	0
01505909	1-HYDROXY-3.6.7-TRIMETHOXY-2.8-DIPRENYLXANTHONE	C26H30O6	438.52		Garcinia mangostana	0
01505015	3H-1.2-DITHIOLE-3-THIONE	C3H2S3	134.24	antioxidant. antineoplastic	synthetic; D3T	0
01505298	3-ISOBUTYL-1-METHYLXANTHINE (IBMX)	C10H14N4O2	222.24	phosphodiestera se inhibitor	synthetic	0
01505490	APIGENIN DIMETHYL ETHER	C17H14O5	298.29		common plant metabolite	0
01505869	AMPYRONE	C11H13N3O	203.24	analgesic. antiinflammator y. antipyretic	synthetic	0
01601020	DIETHYLTOLUAMIDE	C12H17NO	191.27	insect repellant	synthetic; DEET	0
01505395	BICUCULLINE(-) METHIODIDE	C21H20INO6	509.30	GABAa antagonist	semisynthetic; water soluble derivative of bicuculline (01500821)	0
01505157	2.2'-AZO-bis-2-AMINOPROPANE	C8H20Cl2N6	271.19	free radical initiator. apoptosis inducer	synthetic	0
01500842	CARYOPHYLLENE [t(-)]	C14H22	190.33		clove. cinnamon and many other oils	0
01505773	FAMPROFAZONE	C24H31N3O	377.53	analgesic. antipyretic. CNS stimulant	synthetic	0
00330018	DICHLORVOS	C4H7Cl2O4P	220.97	insecticide. cholinesterase inhibitor	synthetic	0

01500864	HARMALINE	C13H14N2O	214.26	CNS stimulant. antiparkinsonian agent	*Peganium harmala*	0
01504149	LEUCODIN	C15H18O3	246.30	antiinflammatory. hypolipidemic	*Artemesia & Achillea spp*	0
01500854	7-OXOCHOLESTEROL	C27H44O2	400.65		Cliona copiosa	0
01504209	DIPLOSALSALATE	C16H12O6	300.27	analgesic. antipyretic	synthetic	0
01505754	SEROTONIN HYDROCHLORIDE	C10H13ClN2O	212.68	neurotransmitter	CNS. GI tract. all animals. many mushrooms & plants	0
01500623	BROXYQUINOLINE	C9H5Br2NO	302.95	antiinfectant. disinfectant	synthetic	0
00240862	KUHLMANNIN	C17H14O5	298.29		*Manchaerium spp*	0
01505724	ISOFLUPREDNONE ACETATE	C23H29FO6	420.48	antiinflammatory	semisynthetic; U-6013	0
01504510	CHINIOFON	C9H5INNaO4S	373.10	antiprotozoal. amebicide	synthetic	0
01505990	CLEMIZOLE HYDROCHLORIDE	C19H21Cl2N3	362.30	H1-antihistamine	synthetic; AL-20	0

Table S2. List of 107 compounds selected by primary screening with an inhibition % against LsrK ≥70 %. Compounds were re-tested in singles at 10 μM against LsrK and at 50 μM against glycerol kinase.

Microsource ID	Compounds	Inhibition (%) against LsrK at 10 μM:	Inhibition (%) against LsrK at 50 μM:	Inhibition (%) against glycerol kinase at 50 μM:
1503118	TRIFLUPROMAZINE HYDROCHLORIDE	113	126	143
1505143	GOSSYPETIN	111	113	2
1504261	CANDESARTAN CILEXTIL	108	101	2
1503135	THONZYLAMINE HYDROCHLORIDE	105	115	2
1503074	ALEXIDINE HYDROCHLORIDE	105	143	64
1502253	HEMATEIN	103	105	3
1505151	HARPAGOSIDE	101	106	0
1505142	2'.5'-DIHYDROXY-4-METHOXYCHALCONE	96	100	1
300006	STICTIC ACID	93	116	0
1502032	SURAMIN	92	111	0
1504101	TETRACHLOROISOPHTHALONITRILE	91	135	1
1502252	MONOCROTALINE	91	109	0
1500572	THIMEROSAL	89	142	161
1500554	SULFINPYRAZONE	89	129	0
1500287	ETHACRYNIC ACID	87	86	0
1500328	HEXACHLOROPHENE	87	112	41
1505163	AURIN TRICARBOXYLIC ACID	87	94	2
1504019	GOSSYPOL	87	81	5
1500762	ROSOLIC ACID	86	117	1
1505775	AGARIC ACID	85	155	3
1500262	DISULFIRAM	82	155	0
201664	CELASTROL	82	107	0
200007	GAMBOGIC ACID	81	87	0
1500132	AUROTHIOGLUCOSE	81	104	178

1504082	DIHYDROCELASTROL	81	103	0
300549	ACETYL ISOGAMBOGIC ACID	80	138	1
1500637	MERBROMIN	79	120	147
100048	7-DEACETOXY-7-OXOKHIVORIN	78	131	0
201716	NORSTICTIC ACID	77	143	0
1500414	MINOCYCLINE HYDROCHLORIDE	77	71	1
1500436	NOREPINEPHRINE	77	74	0
201515	THEAFLAVIN DIGALLATE	77	94	0
1505707	TEICOPLANIN [A(2-1) shown]	76	152	0
1500266	DOXYCYCLINE HYDROCHLORIDE	76	106	0
1500517	PYRANTEL PAMOATE	76	101	18
1501118	MECLOCYCLINE SULFOSALICYLATE	76	93	0
1500636	MECYSTEINE HYDROCHLORIDE	76	110	3
201508	7-DESHYDROXYPYROGALLIN-4-CARBOXYLIC ACID	75	106	0
1505971	BENZBROMARONE	75	104	28
1505308	CHLOROPHYLLIDE Cu COMPLEX Na SALT	74	105	0
1501104	METHACYCLINE HYDROCHLORIDE	74	98	0
210242	THEAFLAVIN MONOGALLATES	73	127	0
1503941	THIOCTIC ACID	70	167	0
200012	BRAZILIN	69	131	0
201507	2'.2'-BISEPIGALLOCATECHIN DIGALLATE	69	167	0
1500365	LEVONORDEFRIN	68	124	3
1501198	TOLFENAMIC ACID	67	95	1
200054	FUMARPROTOCETRARIC ACID	67	147	0
1500345	HYDROXYZINE PAMOATE	60	125	22
1500838	CHOLECALCIFEROL	60	91	0
210239	EPIGALLOCATECHIN-3-MONOGALLATE	60	85	0
310035	SANGUINARINE SULFATE	57	84	56
1500268	DYCLONINE HYDROCHLORIDE	56	132	0
1500186	CHLORTETRACYCLINE HYDROCHLORIDE	55	77	0
1501111	PROTOPORPHYRIN IX	54	99	0
1504030	beta-ESCIN	53	126	7
1500450	OXIDOPAMINE HYDROCHLORIDE	51	117	0
1500148	BITHIONATE SODIUM	46	112	16
1500719	7.2'-DIHYDROXYFLAVONE	45	75	2
1503904	PATULIN	44	98	0
1500566	TETRACYCLINE HYDROCHLORIDE	42	114	2
1505390	NISOLDIPINE	40	159	0
1500455	OXYPHENBUTAZONE	39	94	0
1500457	OXYTETRACYCLINE	38	95	0
1505825	DIHYDROTANSHINONE I	38	113	0
1502020	FOLIC ACID	36	91	2
1500169	CETYLPYRIDINIUM CHLORIDE	35	132	30
211012	IRIGINOL HEXAACEATATE	35	112	0
1501150	SULPIRIDE	31	84	0
2300205	LEVODOPA	30	77	0
240673	ROBUSTIC ACID	28	109	0
201513	EPIGALLOCATECHIN 3.5-DIGALLATE	26	86	0
310016	CHAULMOOGRIC ACID	25	153	1
1500226	DEMECLOCYCLINE HYDROCHLORIDE	24	110	0
210238	EPICATECHIN MONOGALLATE	23	85	0
200111	THEAFLAVIN	22	130	0
1505177	RUBESCENSIN A	22	80	0
100013	3-DEACETYLKHIVORIN	21	97	0
200090	OBTUSAQUINONE	20	127	0
1503223	PARAROSANILINE PAMOATE	19	142	0

201505	METHYL 7-DESHYDROXYPYROGALLIN-4-CARBOXYLATE	17	110	0
205113	EPIGALLOCATECHIN	17	87	0
200488	NORSTICTIC ACID PENTAACETATE	17	85	0
201182	IRIGENOL	16	88	0
240565	5.7-DIHYDROXYISOFLAVONE	15	104	0
210515	PYROGALLIN	14	99	1
1500137	BENSERAZIDE HYDROCHLORIDE	14	97	2
1500603	TYROTHRICIN	13	77	2
200412	2.3.4'-TRIHYDROXY-4-METHOXYBENZOPHENONE	12	108	0
201448	4.4'-DIMETHOXYDALBERGIONE	12	96	0
200798	DALBERGIONE. 4-METHOXY-4'-HYDROXY-	10	88	0
300018	LOBARIC ACID	9	96	2
1600919	3-METHOXYCATECHOL	8	116	0
300147	USNIC ACID	8	200	1
201539	GARCINOLIC ACID	7	74	0
210505	PURPUROGALLIN	7	87	0
201281	DALBERGIONE	6	90	0
200033	LEOIDIN	4	82	0
10101011	BIXIN	2	77	0
200422	KOPARIN	2	115	0
300010	AGELASINE (stereochemistry of diterpene unknown)	2	100	0
200424	2.3.4-TRIHYDROXY-4'-ETHOXYBENZOPHENONE	1	78	0
300038	JUGLONE	0	86	0
1503610	BENZALKONIUM CHLORIDE	0	125	8
1505682	TOREMIPHENE CITRATE	0	142	0
1505412	BISMUTH SUBSALICYLATE	0	95	0
1500500	PRIMAQUINE DIPHOSPHATE	0	89	25

Table S3. List of 22 compounds selected for dose-response experiments against LsrK. For each compound, IC_{50} value and PCIdx are shown. Data points represent means ± SD from two independent experiments (n=2).

Microsource ID	Pubmed ID	Compounds	Structure	IC_{50}(μM)	SD	PubChem activity profile [a]	Promiscuity index (PCIdx) [b]
1504261	2540	CANDESARTAN CILEXTIL	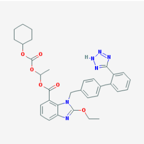	14	2	60/212	0.28
1504082	10411574	DIHYDROCELASTROL	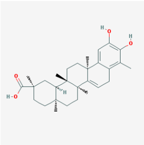	21	6		N/A
1503135	6136	THONZYLAMINE HYDROCHLORIDE	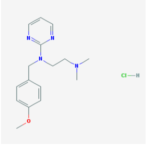	24	1	0/226	0
300549	6857789	ACETYL ISOGAMBOGIC ACID	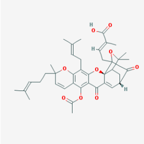	4	1		N/A
1505151	5281542	HARPAGOSIDE	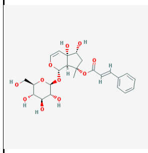	10	0	1/451	0
100048	6708526	7-DEACETOXY-7-OXOKHIVORIN	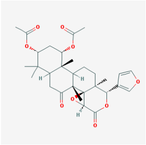	3	0.2		N/A
1505142	5355888	2'.5'-DIHYDROXY-4-METHOXYCHALCONE	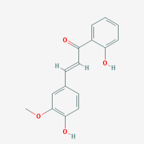	>50		9/80	0.11
1500436	439260	NOREPINEPHRINE	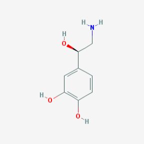	30	11	146/492	0.29
300006	73677	STICTIC ACID	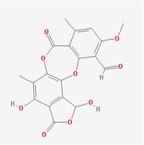	3	0.2	74/924	0.08

1500636	2733208	MECYSTEINE HYDROCHLORIDE	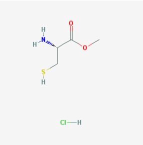	21	0.4	19/815	0.02
1504101	15910	TETRACHLOROISOPH THALONITRILE	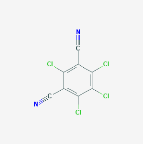	9	1	158/350	0.45
201508	5098885	7-DESHYDROXYPYROG ALLIN-4-CARBOXYLIC ACID	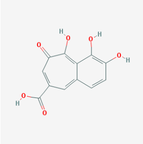	7	0.5		N/A
1502252	9415	MONOCROTALINE	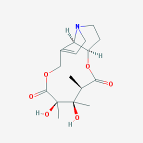	11	1	4/890	0
1500365	164739	LEVONORDEFRIN	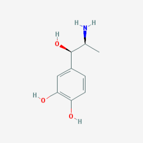	37	13	52/326	0.16
1505163	2259	AURIN TRICARBOXYLIC ACID	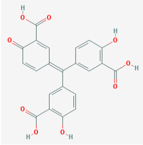	1	0.2	135/479	0.28
1501198	610479	TOLFENAMIC ACID	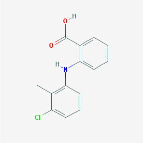	16	0.1	33/1159	0.03
1500762	5100	ROSOLIC ACID	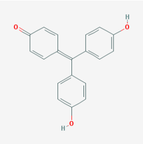	1	0.2	70/612	0.11
200054	5317419	FUMARPROTOCETRA RIC ACID	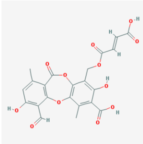	7	0.5	25/89	0.28
1505775	12629	AGARIC ACID	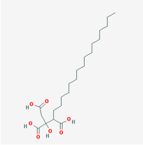	7	0.5	31/235	0.13
1500838	5280795	CHOLECALCIFEROL	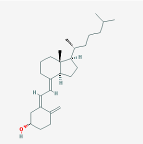	19	3	98/537	0.18

201664	122724	CELASTROL	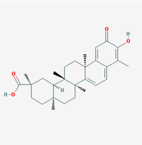	4	0.6	112/129	0.55
1501111	4971	PROTOPORPHYRIN IX	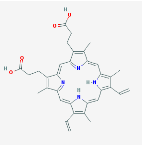	9	0.5	132/803	0.16

[a] Number of PubChem bioassays in which compound was reported as active/number of confirmatory bioassays reported in PubChem in which compound has been tested + number of screening bioassays reported in PubChem in which compounds has been tested

[b] Calculated according to Schürer et al.. J. Biomol. Screen. 2011; 16:415-426.

[c] Not calculated due to low number of bioassays in PubChem

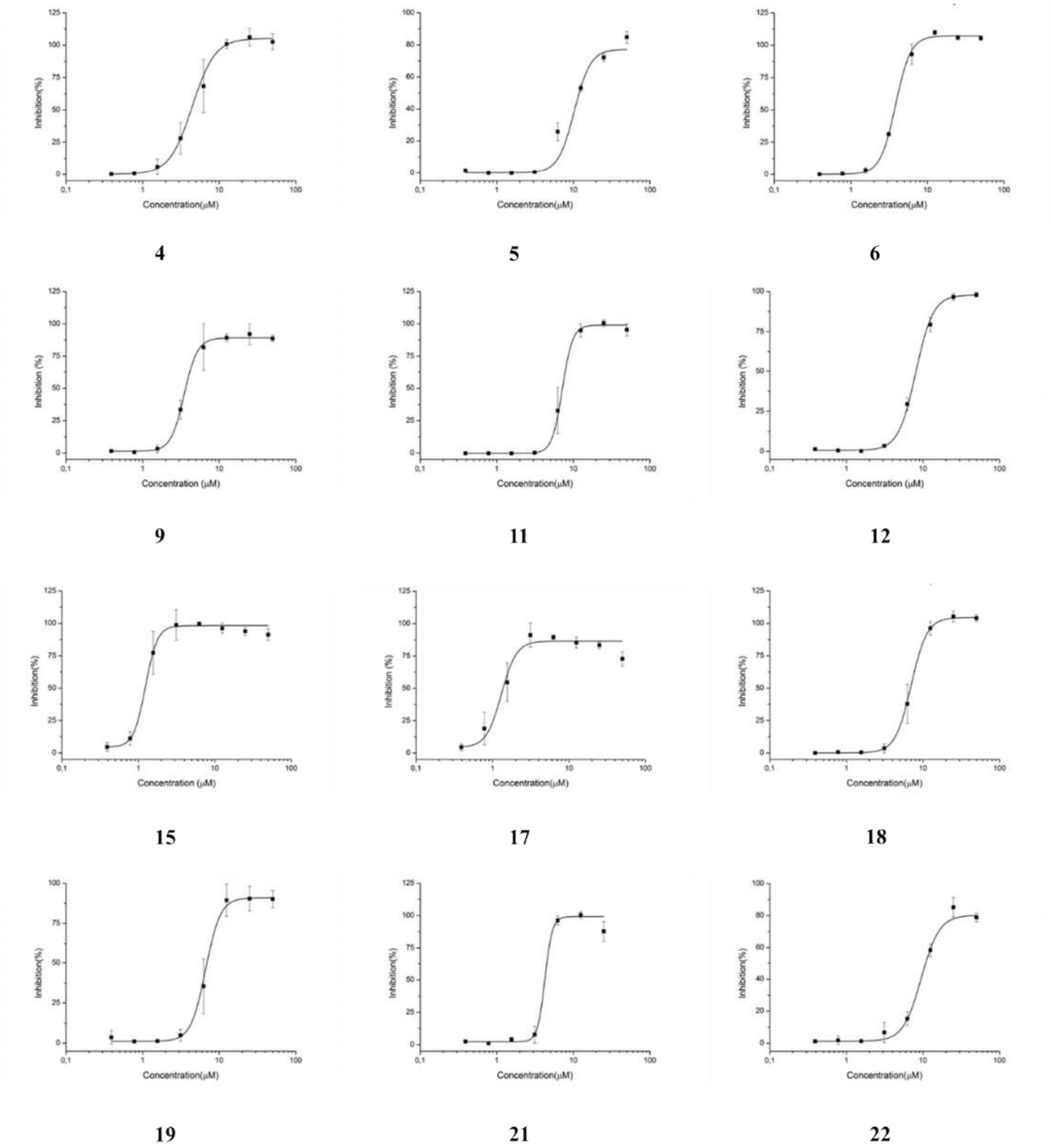

Figure S2: Dose-response curves against LsrK for compounds showing an $IC_{50} \leq 10$ μM. Data points represent means ± SD from two independent experiments (n=2).

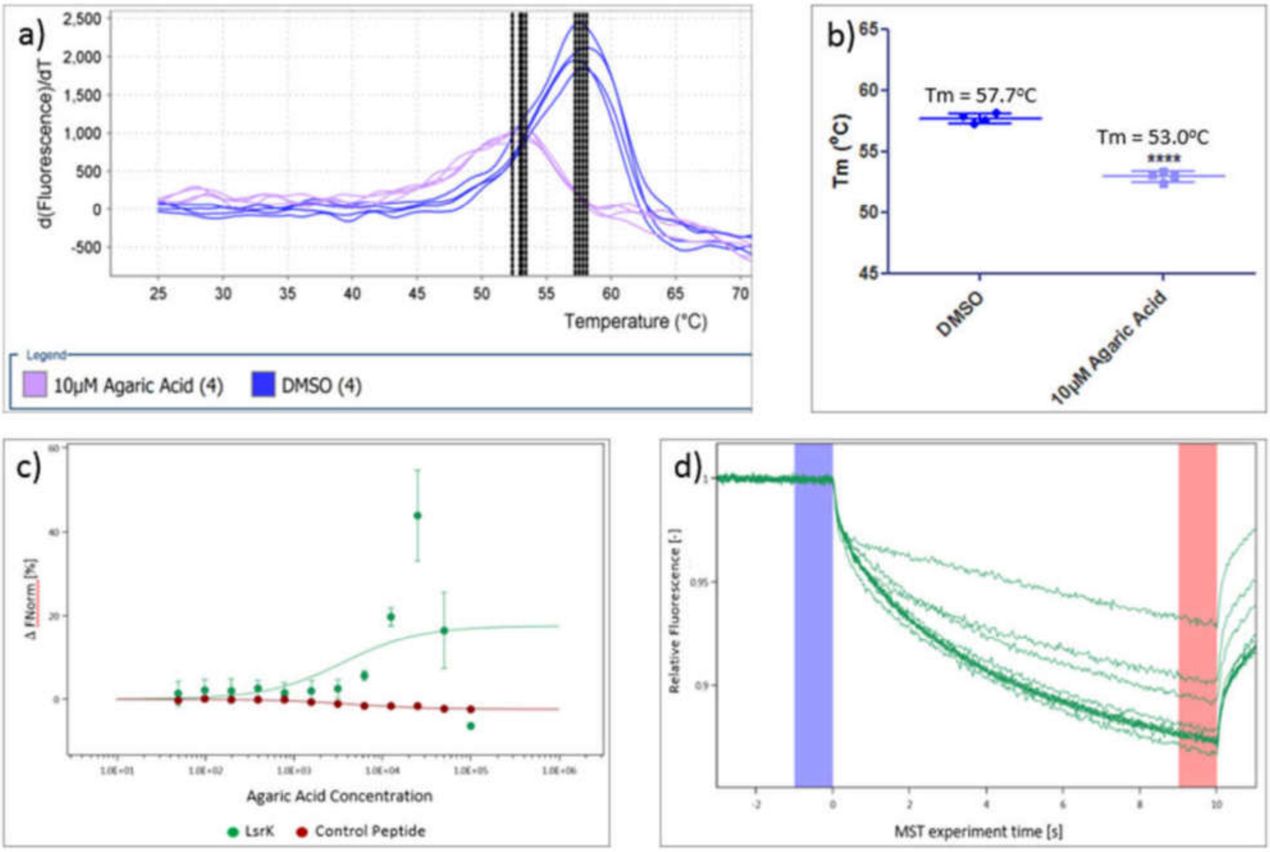

Figure S3. Biophysical profiling of the LsrK interaction with agaric acid: (a) derivative fluorescence in a thermal shift assay and (b) calculated Tm values mean ± SD (n = 3, **** two-tailed unpaired t-test [p < 0.001]). (c) Normalised changes in thermophoretic movement of dye labelled His-LsrK protein or His-Control Peptide in the presence of a dose range of agaric acid and (d) raw thermophoretic traces.

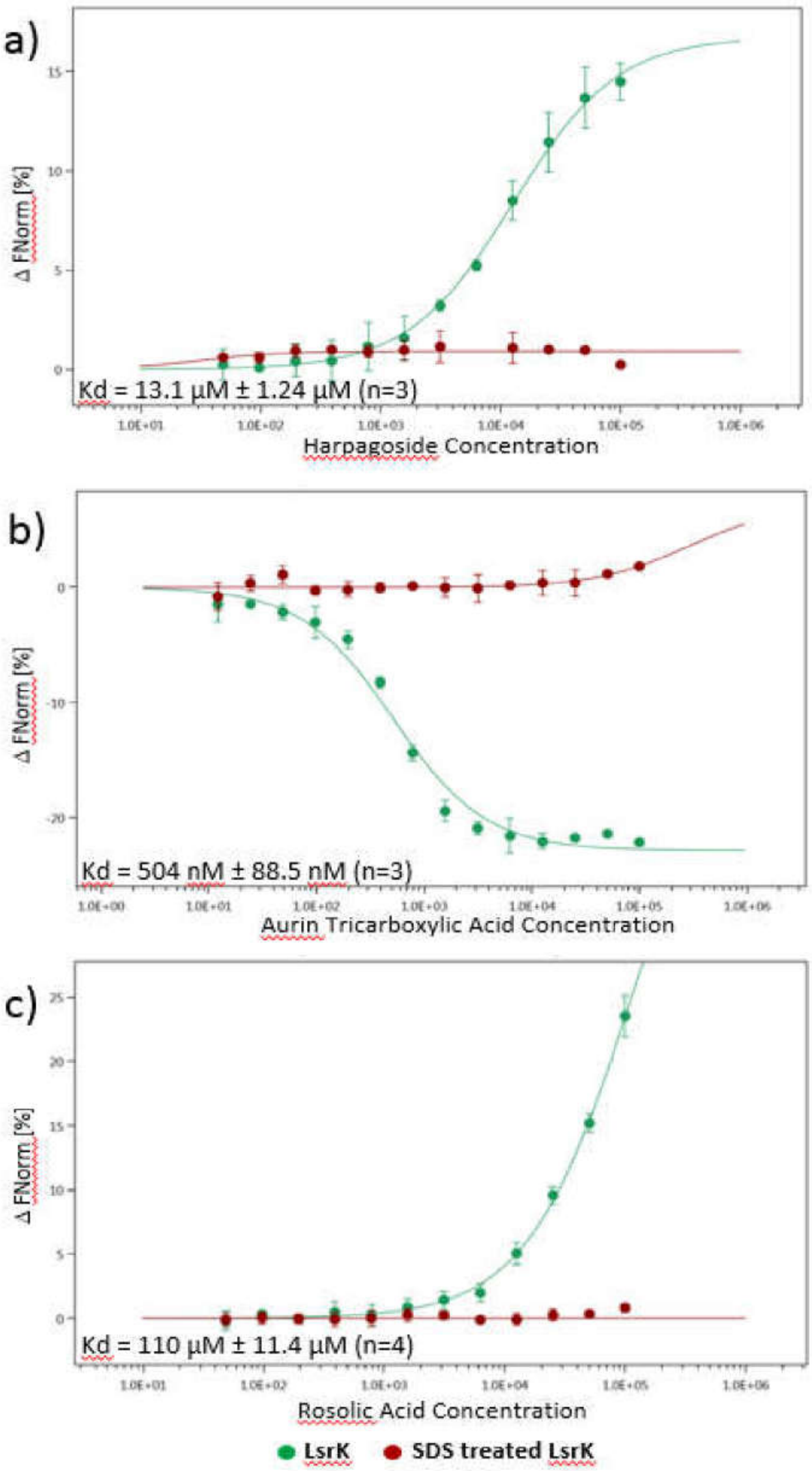

Figure S4. Normalised changes in thermophoretic movement in response to test compounds measured by microscale thermophoresis of dye labelled His-LsrK protein ± SDS. Kd values are calculated for each compound ± 95% confidence intervals.

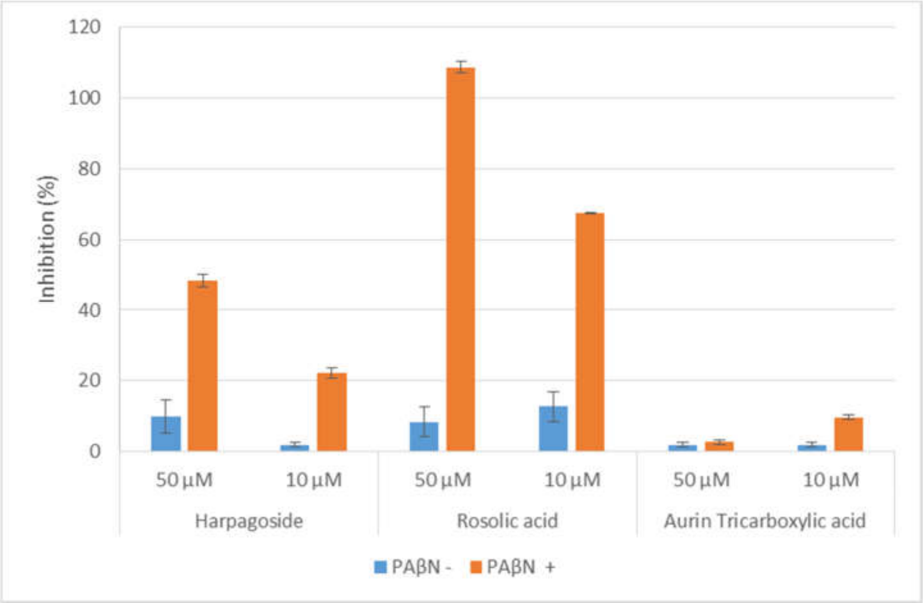

Figure S5. AI-2 QS antagonistic activity of harpagoside, rosolic acid and aurin tricarboxylic acid at 10 and 50 µM concentration determined by AI-2 QS interference assay with and without addition of PAβN (n=3).

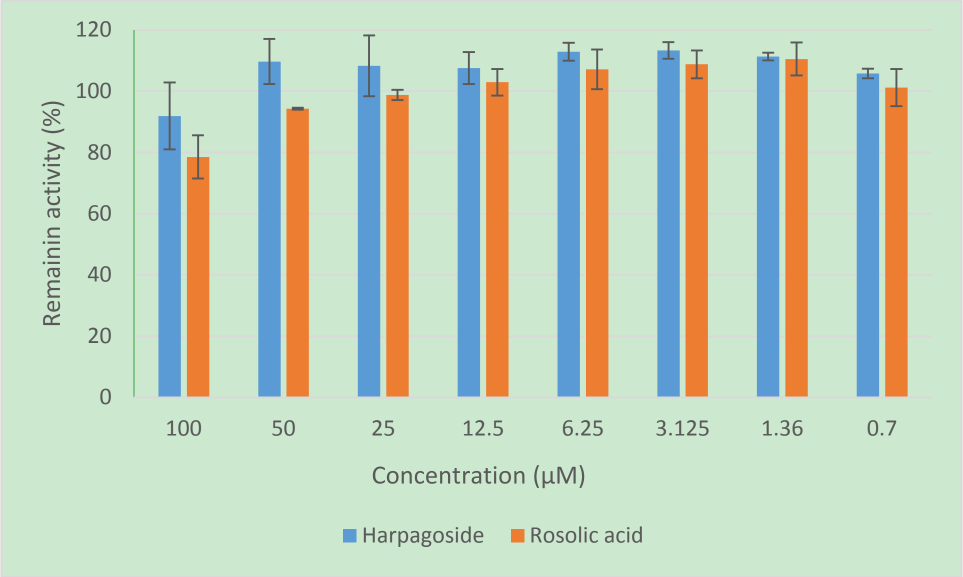

Figure S6. Unspecific response expressed as relative β-gal expression in the control strain *E. coli* pBAC-lacZ for AI-2 quorum sensing interference assay in presence of PAβN. Data points represent means ± SD from two independent experiments (n=3).
